# ConvexGating infers gating strategies from clusters in single cell cytometry data

**DOI:** 10.1038/s41467-026-77360-z

**Published:** 2026-09-18

**Authors:** Vincent D. Friedrich, Karola Mai, Thomas P. Hofer, Elfriede Nößner, Lorenzo Bonaguro, Celia L. Hartmann, Aleksej Frolov, Caterina Carraro, Doaa Hamada, Mehrnoush Hadaddzadeh Shakiba, Heidi Theis, Dalila Juliana Silva Ribeiro, Dagmar Wachten, F. Thomas Wunderlich, Markus Scholz, Fabian J. Theis, Matthias Becker, Marc D. Beyer, Joachim L. Schultze, Maren Büttner

**Affiliations:** 1 University of Leipzig, Institute for Medical Informatics, Statistics, and Epidemiology, Leipzig, Germany; 2 Center for Scalable Data Analytics and Artificial Intelligence (ScaDS.AI), Leipzig, Germany; 3 Systems Medicine, German Center for Neurodegenerative Diseases (DZNE), Bonn, Germany; 4 Modular High Performance Computing and Artificial Intelligence, German Center for Neurodegenerative Diseases (DZNE), Bonn, Germany; 5 Research Group Tissue Control of Immunocytes, Helmholtz Center Munich, Munich, Germany; 6 Life and Medical Sciences (LIMES) Institute, University of Bonn, Bonn, Germany; 7 PRECISE Platform for Single Cell Genomics and Epigenomics, DZNE and University of Bonn and West German Genome Center (WGGC), Bonn, Germany; 8 Immunogenomics & Neurodegeneration, German Center for Neurodegenerative Diseases (DZNE), Bonn, Germany; 9 Department of Microbiology and Immunology, The Peter Doherty Institute for Infection and Immunity, University of Melbourne, Melbourne, VIC Australia; 10 Molecular Immunology in Neurodegeneration, German Center for Neurodegenerative Diseases and the University of Bonn, Bonn, Germany; 11 Medical Microbiology and Immunology Department, Faculty of Medicine, Mansoura University, Mansoura, Egypt; 12 Institute of Innate Immunity, Biophysical Imaging, Medical Faculty, University of Bonn, Bonn, Germany; 13 Max Planck Institute for Metabolism Research, Center for Endocrinology, Diabetes and Preventive Medicine (CEDP), Cologne, Germany; 14 University of Leipzig, Faculty of Mathematics and Computer Science, Leipzig, Germany; 15 Institute of Computational Biology, Helmholtz Center Munich, Neuherberg, Germany; 16 Department of Mathematics, Technical University of Munich, Garching, Germany; 17 TUM School of Life Sciences Weihenstephan, Technical University of Munich, Freising, Germany

**Keywords:** Machine learning, Data processing

## Abstract

Manual expert gating remains common practice for defining specific cell populations in flow cytometry data, but increasing numbers of measured parameters and high inter-rater variability limit consistency across studies. Cluster-based approaches use the full marker space to define cell populations more consistently, but their outputs cannot be directly implemented on a cell sorter. Here we develop ConvexGating, an artificial intelligence tool to address this gap by automatically learning interpretable gating strategies for sorting in an unbiased, data-driven manner, generating low-contamination strategies for both known and previously unknown cell populations, including plasmacytoid dendritic cells identified solely as CD57-CD13-CD45RA+ CD123+ cells. We show that ConvexGating derives sorting strategies for CD8+ subtypes and adipose progenitor cell populations, which we validate experimentally by single-cell sequencing of sorted cells. We also demonstrate that the method transfers effectively to Cytometry by Time of Flight and Cellular Indexing of Transcriptomes and Epitopes by Sequencing data and improves marker panel design for cell sorting.

## Abstract (web summary)

Here, the authors develop ConvexGating, an AI tool that learns interpretable, data-driven gating strategies for cell sorting. They show that when analyzing scRNA-seq data, it yields low-contamination populations and also works across flow cytometry, cyTOF and CITE-seq data.

## Introduction

Flow cytometry is a widely used method for analyzing biological properties of single cells as well as sorting of cell populations for functional testing. Recent machine- and fluorochrome-wise technological advances enabled the measurement of up to 30 cellular markers in parallel. This leads to the acquisition of more and more extensive cellular signatures in flow cytometry experiments. In current mass cytometry (cyTOF) applications commonly 40 or more markers are used^1,2^ with a usually lower cell throughput than flow cytometry^2^. In sequencing-based readouts for antibody binding to cellular protein markers (e.g., CITE-seq), antibody panels utilizing even up to 200 different markers have been reported^3,4^. Consequently, the use of machine learning-based analysis methods is more common for high-dimensional mass cytometry and CITE-seq data^5^.

For the analysis of flow cytometry data, manual gating, a stepwise procedure of selecting cell subsets based on combinations of expression or intensities of characteristic cellular markers, is still common and best practice. In particular, one inspects the distribution of two selected markers and draws a gate around the population of interest in the corresponding two-dimensional space. Subpopulations within the gate are further distinguished by iteratively examining other marker pairs. This iterative approach builds on prior knowledge and yields a straightforward cell type definition. The problem of cytometric gating for cell type annotation can also be addressed with machine learning (ML). For instance, the UNITO approach classifies cell types with an image-based segmentation task in 2D, where optimal gate locations in a predefined gating hierarchy are learned via convolutional neural networks^6^. The manual gating approach for cell type annotation becomes increasingly laborious when up to 30 markers are analyzed. Furthermore, manual gating strategies are subject to variations across operators^7–9^ and over time for a single operator^9–11^ For instance, the separation of monocytes into classical, non-classical and intermediate subpopulations is often accompanied by considerable inter-rater variability^8^.

In contrast, clustering methods like PhenoGraph^12^ or Leiden clustering^13^, amongst others^14^, are most common for analyzing mass cytometry data, where panels encompass up to 40 markers. These unbiased approaches consider all markers simultaneously, which results in a more flexible separation of cells based on multiple features at once. In contrast to manual gating, clustering methods label all cells in a data set. To facilitate high-dimensional data analysis for flow or mass cytometry data, we previously developed the package *pytometry*^15^, which extends the popular single-cell framework scanpy^16^ and builds upon the annotated data frame (anndata)^17^ data structure and related Python packages from the scverse^18^. This integrative approach also paves the way to popularize machine learning applications for flow and mass cytometry data. Such more comprehensive analysis can better identify specific subpopulations and discover novel cell types that might be missed using traditional methods. For instance, ref. ^19^ analyzed cyTOF data from COVID-19 patients and identified disease-specific CD4+ and CD8+ T cells that expressed CD16 via clustering. The abundance of these T cells was associated with severe COVID-19 outcome. As a second example, basophils lose the basophil-defining marker CD123 upon activation^20^, which renders the recovery of basophils in the clinical basophil activation test more difficult. Accordingly, the discovery of novel cell types typically benefits from clustering to identify cell subtypes and states and further improves by contextualization with healthy reference data. In addition, clustering offers more flexibility to jointly analyze multiple samples at once, thereby facilitating multicenter studies.

Clustering also provides a more comprehensive view on the variability of innate immune cells. In particular, it was demonstrated that cell typing and lineage tracing benefit from high-dimensional data spaces and unbiased clustering outperformed manual gating-based analysis^21–23^. For example, the identification of plasmacytoid DCs (pDCs) can be compromised by precursors of conventional DCs (pre-cDCs) when only few markers are used^21,24,25^. As a result, a cell population sorted by flow cytometry could be affected by a potentially unknown level of cross-contamination. Such a population of cells may respond heterogeneously to a stimulus, either due to the inherent heterogeneity of the population or due to being a mixture of different cell types. Identifying the underlying cause for the differences in the response is then challenging.

Still, manual gating remains Gold standard, also due to the lack of complementary control experiments to determine true cellular identity. Recent advances on multimodal experiments like CITE-seq^3^, Abseq^4^, or REAP-seq^26^ combine surface antibody labeling and gene expression profiling at single-cell resolution. Such multimodal data provide independent information on cellular identity required to compare manual gating with clustering and other machine learning-based classification tools.

While using clustering to identify cell population has several advantages during data analysis, there is a considerable gap to transfer the cell identity back into a sorting strategy for further experiments. Such a translation of clusters to a sorting strategy requires reverse engineered gating strategies. An optimal gating strategy aims to recover the cells of interest (recall) with high purity (precision) in as few steps as possible. While clustering can inform the construction of a gating strategy, it largely depends on manual finetuning. This process can be significantly enhanced by ML approaches. Hypergate^27^ identifies populations via shrinkage and expansion of rectangles in the full marker space. Ji et al.^28^ present an approach for learning rectangular gate boundaries through gradient descent. However, certain subpopulations might require more flexible gate geometries than rectangles. Especially to avoid contamination with undesired non-target cells for small cell subpopulations, an arbitrary flexible gate shape in 2D marker space is beneficial. Enforcing a convex gate shape ensures a continuous gated region, thus sacrificing a certain level of performance for interpretability and reproducibility which can therefore be considered a form of regularization.

In this work, we present ConvexGating which derives full gating strategies for any cell population of interest in an autonomous fashion independent of manual expert gating. The suggested gating strategies aim at retrieving the cell populations of interest as much as possible while concomitantly avoiding contamination with non-target cells in a cell sorting experiment. Importantly, ConvexGating is designed to complement existing clustering or annotation tools by converting cluster-based cell type annotations into a hierarchy of 2D polygon gates that can be directly executed on a cell sorter. This closes the gap between computational analysis and physical cell isolation. ConvexGating outperforms existing tools for reverse gate engineering and works equally well with the full range of panel sizes from ten markers in FACS panels up to 200 markers in CITE-seq panels. We experimentally validate our approach with plate-based single-cell RNA-sequencing, where the ConvexGating-predicted gating strategy outperformed a conventional manual gating strategy for CD8+ TEMRA cells.

## Results

### ConvexGating ML workflow mimics manual gating

**Fig. 1:**
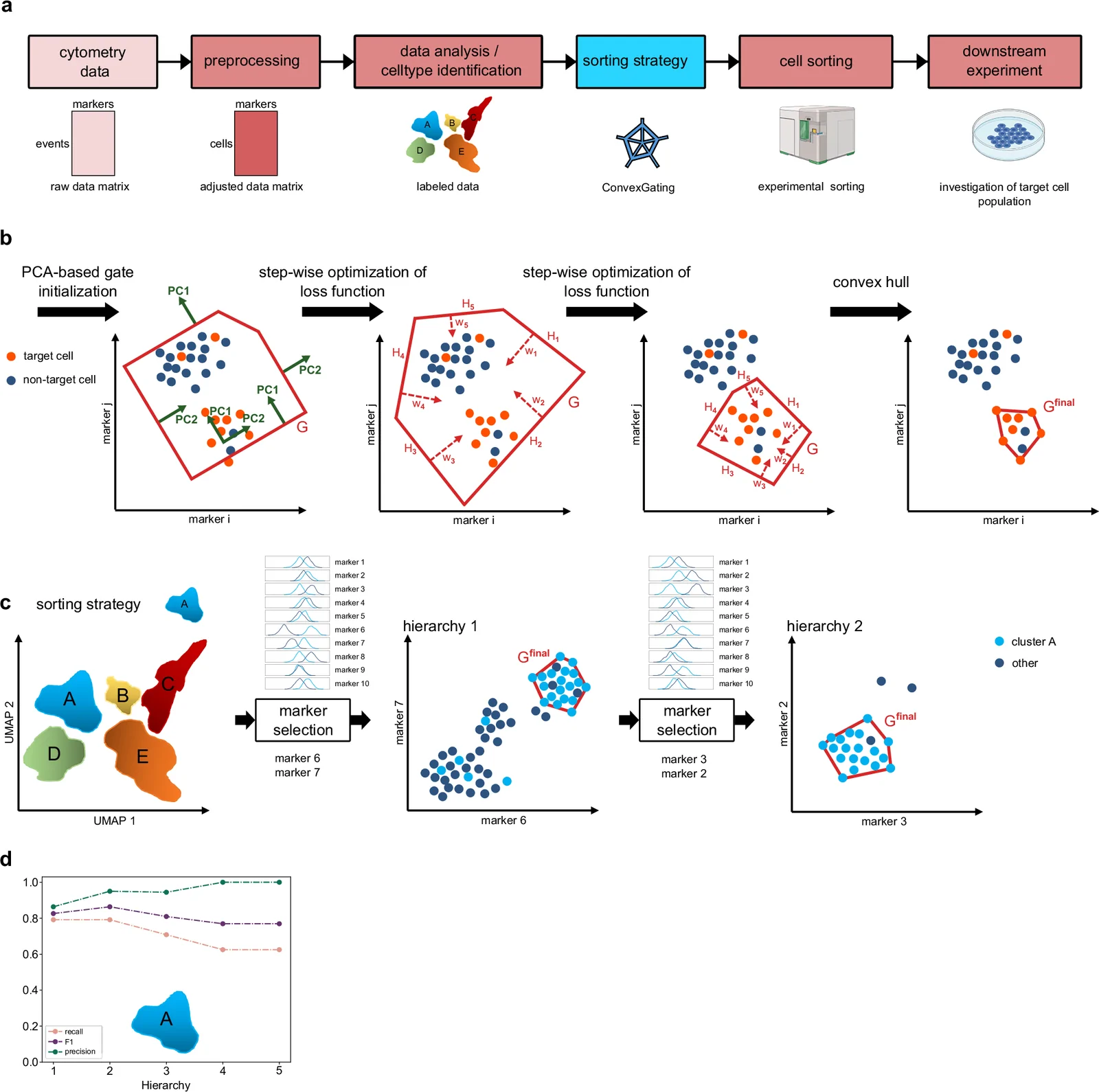
ConvexGating: a data-driven method for hierarchical and interpretable cell population gating. **a** Positioning of ConvexGating in a standard cytometry data analysis workflow. Created with BioRender.com (https://BioRender.com/h2ea5he). **b** Gate inference in 2D marker space. The gate is initialized partly at random and partly based on the first two principal components (PCs) derived from the target cell population, then refined through stochastic gradient descent (SGD)-based optimization of an explicitly designed loss function. The final gate corresponds to the convex hull of the SGD-optimized gate boundary. **c** Illustration of the algorithmic workflow for a sorting strategy targeting a cell subpopulation within a sample. At each hierarchy, marker selection is performed using a heuristic that quantifies the separability between the target and non-target cell populations. The gate is then learned in the corresponding 2D marker space, followed by new marker selection and gate inference in the subsequent hierarchy. **d** ConvexGating is evaluated by recovery of target cells (recall), by contamination with other cell types (precision), and by the weighted mean of precision and recall (F1 score). The maximum F1 score determines the number of hierarchies in the final gating strategy.

Manual gating is an iterative process, where users select a set of markers to separate a population of interest from the remaining cells based on prior knowledge. When using clustering to identify cell types, there is a considerable gap in translating such a cell type definition to an actionable sorting strategy for further experiments. In our approach, ConvexGating, we mimic manual gating in an automatic, fully data-driven way to compute gating strategies for cell sorting from cluster-based cell type labels. The inferred gating strategy can then be applied in a cell sorting experiment, which effectively separates gating as an analysis tool from gating as a cell sorting technique (Fig. 1a). Our approach is based on the assumption that every cell population in a flow or mass cytometry dataset can be identified by an appropriate clustering^12,13,29^.

We define the population of interest as the target population and refer to all other cells as the non-target population. In the first step, we calculate distribution parameters along each marker to select the most promising marker combination in terms of separability of target and non-target population in the current gating hierarchy (see Methods). When comparing marker rankings from this heuristic method with those from tree-based and SVM-based classifiers, we observed overall positive correlations, with the heuristic and SVM rankings being more strongly correlated than those from the tree-based approach (Supplementary Fig. 1a–c). The heuristic method also demonstrated the shortest computation time (Supplementary Table 1). Next, we represent the gate surrounding the target population as a convex shape bounded by a set of hyperplanes. One subset of hyperplanes is randomly initialized, while the normals of the remaining hyperplanes are fixed based on the principal components derived from the data matrix of the target population. During the optimization step, the hyperplanes form an intermediate gate by minimizing a modified version of binary cross-entropy loss complemented by two problem-specific weighted regularization terms using stochastic gradient descent. This loss incentivizes balancing recovery of target cells (recall) and purity of the gated population (precision). In the final step, we tighten the intermediate gate(s) to exclude as many non-target cells as possible. This is achieved by applying the convex hull to the encircled target population within the intermediate gate(s) (Fig. 1b).

We then repeat the process of obtaining an optimal marker combination and subsequent gate optimization until we obtain the cleanest possible population (high precision) and retain as many cells as possible (high recall) (Fig. 1c). ConvexGating prioritizes precision over recall, aiming for purity in the extracted population. We use F1 scores as a measure for the balance between precision and recall and monitor these metrics for all gating hierarchies. The final gating strategy retains hierarchies up to the level where the highest F1 score occurs (Fig. 1d).

### ConvexGating infers gating strategies similar to manual gating

**Fig. 2:**
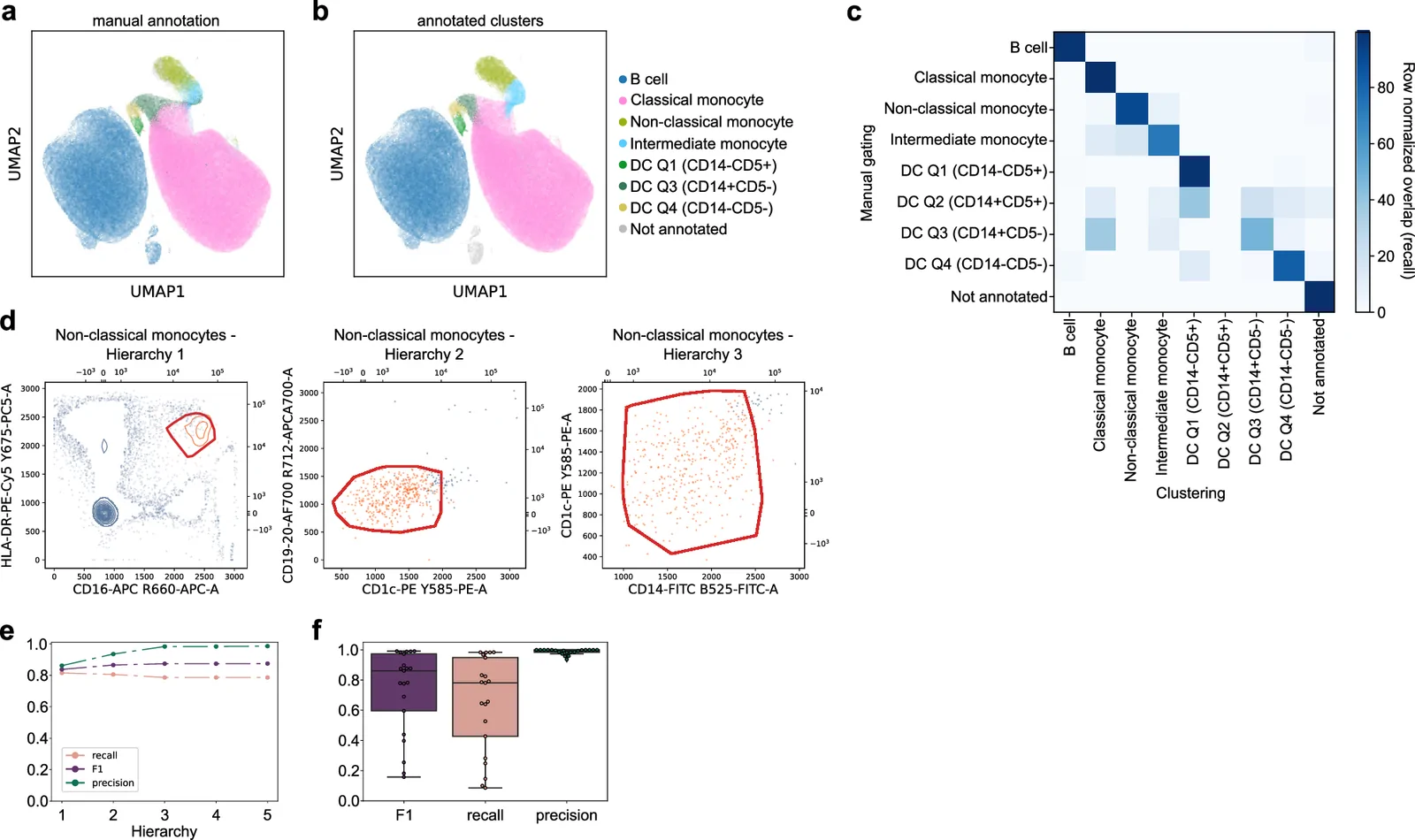
ConvexGating infers a gating strategy for annotated clusters on a monocyte panel. **a** UMAP displaying expert cell type annotation via manual gating. **b** UMAP displaying cell type annotation via clustering. **c** Row-normalized overlap (recall) of clustering with expert labels. **d** Gating strategy for non-classical monocytes inferred by ConvexGating. Target cells (orange) and non-target cells (blue) are represented via scatter plots and/or contour plots per target and non-target population. **e** Precision, recall and F1 score for each hierarchy in (**d**). No improvement in F1 score in higher hierarchies was observed. **f** Boxplot of precision, recall and F1 scores for all annotated cell populations, displayed is a summary of 21 observations (7 cell types × 3 samples). The independent experimental unit was the biological sample (*n* = 3); each sample contributed one metric value for each cell type, boxes represent 25th percentile, median and 75th percentile, respectively, whiskers show 1.5 * interquartile range. Source data are provided as a Source Data file.

In our first showcase, we demonstrate that cell type annotations achieved through clustering and manual gating are highly consistent, and inferred gating strategies successfully identify all target populations with high precision. In human blood, there are three dendritic cell (DC) populations classified as plasmacytoid DCs, CD141+ myeloid DCs, also termed cDC1, and CD1c+ myeloid DCs, also termed cDC2. Classification is not trivial, as there is no lineage marker. Consequently, markers are shared with other blood leukocytes, and marker expression depends on the activation state of the cells. Focusing on the CD1c+ DC2, there is a CD14+ and CD14- population^30^, and a differential expression of CD5 has been described^31^. Hence, there are at least two subsets of CD1c+ DC2 cells, which are CD1c+ CD5− CD14− cells, CD1c+ CD5+ CD14− cells, and CD1c+ CD5− CD14− cells. Current data suggest that the CD14− cells are bona fide DCs, while the CD14+ cells represent a unique population with ontogenetic links to monocytes^32^. Therefore, the correct dissection of the CD14+ and CD14− populations is of great importance. Care must be taken, as CD14 is expressed by all three monocyte subsets and the CD1c+ CD5− CD14+ DC2s tend to overlap with the classical monocytes. Also, CD14 is expressed at low levels by neutrophils, and CD1c+ is also expressed on B lymphocytes. To develop a robust identification strategy for the cDC2 subsets, we assembled a 6-marker panel including CD1c, CD5, CD14, CD16, CD19/20, and HLA-DR. We will refer to this panel as the DC panel in the following. In whole blood stainings from three healthy donors, we used both manual gating (Fig. 2a and Supplementary Fig. 2) and Leiden clustering with our previously developed Python package *pytometry*^15^ (Fig. 2b and Supplementary Fig. 3) to identify B cells (CD19/20+), monocytes (HLA-DR+, CD14+ or CD16+) and type 2 dendritic cells (cDC2s) (HLA-DR+, CD1c+). Notably, T cells remained unannotated as the panel did not include CD3 to stain T cells. We further subtyped classified monocytes into classical monocytes (CD14+, CD16−), non-classical monocytes (CD14low, CD16+) and intermediate monocytes (CD14+, CD16+) through clustering. For the identification of cDC2 subsets, we distinguished CD1c+ DC2s based on CD5 and CD14 markers as described above^32^. For both monocytes and cDC2 subtyping, we observed less overlap between manual gating and clustering, while the overall overlap with the manual gating annotation is high (see Methods) (Fig. 2c). In the case of monocyte subtypes, we attribute the lower concordance to the continuous decrease of CD14 and the increase of CD16, which makes the distinction of the subpopulations difficult. For cDC2s subpopulations, we work with relatively few cells, resulting in less concordance between manual gating and clustering.

We then applied ConvexGating to all cell types identified by clustering and examined the proposed gating strategies (Fig. 2d and Supplementary Fig. 4a–j). Here we showcase the ConvexGating results for the case of non-classical monocytes (Fig. 2d). The optimal gating strategy consists of three hierarchies and largely reflects the manual gating strategy (CD19/20-, HLA-DR+, CD16+, CD14−). In every hierarchy, we observe an increase in both F1 score and precision with final values of 0.98 for precision, 0.79 for recall and 0.87 for F1 score for this population (Fig. 2e). As optimized for highest precision, over all populations, our computational gating strategy has very high precision scores and for most cell types also high F1 scores (Fig. 2f and Supplementary Fig. 4d), and performance is stable when gating strategies are applied to unseen samples (Supplementary Fig. 5). Taken together, ConvexGating inferred precise and plausible gating strategies for abundant and rare cell types.

### Considerable variability exists across operators in a manual gating task

**Fig. 3:**
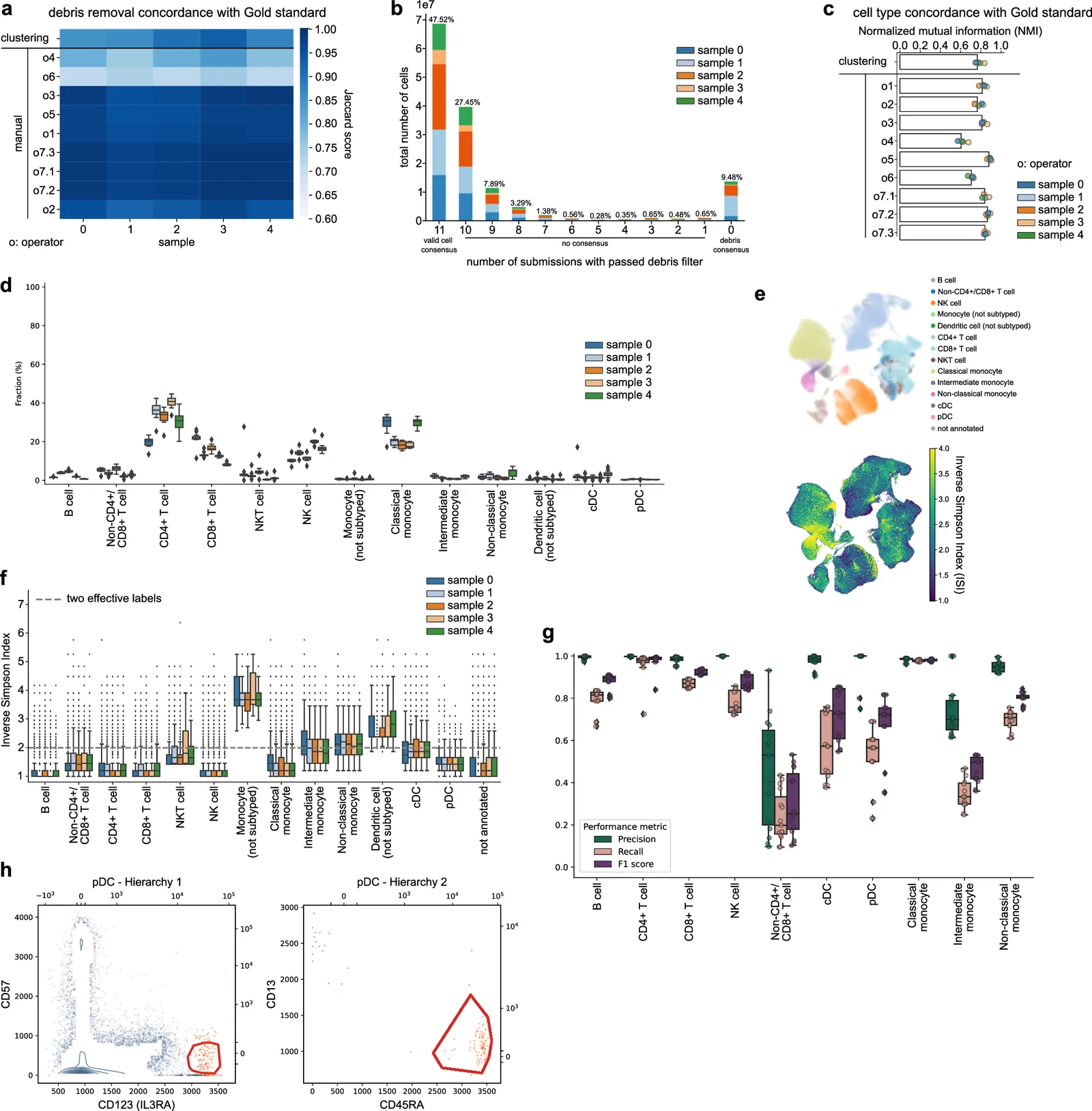
Assessing the variability of human expert-labeled data on a high-dimensional marker panel. **a** Heatmap depicting debris removal across all submissions compared to the Gold standard. Coloring indicates the Jaccard score over all cells per sample. **b** Stacked barplot on the number of cells labeled as valid, aggregated over all submissions, colored by sample. **c** Barplot of overlap of cell type labels compared to the Gold standard measured by normalized mutual information (NMI). Dots colored by sample indicate NMI per sample. **d** Boxplot of cell type composition over all submissions (*n* = 11 annotation submissions from seven individual annotators (one annotator submitted three annotations), plus the Gold standard and the cluster-based annotation), boxes represent 25th percentile, median and 75th percentile, respectively, whiskers 1.5 * interquartile range and black diamonds represent outliers, colored by sample. **e** UMAPs displaying the consensus cell type labels over all submissions and Gold standard (upper panel) and the number of effective labels per cell over all human expert labels measured by Inverse Simpson Index (ISI) (lower panel). **f** Boxplot of number of effective labels per cell over all submissions (*n* = 11 annotation submissions from 7 individual annotators (one annotator submitted three annotations), plus the Gold standard and the cluster-based annotation), boxes represent 25th percentile, median and 75th percentile, respectively, whiskers show 1.5 * interquartile range and black diamonds represent outliers), colored by sample, grouped by the consensus label over all submissions and the Gold standard. The gray dashed horizontal line indicates the effective number of two cell type labels. **g** Boxplots summarizing precision, recall and F1 scores per sample (*n* = 5 samples, boxes represent 25th percentile, median and 75th percentile, respectively, whiskers show 1.5 * interquartile range, and black diamonds represent outliers) obtained by running ConvexGating on all samples for cell populations annotated by clustering. **h** Two-step gating strategy for pDCs inferred by ConvexGating. Source data are provided as a Source Data file.

Next, we investigated the robustness of manually acquired gates on a larger flow cytometry panel with 27 markers using five frozen PBMC samples of healthy donors^33^. Specifically, we devised the task to gate for major cell types, such as T cells, B cells, NK cells and monocytes, as well as a finer resolution of T cell, monocyte and DC subpopulations (see Methods) (Fig. 3 and Supplementary Fig. 6a). We obtained a total of nine submissions from seven operators with different levels of expertise. As an additional submission, we preprocessed the same datasets computationally with debris removal (QC) and identification of cell populations with pytometry using Leiden clustering. We defined the gating result of the original study^33^ as the Gold standard for our comparison. To evaluate the concordance among operators, we first analyzed which cells are classified as debris (including doublets) and valid cells in the QC using FSC and SSC, and then measured the overlap with the Gold standard using the Jaccard score (Fig. 3a, see Methods). Seven submissions marked almost identical cells as valid cells, while two submissions were more restrictive, resulting in lower Jaccard scores. The computational QC recovered slightly less cells compared to the Gold standard (Fig. 3a and Supplementary Fig. 6b), but overlaps largely with the gating-based QC. In the Gold standard, around 89% of events passed QC as valid cells. Over all submissions, 9.48% of events were consistently labeled as debris (Fig. 3b), and we did not observe a QC bias over samples (Supplementary Fig. 6c).

Next, we quantified the robustness of cell type labels with several metrics. Firstly, we evaluated their overlap with the Gold standard using normalized mutual information (NMI) (Fig. 3c and Supplementary Fig. 6d, see Methods). The overlap of cell types and their compositions is largely consistent across all submissions (Fig. 3c), and the clustering results are within the range of observed variability of the results. Secondly, we computed the variability of cell type compositions (Fig. 3d and Supplementary Fig. 6e), and specifically compared the compositions obtained via clustering and by the Gold standard, respectively (Supplementary Fig. 6f, g). Overall, the deviation of clustering to the Gold standard lies within the variation observed over all submissions (manual gating) and samples, except for assigning slightly more cells to CD4+ and CD8+ T cells, while slightly less cells are labeled as classical monocytes. The discrepancy can be explained by the hierarchical approach of manual gating; the T cell gate is usually drawn first, while monocytes are identified thereafter. Monocytes ending up as contamination of the T cell gate cannot be re-assigned to the monocyte populations. This highlights the strength of high-dimensional clustering for cell type analysis as it reduces the bias of the hierarchical gating approach. Thirdly, to derive reliable gating strategies, we must ensure that the population of interest has a high purity and actually contains the cells of interest. However, some populations are inherently difficult to identify using gating. Therefore, we determined the number of effective cell type labels per cell using inverse Simpson index (ISI) (Fig. 3e, f and Supplementary Fig. 6h, i see Methods), where an effective number of 2 or more indicates discordance of the assigned labels across the expert gating strategies. In this assessment, we used the most often assigned label as a consensus label instead of relying on the Gold standard label alone (Fig. 3e). While the overall concordance of cell type labels measured using NMI is high (Fig. 3c and Supplementary Fig. 6d), we observe ISI of 2 and above in labeling DCs and the DC subpopulation cDCs, as well as intermediate and non-classical monocytes (Fig. 3e, f and Supplementary Fig. 6h, i). Some submissions labeled classical monocytes partially as NK cells, which is a common error in manual gating (Supplementary Fig. 6j, k). We conclude that the clustering-based cell type annotation is as accurate as a manual gating-based cell type annotation.

Next, we used the clustering-based cell type annotation as input for ConvexGating to infer gating strategies over all samples and determine the variability of the performance scores across the five samples (Fig. 3g and Supplementary Fig. 6l). The resulting gating strategies recover the majority of cell types with a precision of 0.95 or higher, except for T cells that are neither CD4+ or CD8+, and intermediate monocytes (Fig. 3g, and Supplementary Fig. 6m, n). ConvexGating shows a lower performance for some rare cell populations like intermediate monocytes (precision 0.71−0.9) and cDCs (precision 0.99−1 with F1 0.49−0.65). On the other hand, we observe a precision >0.9 for other rare populations like non-classical monocytes and pDCs across all samples. At the same time, we observed considerable heterogeneity in the assignment of cell type labels for intermediate and non-classical monocytes as well as cDCs (Fig. 3f), indicating that their recovery is also challenging for classical manual gating strategies. Notably, ConvexGating inferred a two-step sorting strategy for pDCs, which is independent of parent populations, and fully identifies pDCs using CD57- CD123+ in the first hierarchy and CD13− CD45RA+ in the second hierarchy (Fig. 3h).

### ConvexGating supports the optimal design of marker panels

To assess the applicability of ConvexGating to high-dimensional mass cytometry data for optimal gating strategies, we applied it to extract cell populations in eight healthy human bone marrow samples measured by mass cytometry^34^ with a 34-marker panel focused on T cell subtyping (“Oetjen cyTOF panel”, Supplementary Note 1 and Supplementary Fig. 7). With increasing cell type granularity, all metrics decrease slightly while the gating depth increases (Supplementary Fig. 7a–c), indicating the increasing difficulty in capturing T cell subpopulations. While ConvexGating used markers like CD4, CD8 and CD197 most often in the initial hierarchies (see “reduced strategy”), it used a greater variety of markers for later hierarchies (see “full strategy”), leading to a larger marker set than that classically used to identify the respective T cell subpopulations (Supplementary Fig. 7d, e). Moreover, ConvexGating directly infers a gating strategy for a target population, regardless of potential parent populations (Supplementary Fig. 7f, g). For example, the sorting strategy for CD4+ TEMRAs involves five steps with sufficiently high precision after three steps (Supplementary Fig. 7g, h). This underscores the higher flexibility of marker choice with ConvexGating and an increased efficiency, especially for sorting rare subpopulations directly, regardless of potential parent populations.

### ConvexGating strategies show better applicability across multiple scenarios compared to Hypergate

We benchmarked ConvexGating, the gating tool Hypergate^27^ and off-the-shelf SVM classifiers on three test scenarios (see Methods and Supplementary Note 2). Notably, while SVM classifiers do not provide directly translatable gating strategies for experimental cell sorting, they provide a theoretical performance estimate for the separation of target cells. ConvexGating showed higher mean F1 scores on the test set compared to Hypergate across all annotation levels and tested scenarios (Supplementary Fig. 8). ConvexGating focused on high precision while Hypergate prioritized high recall. Compared to SVM classifiers, we found that ConvexGating outperformed the linear SVM classifier on the DC panel and annotation level 2 of the large PBMC panel, while the linear SVM achieved a higher mean F1 score at annotation level 1 of the large PBMC and the cyTOF panels. ConvexGating also achieved a higher mean F1 score than the nonlinear RBF SVM at annotation level 2 of the DC panel; however, the RBF SVM showed the highest mean F1 scores in all other tested scenarios (Supplementary Fig. 8). The RBF SVM employs a nonlinear kernel transformation to project the data into a high-dimensional space where it creates a decision boundary separating the target from the non-target population. However, this boundary cannot be directly translated into a hierarchical gating strategy using simple geometric shapes such as polygons in two-dimensional marker space, which is essential for cell sorting. In contrast, ConvexGating is designed to provide gating strategies that are directly applicable to cell sorting experiments. This practical applicability comes at the expense of a certain degree of separation power compared to more complex classifiers, such as the RBF SVM.

### ConvexGating-sorted CD8+ TEMRAs show higher purity than conventionally sorted cells

**Fig. 4:**
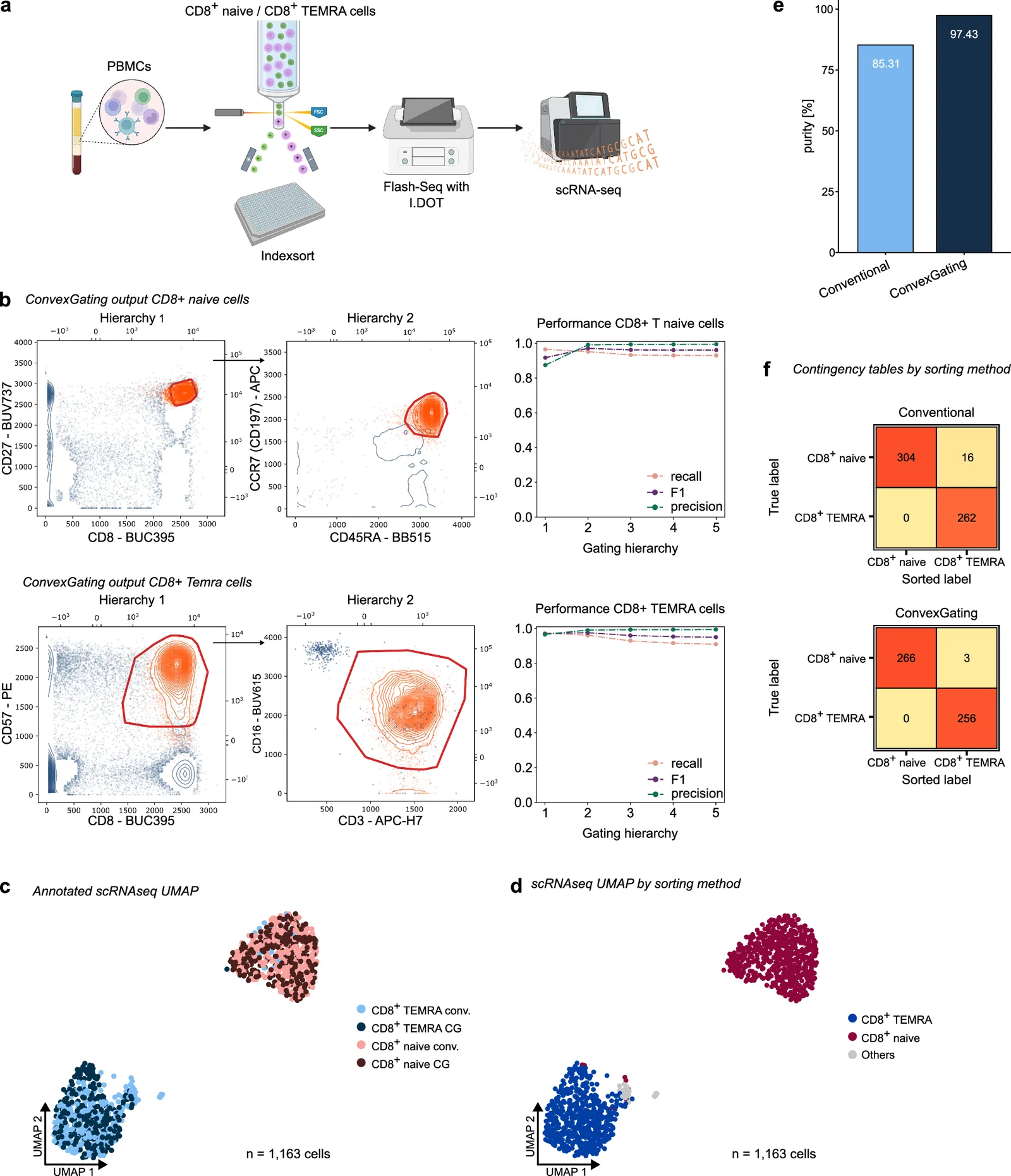
Experimental validation of purity in ConvexGating-sorted populations. **a** Experimental workflow for sorting CD8+ naive and CD8+ TEMRA cells with subsequent sequencing using the FLASH-Seq protocol to generate single-cell transcriptomes. Created with BioRender.com (https://BioRender.com/j37h536). **b** Sorting strategies predicted by ConvexGating for CD8+ naive cells (upper panel) and CD8+ TEMRA cells (lower panel) and their respective recall, F1 and precision metrics. **c** Uniform manifold approximation and projection (UMAP) of the transcriptome of single sorted CD8+ naive and CD8+ TEMRA cells, with colors indicating whether the cells were sorted by ConvexGating or by the conventional gating strategy. **d** UMAP plot showing cell type annotation based on T cell gene and protein markers. **e** Comparison of precision (purity) in the conventionally sorted and ConvexGating-sorted CD8+ TEMRA population. **f** Confusion matrices showing true positives, false positives, false negatives and true negatives for sorting CD8+ naive cells and CD8+ TEMRA cells using the conventional strategy (upper panel) and ConvexGating (lower panel). Source data are provided as a Source Data file.

To demonstrate the functionality of ConvexGating and to confirm the identity of the cells identified with ConvexGating, we performed a validation experiment sorting CD8+ naive and CD8+ TEMRA cells to confirm cell type identity on the transcriptional level using the FLASH-seq protocol^35^ (Fig. 4a and Supplementary Fig. 9a–c). For this purpose, we predicted a ConvexGating strategy based on a sample stained with 27 antibodies (Fig. 4b, referred to as the initial experiment). For comparison, we used a conventional gating strategy for subsequent sorting with the same 27 antibodies (Supplementary Fig. 9d). We index-sorted CD8+ naive and CD8+ TEMRA cells based on these two gating strategies into 384-well plates. We then followed the well-established FLASH-seq protocol to generate single-cell transcriptomes (see Methods).

After preprocessing, we clustered the cells (Supplementary Fig. 9e) and observed two clearly distinguished clusters: CD8+ naive cells and TEMRA cells (Supplementary Fig. 9f). The cells of the respective cell clusters appeared to be well-mixed in terms of sorting strategy (Fig. 4c). Next, we annotated the cells based on canonical T cell gene and protein markers (Supplementary Fig. 9g–i), and projected the labels onto the UMAP-embedding (Fig. 4d). One smaller cluster, sorted as CD8+ TEMRAs by the conventional gating strategy, did not express characteristic properties of either naive or CD8+ TEMRA cells and was therefore labeled “others”. Using differential expression analysis comparing conventionally sorted and ConvexGating-sorted cell populations showed negligible differences for both CD8+ naive and TEMRA cells (Supplementary Fig. 9j), confirming their identity.

To evaluate the specificity and sensitivity of ConvexGating, we compared the purities of the sorted populations obtained using both approaches. Our analysis revealed that ConvexGating resulted in a higher purity of CD8+ TEMRA cells than conventional sorting (Fig. 4e, f). In summary, we demonstrate that ConvexGating has similar or even higher precision for cell sorting.

### ConvexGating-sorted progenitor cells of white adipose tissue show higher purity than conventionally sorted cells

To demonstrate the applicability of ConvexGating-based gating strategies for cell sorting beyond immune cells, we performed a second validation experiment in visceral white adipose tissue (WAT), a technically challenging tissue for cell sorting due to its high lipid content and cellular fragility. We sorted two adipocyte precursor subpopulations (P1 and P2) and sequenced with FLASH-seq to confirm cell type identities (Supplementary Fig. 10). While P1 was challenging to sort in both conventional and ConvexGating-based strategies, we observed higher purity for the ConvexGating-based strategy for P2 compared to conventional sorting (Supplementary Fig. 11). In conclusion, ConvexGating successfully transfers to non-immune cells and maintains robust performance even in technically challenging tissues such as WAT.

### ConvexGating provides gating strategies for disease-specific, previously unknown cell populations associated with severe COVID-19 outcomes

**Fig. 5:**
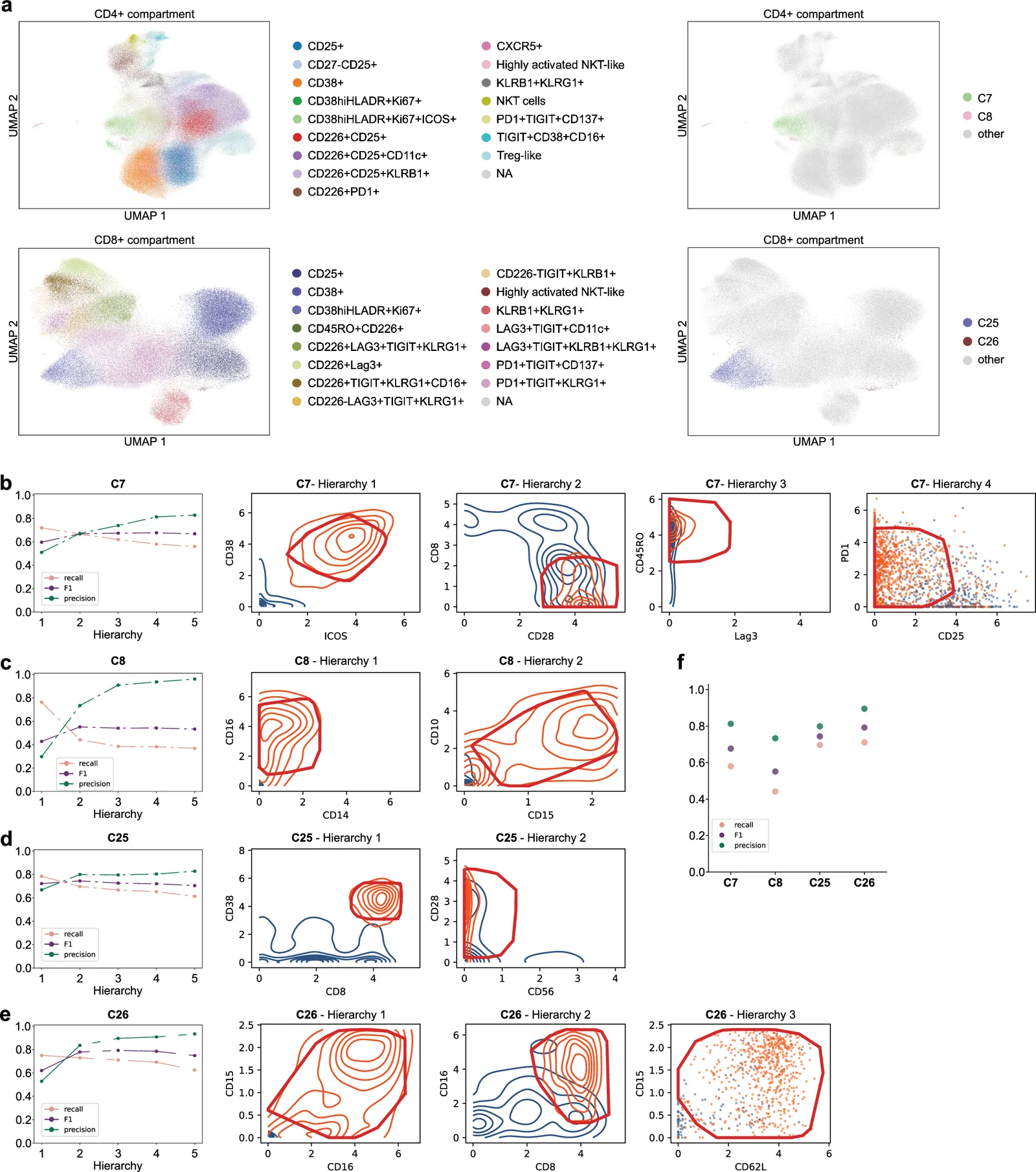
ConvexGating finds gating strategies for unknown populations in cyTOF COVID-19 data^18^. **a** UMAP representation of CD4+ T cell compartment (upper panels) and CD8+ T cell compartment (lower panels) with coloring according to subtype annotation (left panels) and clusters (right panels). **b**–**e** Performance scores and gating strategies for cluster 7 - CD4+ T cell compartment (**b**), cluster 8 - CD4+ T cell compartment (**c**), cluster C25 - CD8+ T cell compartment (**d**), and cluster C26 - CD8+ T cell compartment (**e**). Target cells (orange) and non-target cells (blue) are represented via scatter plots or contour plots per target and non-target population. Subsampling was performed to have a ratio of 1:15 between target cells and non-target cells. **f** Final performance overview on full cyTOF panel. Source data are provided as a Source Data file.

ConvexGating creates efficient strategies for previously unknown, ill-defined or perturbed cell populations (Fig. 5). To showcase this, we used the mass cytometry dataset of ref. ^19^ described CD16+ T cells in several independent cohorts of COVID-19 patients. CD16+ T cells had been previously described in patients with chronic infections and inflammation, but exhibited a more differentiated phenotype^36,37^. Specifically, the authors reported CD38high HLA-DR+ Ki-67+ subtypes (clusters 7 and 25) and CD3+ CD16+ highly activated NK-like cells (clusters 8 and 26) in both the CD4+ and CD8+ T cell compartments using cyTOF (Fig. 5a). They confirmed the existence of these populations via manually derived gating strategies. We applied ConvexGating on these populations to identify alternative gating strategies, reidentifying these previously unknown and rare populations (Fig. 5b–e). For the identification of cluster 7, ConvexGating selected CD38 and ICOS in the first hierarchy, and separated further with CD8- and CD28high (Fig. 5b). Interestingly, cluster 8 was previously separated from cluster 7 by adding ICOS to the gating strategy, while ConvexGating suggested a two-step strategy with the monocyte markers CD14 and CD16, and previously unused markers CD10 and CD15 (Fig. 5c). ConvexGating corroborated the previous finding where CD16 expression has been identified as a hallmark for activated T cell populations in both the CD4 and CD8 compartment, which was associated with severe outcomes in COVID-19^19^. Adding more hierarchies to the gating strategy strongly improved precision with almost constant F1 score (Fig. 5b–e, leftmost panels). Overall, ConvexGating provided a data-driven gating strategy with contamination estimates for disease-specific T cells for severe cases of COVID-19.

### ConvexGating bridges the gap between data modalities

**Fig. 6:**
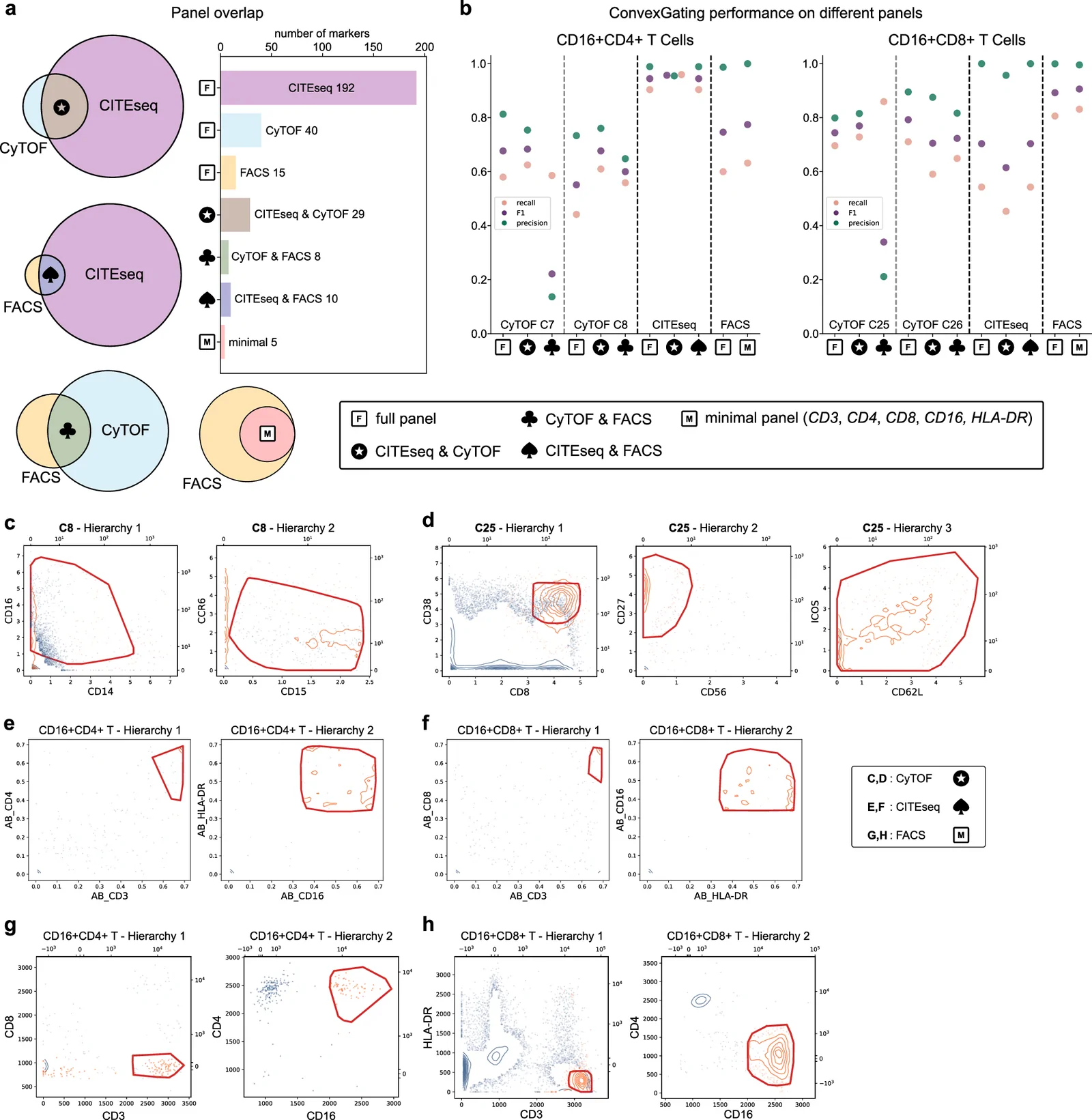
ConvexGating bridges the gap between cyTOF, CITE-seq and FACS antibody panels. **a** Overlap of cyTOF, CITE-seq and FACS antibody panels for independent cohort studies of COVID-19 using peripheral blood, highlighted through a Venn diagram showing shared and unique antibodies across methods, with a barplot detailing corresponding counts. **b** Performance overview (recall, F1 score, precision) for identification of CD16+ CD4+ T cells (left panel) and CD16+ CD8+ T cells (right panel) via ConvexGating on full, overlap and minimal antibody panels. (c-d) cyTOF: gating strategies for cluster 8 in CD4+ T Cell compartment (Fig. 4a) (**c**) and for cluster 25 in CD8+ T cell compartment (Fig. 4a) (**d**), both on the joint CITE-seq & cyTOF panel. **e**, **f** CITE-seq: gating strategy for CD16+ CD4+ T cells (**e**) and CD16+ CD8+ T cells (**f**), both on the joint CITE-seq & FACS panel. **g**, **h** FACS: gating strategy for CD16+ CD4+ T cells (**g**) and CD16+ CD8+ T cells (**h**), both on the minimal panel consisting of antibodies CD3, CD4, CD8, CD16, and HLA-DR. Target cells (orange) and non-target cells (blue) are represented via scatter plots and/or contour plots per target and non-target population. Subsampling was performed to have a ratio of around 1:15 between target cells and non-target cells (**c**–**h**). Source data are provided as a Source Data file.

To bridge the gap of sequencing-based cytometry to flow and mass cytometry, we examined ways to leverage large-scale panels as created with CITE-seq and demonstrate that the inferred marker choice can also be detected in flow cytometry or cyTOF data with a much smaller panel size (Fig. 6a). Here, we show the use of ConvexGating on three independent COVID-19 cohorts measured with CITE-seq^38^, cyTOF, and FACS^19^ to identify disease-specific CD16+ CD4+ and CD8+ T cells, respectively. For the CD16+ T cells in both the CD4 and CD8 compartments measured with cyTOF, we observed a fairly stable performance using overlapping markers of cyTOF and CITE-seq (Fig. 6b–d) and cyTOF and FACS, respectively, with the exception of clusters 7 and 25, where the overlapping markers of the cyTOF and FACS panel still allow to identify the population of interest with high recall, but high contamination, too (Fig. 6b). To identify CD16+ T cells in the COVID-19 CITE-seq panel, we first denoised the data using totalVI^39^, which integrates both single-cell RNA-seq and protein abundance data, and removes the background noise of the latter (Supplementary Fig. 12a–d). We subsequently identified a fraction of 0.07% CD16+ CD4+ T cells in the CD4 compartment and of 0.11% CD16+ CD8+ T cells in the CD8 compartment, respectively, which are predominantly observed in COVID-19 cases (Supplementary Fig. 12e). Using the full marker panel for each study individually, ConvexGating identified these cell populations with high precision (Fig. 6b). Using only overlapping markers of cyTOF and CITE-seq, and flow cytometry and CITE-seq, respectively, the performance to identify CD16+ T cells in both compartments remains stable, underscoring ConvexGating’s flexibility to identify target populations from marker subsets (Fig. 6b, e, f). For flow cytometry data, we defined a minimal marker panel (CD3, CD4, CD8, CD16 and HLA-DR) to identify our populations of interest. Surprisingly, ConvexGating’s performance even slightly increased on the minimal panel compared to the full marker panel (Fig. 6b, g, h). In summary, ConvexGating provides interpretable and simple gating strategies for disease-specific T cells across single-cell analysis platforms. Moreover, the performance metrics directly indicate what kind of purity level to expect, if those gating strategies are subsequently used for cell sorting and functional experiments on the full panel or on subsets of markers. ConvexGating handles different subsets of marker panels with stable performance. While we provide an export function for gating strategies, transferring them across modalities (FACS, cyTOF and CITE-seq) would most likely require manual adjustment. In addition, ConvexGating shows how well populations defined in complex panels can be gated in panels with fewer markers.

## Discussion

Machine learning applications have become increasingly popular for flow and mass cytometry data^15,40–42^. This development is accelerated by recent technological advances to increase the size of flow cytometry marker panels and the development of joint profiling of transcriptome and epitopes via, e.g., CITE-seq. Yet, in flow cytometry, manual gating is still the current Gold standard for both data analysis and sorting of cells of interest.

While an increased flow cytometry panel offers an enhanced characterization of single cells, it also makes the drawbacks of manual gating more apparent. In our study, we observed large inter-rater variation across operators, bias in cell type proportions due to the hierarchical nature of manual gating and inconsistent cell type label assignment especially in the DC compartment (Fig. 3). In contrast, clustering leverages the high-dimensional feature space to separate cell types and leads to very similar results as manual gating by an experienced operator (Figs. 2 and 3).

As such, data analysis via supervised, unbiased clustering^14^ or advanced ML techniques like automated cell type annotation using normalizing flows^43^ pose an alternative to manual gating. Cell sorting via FACS relies on hierarchical gating instead of clustering methods, such that tools like our proposed ConvexGating provide gating strategies in a data-driven, unbiased manner. Marker choice in ConvexGating depends on separability, such that markers have equal chances of being selected in the gating strategy, regardless of default usage in manual gating or frequent appearance in the literature. In contrast, the recently published UNITO approach^6^ provides segmentation masks in a 2D space similar to a conventional gate, but it relies on a predefined gating hierarchy. ConvexGating offers gating strategies optimized for cell sorting where minimized contamination is a prerequisite. This requires a high degree of precision in the extracted population while still capturing most of the target cells to obtain a comprehensive picture of the population under investigation. ConvexGating puts this into practice by aiming to maximize the F1 score, with precision implicitly given priority over recall. In terms of F1 score, ConvexGating outperforms the Hypergate gating tool^27^ across scenarios and different data modalities on the test set (Supplementary Fig. 8). ConvexGating provides short, high-precision gating strategies while Hypergate derives high-recall gating strategies with an increased risk for contamination. Consequently, we consider ConvexGating better suited for tasks where a high purity in the extracted population is desired. As ConvexGating is designed to optimize marker discrimination, marker selection is primarily guided by separability in feature space and consequently is not directly aware of biological significance but rather by panel characteristics such as signal brightness and staining index. This can occasionally result in a marker selection where, for example, a negative marker is selected to identify a more biologically informative positive marker. Consequently, ConvexGating may prioritize statistically informative markers that should be evaluated against domain knowledge when available, as the tool is expressly designed to support user-guided refinement.

Further, it must be noted that repeated mapping of high-dimensional marker expression data into a 2D marker space while striving for short, concise gating strategies is not optimal with respect to classification performance. More flexible ML models, such as nonlinear SVM classifiers, separate target populations with higher precision and recall (Supplementary Fig. 8). The higher performance comes at the cost of a lack of direct translatability into a gating strategy. Therefore, ConvexGating offers a trade-off between performance and practical applicability in cell sorting. Convex gate shapes induce contiguous, interpretable gated areas in 2D marker space while at the same time enabling more target population-adaptive and flexible gates compared to rectangular shapes or marker thresholding^21,22,27,28^. While complex classifiers may not provide directly implementable gating strategies, their insights into feature importance could inform future developments in automated gating tools, particularly for marker selection.

Due to its data-driven nature, ConvexGating supports the exploration and characterization of newly discovered or rare cell populations. The used markers indicate how the cell population can be efficiently extracted, regardless of whether the cell populations were originally identified via clustering or manual gating. The derived gating strategies then, in turn, allow drawing conclusions about the underlying biology of the cell populations under investigation (Figs. 5 and 6) and allow for optimal panel design. For high-dimensional human bone marrow data measured with cyTOF, ConvexGating provides high-precision strategies for both broad and fine-grained cell type definitions (Supplementary Fig. 7). Notably, rare T cell subsets require more markers for precise gating. At the same time, ConvexGating uses markers that provide the best separability for a population of interest. We demonstrated superior performance in terms of precision compared to conventional gating for CD8+ naive and TEMRA cells (Fig. 4 and Supplementary Fig. 9). Using index-sorting combined with an RNA-sequencing approach to investigate the identity of the sorted cells, we observed fewer contaminations in both populations. We observed similar improvements in cell sorting purity for two adipocyte precursor populations isolated from visceral WAT (Supplementary Figs. 10 and 11). This underscores the utility of ConvexGating-derived gating strategies in contrast to conventional ones and supports the operator of a flow experiment to obtain populations of interest with high purity.

In the disease context, ConvexGating is capable of deriving gating strategies for unknown cell subpopulations, e.g. the CD16+ T cell subpopulations in the CD4+ and CD8+ T cell compartments in patients with severe COVID-19 (Fig. 5). Here, ConvexGating helps with marker prioritization as a number of markers were found to describe the unknown population, and we showed that a minimal panel of four or five markers, respectively, is already sufficient to separate these cells. Large panels, as in cyTOF, spectral flow or CITE-seq experiments, provide a comprehensive picture of the cells under study, but translation into smaller panels while preserving performance might not be straightforward. In this scenario, ConvexGating can be used for optimal antibody choice and panel design as we can determine the performance of a smaller panel compared to larger panels computationally with direct estimates of potential contaminations (Fig. 6). Such an approach fosters the standardization of marker choice from extensive cellular signatures to effective sorting strategies. We demonstrated how ConvexGating can be used to create gating strategies for perturbed populations across multiple studies and modalities. However, a direct translation of a gating strategy would still require manual adjustment. We consider the translation of the inferred gating strategies from CITE-seq or cyTOF data to a flow cytometry or spectral flow experiment as a separate task since the data distribution in each modality is quite different, such that a reliable transfer of a gating strategy requires an invertible mapping into the same feature space. Finding such potentially nonlinear mappings is challenging because a reliable mapping of the same cells must be established, as well as a meaningful mapping of the convex gates from the integrated data space back into the original data space that is used for cell sorting.

In conclusion, ConvexGating links machine learning-based data analysis with cell identification by deriving gating strategies mimicking expert manual gating in an automated fashion. While some markers can be used interchangeably, our approach contributes to the efforts in standardization of cell type labeling and subsequent sorting. Our developed Python package integrates seamlessly with other popular single-cell analysis frameworks in Python to facilitate ML for flow and mass cytometry data. We envision that the explainability of the ConvexGating model and its output fosters an enhanced acceptance of our approach in biological and medical research, as well as drug discovery, leading to more efficient analysis pipelines that can adapt to expanding parameter spaces. In the future, we believe that machine learning tools like ConvexGating could lay the foundation for potential sample-specific automated live adjustments on cell sorters.

## Methods

### Ethical approval

Informed consent of whole blood of healthy donors (DC panel) was obtained in compliance with the Declaration of Helsinki and ethical approval was obtained from the Ethics Committee of the Ludwig-Maximilians University of Munich under the reference number 071-06-075-06. Ethical approval for the FLASH-seq experiment of human CD8 T cells was obtained from the Ethics Committee of the University of Bonn under the reference 272/23-EP. Ethical approval for the FLASH-seq experiment of mouse white adipose tissue progenitor cells was obtained from the Ethics Committee of the University of Cologne under the reference A2021.080.

### Staining of whole blood of healthy donors (DC panel)

Peripheral blood samples were collected from apparently healthy volunteers (Sarstedt S-Monovette EDTA K3E tubes, Cat. No. 04.1931). The antibody panel was designed to analyze monocytes and cDC2 subsets^32^. 100 µl of heparinized venous blood from three apparently healthy donors was used and subjected to erythrocyte lysis using Q-Prep (Coulter). After centrifugation, the cell pellet was resuspended in 100 µl staining-buffer (PBS without Ca^2+^/Mg^2+^ (Thermo Fisher, Cat. No. 14190144) supplemented with 2% FCS, 2 mM EDTA (Sigma, Cat. No. 6511), and 0.02% Na-Azide (Sigma, Cat. No. 102729748)) and cells were incubated with CD1c-PE (Miltenyi, Cat. No. 130-110-536), CD5-BV711(Becton Dickinson, Cat. No. 563170), CD14-FITC (Beckman Coulter, Cat. No. 6603511), CD16-APC (BioLegend, Cat. No. 302012), HLA-DR-PE-Cy5 (Beckman Coulter, Cat. No. A07793), CD19-AF700 (BioLegend, Cat. No. 302226), and CD20-AF700 (BioLegend, Cat. No. 302322) for 20 min in the dark on ice. After washing, the cells were resuspended in a staining buffer and analyzed using a Beckman Coulter CytoFLEX LX flow cytometer. A compensation matrix was generated using single-stained Versa Comp compensation beads (Beckman Coulter, Cat. No. B22804) and manually adjusted (Supplementary Table 2). FCS files were analyzed using FlowJo v10.8. After gating on singlets plus HLA-DR+ non-B cells, monocyte subsets were defined based on expression of CD14 and CD16, and cDC2 subpopulations were identified using CD1c, CD14, and CD5^32^ (Supplementary Fig. 2).

### FLASH-seq experiment of human CD8 T cells

#### Isolation of human PBMCs to sort CD8+ naive and CD8+ TEMRA cells

We isolated PBMCs of one healthy donor from EDTA-treated (tubes: Sarstedt, Cat. No 02.1066.001) peripheral blood via Pancoll (PAN Biotech, Cat. No. P04-60500) density centrifugation at 800×*g* for 20 min at room temperature without brake. Cells were collected with a Pasteur pipette and washed twice with MACS buffer (1x PBS (Gibco, Cat. No. 14190-144) with 0.5% BSA (Sigma, Cat. No. A7906-100G) and 0.074% EDTA (Invitrogen, Cat. No. AM9260G)). Then, we stained the cells with the surface antibodies listed in Supplementary Table 3 at 4 °C for 30 min. We washed the cells twice with MACS buffer and proceeded with FACS sorting.

#### Library preparation for CD8+ naive and CD8+ TEMRA cells

We FACS-sorted stained CD8+ naive and CD8+ TEMRA cells directly into 384-well plates (Eppendorf, Cat. No. 30128508), pre-filled with 1 µL of lysis buffer containing 0.2% Triton X-100 (Sigma Aldrich, X100-100ML), RNase inhibitor (Takara Bio Europe SAS, Cat. No. 2313 A), dithiothreitol (Thermo Fisher, Cat. No. 18090050), betaine (Sigma Aldrich, Cat. No. B0300-1VL), and supplied with dNTPs (Carl Roth, Cat. No. K039.2), SMART-dT30VN primer, dCTP (Thermo Fisher, Cat. No. 10217016), and FLASH-seq-Template Switching Oligo (all oligos were IDT custom orders, FS-TSO: 5′-Bio-AAGCAGTGGTATCAACGCAGAGTACrGrGrG-3′), using the FACSDiscover S8. Plates were sealed, snap-frozen on dry ice and stored at −80 °C until further processing. We prepared libraries following the FLASH-seq protocol as described by Picelli and Hahaut (2023)^35^ with minor modifications. Specifically, after a 3-min incubation at 72 °C, reverse transcription polymerase chain reaction (RT-PCR) was performed by adding 4 µL of RT-PCR mix containing Superscript IV (Thermo Fisher Scientific, Cat. No. 18090050) to each well using the I.DOT Non-Contact dispenser (Dispendix). The RT-PCR program consisted of: 60 minutes at 50 °C, 3 min at 98 °C, followed by 21 cycles of (20 s at 98 °C, 20 s at 67 °C, and 6 min at 72 °C). Amplified products were purified using the C.WASH noncontact centrifugal plate washer (CYTENA) for the beads (Beckman coulter, Cat. No. A63880) clean-up step. After plate normalization by adding nuclease-free water to, in average, a final concentration of 200 pg/µL, 0.5 µL cDNA underwent tagmentation and enrichment using the Tagmentase (Tn5 transposase- unloaded, Diagenode, Cat. No. C01070010). Loading of the Tn5 was conducted as described by ref. ^44^. In brief, Tn5 transposase was loaded with custom mosaic-end adapters. Two adapter mixes were prepared by annealing equimolar oligos: Tn5ME-A (5′-TCGTCGGCAGCGTCAGATGTGTATAAGAGACAG-3′) with Tn5MErev (5′-[phos]-CTGTCTCTTATACACATCT-3′), and Tn5ME-B (5′-GTCTCGTGGGCTCGGAGATGTGTATAAGAGACAG-3′) with Tn5MErev. Oligos were annealed by heating to 85 °C and cooling to 20 °C at −1 °C/min, then stored at −20 °C. For loading, 5 µL Diagenode Tn5 was mixed with 2.5 µL of each annealed adapter mix and incubated at 23 °C for 30 min. After the addition of 10 µL glycerol, the loaded Tn5 complex was mixed gently and stored at −20 °C until use. For preparing the final sequencing libraries, a working dilution of 0.01 µM was employed; however, optimization might be required depending on the specific activity of each batch of Tn5. The final libraries were quantified using a Qubit Fluorometer with the Qubit dsDNA HS Assay (Thermo Fisher), and the fragment size distribution was measured using the Agilent high-sensitivity D1000 assay on a TapeStation 4200 system (Agilent Technologies). Sequencing was performed in paired-end mode (101 cycles) on a NovaSeq 6000 system (Illumina) with NovaSeq S1 v1.5 (200 cycles) chemistry. Raw sequencing data were demultiplexed using bcl2fastq2 v2.20.

### FLASH-seq experiment of mouse white adipose tissue progenitor cells

#### Isolation and processing of visceral adipose tissue

C57BL/6n male high-fat diet feeding from 3 to 20 weeks. Mice (C57Bl/6N background) were kept in IVC at a specific pathogen-free facility at 22–24 °C with a 12 h light-dark cycle with 45–65% humidity. Mice had ad libitum access to water and a high-fat diet (HFD; EF D12492-(I); Ssniff Diet). The isolation of progenitor cells from the stromal vascular fraction (SVF) followed the protocol outlined by ref. ^45^. In brief, inguinal white adipose tissue (WAT) from one C57bl/6n 20 week-old male high-fat diet-fed mouse was excised, finely chopped, and enzymatically digested using 2 mg/mL collagenase II (Merck, Cat. No. C6885-5G) and 15 kU/mL DNAse I (PanReac AppliChem, Cat. No. A3778) in PBS (Gibco, Cat. No. 14190-094) containing 0.5% bovine serum albumin (BSA; Sigma, Cat. No. A4647-500G) at 37 °C under continuous agitation. The enzymatic reaction was terminated by adding AT buffer (PBS with 0.5% BSA). Following digestion, the cell suspension was filtered through a 100-μm strainer (Miltenyi, Cat. No. 420302) and centrifuged at 500×*g* for 10 min. The supernatant, which contained mature adipocytes, was removed, while the pellet comprising the stromal vascular fraction was resuspended in red blood cell lysis buffer (BioLegend) and incubated for 2 min at room temperature. This lysis step was halted by the addition of AT buffer, followed by centrifugation at 500×*g* for 10 min. The isolated cells were subsequently kept in Omics Guard (BD, Cat. No. 570911) at 4 °C overnight. The next day, cells were washed, then stained with the surface antibodies listed in Supplementary Table 4 and incubated at 4 °C for 30 min. Then, index sorting on a BD FACSDiscover S8 was performed.

#### Library preparation for adipocyte precursor subpopulations P1 and P2

The library preparation for sorted adipocyte precursor subpopulations P1 and P2 followed the same FLASH-seq protocol as for the sorted CD8+ naive and CD8+ TEMRA cells, with the following modifications to implement FLASH-UMI-seq as described by Picelli and Hahaut (2023). In the lysis buffer, we replaced the SMART-dT30VN primer with the STRT-dT31 oligonucleotide (5′Bio-AATGATACGGCGACCACCGATCGT31-3’) and omitted the FLASH-seq-Template Switching Oligo (FS-TSO). For the RT-PCR step, we added 4 µL of FS-UMI-RT-PCR mix containing 0.092 µL TSO carrying UMIs and a 5-bp spacer (TSO-UMI, 5′Bio-AAGCAGTGGTATCAACGCAGAGTNNNNNNNNXXXXXrGrGrG-3′, where NNNNNNNN represents the UMI and XXXXX the 5-bp spacer; 100 µM, IDT), 0.05 µL Tn5-ISPCR forward primer (5′TCGTCGGCAGCGTCAGATGTGTATAAGAGACAGAAGCAGTGGTATCAACGCAGAGT-3′, 100 µM, IDT), and 0.01 µL DI-PCR-P1A reverse primer (5′-AATGATACGGCGACCACCGA-3′, 100 µM, IDT), along with Superscript IV (Thermo Fisher Scientific, Cat. No. 18090050). Additionally, the annealing temperature in the RT-PCR cycling was reduced from 67 °C to 65 °C. All other steps remained identical as described for the sequencing of human CD8 T cells.

### Data analysis

In the following, we describe how we carry out the preprocessing steps with pytometry^15^ and scanpy^16^. Let ${X}^{{{\mathrm{orig}}}}\epsilon \,{R}^{{{\mathrm{NxM}}}}$ be our data matrix that contains original measurement values stored as one FCS file per sample. N denotes the number of cells, and M denotes the number of markers. The data processing steps vary in every dataset and are described in the following sections. After our preprocessing steps, we refer to our data matrix as ${X}^{{{\mathrm{data}}}}\epsilon \,{R}^{{{\mathrm{NxM}}}}$. Unless stated otherwise, all analyses were carried out in Python v. 3.8 with scanpy v. 1.8.1, anndata v. 0.8.0 and pytometry v. 0.1.2.

#### Flow cytometry data of whole blood of healthy donors (DC panel)

The dataset of whole blood stains encompasses three healthy donors stained with six markers (see DC panel above). We load all data in Python (v. 3.8) using the pytometry package^15^ (v. 0.1.3). We convert the FCS files into the anndata^17^ format (pytometry.io.read_fcs). Next, we perform compensation to correct for potential fluorescent spillover across channels, i.e., by multiplying the inverse of the spillover matrix by the data matrix. Then, we employ the bi-exponential transformation for normalization of flow data with the following parameters: max value 10,000,000, negative 0, positive 6, width basis −250, and channel range 4096. We then subset the data using the manual singlet gate and perform Leiden clustering^13^ of the k-nearest neighbor graph directly on the data (scanpy.pp.neighbors)^16^ to group cells and annotate the clusters based on characteristic marker intensities (scanpy v. 1.8.2). In particular, we employ a split-and-merge strategy to annotate the cells at different levels of resolution. In the first round, we separate B cells, monocytes and DCs from all other cells, which we term “not annotated”. In the next step, we subset to monocytes and DCs, and recompute the k-nearest neighbor graph and Leiden clustering to identify intermediate and non-classical monocytes. We reiterate this procedure for the DCs to identify DC2 subsets and to separate the remaining classical monocytes. We use UMAP to represent the data with low-dimensional visualizations (scanpy.tl.umap). In the final step, we merge the resulting cell type annotation into broad categories (cell type level 1) and refined categories (cell type level 2). To check the coherence of manual gating (Supplementary Fig. 2) and clustering, we visualize the final annotation as 2D scatter plots (Supplementary Fig. 3c–k).

We performed ConvexGating with standard parameters per donor and per cell type on a subset of 50,000 cells (scanpy.pp.subsample) for both cell type level 1 and cell type level 2 (Supplementary Fig. 4).

#### Flow cytometry data of frozen PBMCs of healthy donors (Knoll et al.)

Flow cytometry data were originally collected by ref. ^33^ using a 27 marker panel (see Supplementary Table S1 in Knoll et al.), and the data were shared with us by the authors. In this study, we use five samples collected from healthy donors. We load all data in Python (v. 3.8) using the pytometry package^15^ (v. 0.1.2). We convert the FCS files into the anndata^17^ format (pytometry.io.read_fcs). Next, we perform compensation to correct for potential fluorescent spillover across channels, i.e., by multiplying the inverse of the spillover matrix by the data matrix. We then remove doublets from the data via FSC-A/SSC-A and FSC-A/FSC-H channels. We then normalize with the bi-exponential transformation similarly to the original analysis with FlowJo (BD, v. 10.7.1). Unless otherwise stated, we used the following parameters: max value 262144.000029, negative 0, positive 4.41854, width basis −100, and channel range 4096. In the following, we will state the parameters that differed from the default values. For CD14 (UV A 810_40-A), we used max value 26214.400003, positive 3.41854, and width basis -50.118723. For CD197 (CCR7) (Vio A 780_60-A), we used a width basis of −10. For CD25 (YG A 780_60-A) and CD57 (Vio H 431_28-A), we used a width basis of −15.848932. For CD32 (UV H 379_28-A), we used max value 8289.72115, positive 2.91854, and width basis −25.118864. For CD45RA (Blue E 530_30-A), we used positive 4.55, and width basis -25.118864. For CD56 (Red B 730_45-A) and HLA-DR (Red A 780_60-A), we used a width basis of −39.810717.

We then perform Leiden clustering^13^ of the k-nearest neighbor graph directly on the data (scanpy.pp.neighbors)^16^ to group cells and annotate the clusters based on characteristic marker intensities with a similar split-and-merge strategy as described above using scanpy v. 1.9.1.

#### cyTOF data of human bone marrow (Oetjen et al.)

Leveraging previous work on the pytometry package^15^, we downloaded the publicly available flow and mass cytometry data of healthy human bone marrow donors^34^ from FlowRepository.org (accession codes: FR-FCM-ZYQ9, FR-FCM-ZYQB). We normalized the data with arcsinh-transformation and cofactor 5. We then use previously created cell type annotation based on Leiden clustering and marker intensities. The detailed analysis can be found at https://pytometry.readthedocs.io/en/latest/examples/.

We applied ConvexGating to retrieve gating strategies for cell populations in cyTOF data of human bone marrow. Gating strategies were separately learned on eight samples and four annotation levels (level 2–level 5) (Supplementary Fig. 7). In level 2, NK cells and T cells were annotated. With increasing annotation level, T cells were further subtyped (Supplementary Fig. 7a). We subsampled to 50,000 cells (scanpy.pp.subsample in scanpy v. 1.8.1) before applying ConvexGating with standard parameters. For the CD8+ TRM T cell population in sample B, we ensured a minimum number of 100 target cells after subsampling. We evaluated the performance of the gating strategies as described above. We further compared the performance of the full gating strategy with the performance of a reduced gating strategy that only relies on the two initial hierarchies. For all inferred gating strategies, we determined the optimal number of hierarchies and visualized the distribution per cell type annotation level (Supplementary Fig. 7b).

### FLASH-seq experiment of human CD8 T cells or mouse WAT cells

#### Data preprocessing and alignment

Single-cell data of either human CD8 T cells or mouse WAT tissue cells were demultiplexed using bcl2fastq2 v2.20. Following sequencing by the FLASH-Seq^35^, RNA-Seq libraries were subjected to initial quality control using FASTQC (v0.11.9) and multiQC (v1.14). Next, raw reads were pseudoaligned to the human transcriptome (hg38, Gencode 33) using Kallisto^46^ with default settings (v0.48.0) with an estimated average fragment length of 200 and an estimated standard deviation of fragment length of 30. The alignment pipeline was executed using SnakeMake v7.20.0.

#### scRNA-seq analysis of CD8+ naïve and CD8+ TEMRA cells

The transcript abundance files in hdf5 format^47^ from the pseudo alignment via Kallisto^46^ were merged and converted into Transcripts per Kilobase Million (TPM) normalized gene expression matrices, with additional length scaling (”lengthScaledTPM”) and otherwise default parameters, via the package tximport v1.20.0^48^. The Ensembl IDs were transformed into gene symbols using biomaRt v2.48.3^49,50^. The expression matrix and respective metadata were added to a Seurat object. Additionally, fluorescence intensity values from the index sorting to 384-well plates were exported from the index sort fcs files as csv files according to the manufacturer’s recommendation^51^ and were auto-logically transformed with the cyCONDOR^40^ package (v0.1.6). After transformation, flow cytometry index sort data was added to the merged Seurat object with dplyr v1.0.7. The Seurat object was further preprocessed by keeping only protein-coding genes. This process resulted in 15,608 genes left from the initial 33,806 genes. From the total 1536 cells that were sorted into plates, 1518 cells were sequenced. Singlets with fewer than 1500 detected genes or more than 30% mitochondrial gene counts were excluded from downstream analysis. Gene expression was normalized to the library size per well, multiplied by 10,000, and Log1p transformed, followed by centering and scaling^52^. The first 15 PCs were used for SNN and UMAP computation. The clustering was performed using the Louvain algorithm^13^. Clusters were annotated using canonical markers, and protein expression was detected using the index sort approach^51^. For visualization in the volcano plots, a threshold of *p* < 0.05 and a Log_2_ fold change >0.5 were applied to define differentially expressed genes.

#### scRNA-seq analysis of mouse adipocyte precursor subpopulations P1 and P2

For analysing mouse adipocyte precursor subpopulations P1 and P2, data were imported as described above for human CD8 T cells. Cells with fewer than 50,000 detected RNA counts or pseudolaignment rates below 25% were removed prior to downstream analysis. The final Seurat object consisted of 17,121 genes and 813 cells.

#### cyTOF data of T cells of COVID-19 patients

We downloaded the normalized and annotated cyTOF data set of T cells of COVID-19 patients^19^ from https://zenodo.org/record/5771937/files/data_Tcells_annotated.csv.gz. We then converted the data frame into an anndata^17^ object, separating marker intensities and metadata. Next, we selected the previously identified CD16+ T cell populations in the CD4 and CD8 compartments, which were annotated as clusters 7 and 8, and clusters 25 and 26, respectively. For each target cluster, we subsampled the anndata object to have an equal proportion of 6.25% target cells and 93.75% non-target cells (proportion 1:15). Furthermore, for cluster 7 and cluster 25 we subsampled the corresponding anndata object to 50,000 cells (scanpy.pp.subsample). We then performed ConvexGating with standard parameters.

#### Flow cytometry data of T cells of COVID-19 patients

We obtained the compensated flow cytometry data set of T cells of COVID-19 patients^19^ as an in-house data set with 15 markers. We convert the FCS files into the anndata^17^ format (pytometry.io.read_fcs). We then normalize the data using the bi-exponential transformation as previously used for analysis with FlowJo (BD, v10.7.1). Unless otherwise stated, we used the following parameters: max value 262144, negative 0, positive 4.41854, width basis -100, and channel range 4096. For CD3 (YG D 610_20-A), we used the width basis -79.432823. For CD16 (Vio E 605_40-A), max value was adjusted to 828972.114947. For HLA-DR (Vio H 431_28-A), we used width basis -31.622777. For CD19 (Red A 780_60-A) and CD203c (Red C 670_30-A), we used width basis -19.952623. Within the CD4 and CD8 T cell gates, to identify CD16+ T cells, we used a normalized CD16 marker intensity above 2000 similar to the CD16 threshold for neutrophils. The fraction of CD16+ CD4+ T cells is 0.048% of total CD4+ T cells and 2.54% of total CD8+ T cells.

For each target population, we subsampled the anndata object to have an equal proportion of 6.25% target cells and 93.75% non-target cells (proportion 1:15). Furthermore, for CD16+ CD8+ T cells, we subsampled the corresponding anndata object to 50,000 cells (scanpy.pp.subsample). We then performed ConvexGating with standard parameters.

#### CITE-seq data of the immune cell compartment in COVID-19 patients

We downloaded the annotated CITE-seq data set of the immune cell compartment of COVID-19 patients and healthy donors^38^ from https://www.covid19cellatlas.org/. The CITE-seq dataset consists of 624,325 cells with 24,737 expressed genes and 192 markers, respectively. In order to define CD16+ T cells, we selected the “raw” count data from the data set. We denoised the CITE-seq data using totalVI^39^ to account for the background signal of CITE-seq data. For cell type definition, we rely on the previously published cell type definition for CD4+ and CD8+ T cells (Supplementary Fig. 12a). We denote HLA-DR+ CD16+ T cells as T cells with a foreground probability of both HLA-DR and CD16 higher than 0.4 and being clustered with the CD4 and CD8 T cell compartments, respectively (Supplementary Fig. 12b–e). As part of the preprocessing pipeline, we utilize log+1 scaling for normalization of the protein expression data. Furthermore, we subsample the data for each cell type of interest, namely CD4 and CD8 T cells, to ensure a proportion of 6.25% target cells and 93.75% non-target cells before applying ConvexGating with standard parameters.

### Manual gating comparison

To assess the inter-rater variability of manual gating across operators, we devised the following manual gating task to be carried out by members of the lab. The test dataset was originally collected by ref. ^33^ and consisted of PBMC samples from five healthy human donors measured on a BD Symphony A5 instrument, encompassing 27 markers (Supplementary Fig. 6a), which covers the markers for all major immune cell populations. The dataset was compensated, but not filtered for debris. Every participant is trained in the use of a flow cytometer and in the analysis of immune cell populations using FlowJo (BD, v. 10.7.1). We obtained nine submissions from seven participants, where one participant submitted three different gating strategies. As the “Gold standard”, we defined the cell type annotation of the original study. All participants were asked to gate the following populations:

B cells, T cells (with the subtypes CD4+, CD8+ and NKT cells), NK cells, monocytes (with subtypes classical, non-classical, and intermediate), and DCs (with the subtypes pDC and cDCs). No further advice as to which gating strategy to use was given. Notably, the task was designed to cover abundant populations as well as more rare and difficult to gate ones.

All participants submitted their manual gating results in FlowJo as a FlowJo workspace (wsp) file. For analysis, we converted all gating results into a one-hot encoded data frame using CytoML v. 3.12 and flowWorkspace v. 3.13 in R v. 4.0.3 and exported the cell annotation data frames then as csv files for further analysis with pandas v. 1.3.5 and scanpy v. 1.9.1 in Python v. 3.8. We harmonized cell type labels into three levels: The “valid cell” level marks the first pass QC, where debris is removed from further analysis. Next, we defined the first level of cell type annotation as the cell types B cells, T cells, NK cells, monocytes, and DCs. The second level of cell type annotation encompassed, in addition, the subtypes of T cells, monocytes, and DCs, respectively. Cells that were not classified or failed QC were labeled as “not annotated”. In addition to manual gating, we analyzed the data using Leiden clustering in scanpy and added this annotation as “clustering” to the comparison (see data analysis).

We examined the concordance of cell type labeling using manual gating and clustering annotation with the Jaccard index, normalized mutual information, and inverse Simpson index (ISI), and determined the cell type compositions for all submissions. In addition to the Gold standard, we computed the most often assigned label as the “consensus label” as the most likely label of a cell to account for potential bias in the Gold standard. Unless stated otherwise, we compared cell type annotations to the Gold standard.

#### Jaccard index

The Jaccard index^53^ quantifies the similarity of two set ${S}_{1}$ and ${S}_{2}$ by considering its intersection over union.

1 $$J({S}_{1},{S}_{2})\,=\frac{|{S}_{1}\,\cap \,{S}_{2}|}{|{S}_{1}\,\cup \,{S}_{2}|}$$

It follows that $0\,\le {J}({S}_{1},{S}_{2})$ $\le \,$1. High values for J indicate high similarity of ${S}_{1}$ and ${S}_{2}$.

#### Normalized mutual information (NMI)

Mutual information (MI) between two label assignments of the same data quantifies the degree of similarity between the labels^54^. The NMI further maps the MI to the range [0,1], where higher NMI values indicate higher similarity between two sets of labels.

#### Inverse Simpson index (ISI)

Inverse Simpson Index (ISI) is a measure of the effective number of categories present in a population. It is based on the Simpson index (SI)^55^, which measures the diversity within a population. For a population P with N total elements, C different categories and ${n}_{c}$ elements per category, SI reads as

2 $${SI}(P)=\frac{{\sum }_{c}{n}_{c}({n}_{c}-1)}{N(N-1)}$$

We have that $0 < {SI}(P)\,\le \,1$. If ${SI}(P)=1$, all elements stem from one category while for ${SI}(P)=0$ each element is assigned to its own category. The higher the SI, the lower the diversity within population P. The ISI score is then obtained by taking the inverse of SI

3 $${{\mathrm{ISI}}}(P)=\frac{1}{{{\mathrm{SI}}}(P)}$$

It follows that $1 < {ISI}(P)\,\le {C}$. ISI reflects the effective number of categories in P. To determine the most likely label of a cell, we used the most often assigned label as “consensus label” to account for potential bias in the Gold standard. The lower the ISI for a cell type reflects a more consistent labeling across operators.

### Benchmark gating strategies

We conducted a comprehensive benchmark analysis of ConvexGating against the state-of-the-art automated gating tool Hypergate^27^. Hypergate fits a high-dimensional rectangle to separate target cells from non-target cells. Every projection of the high-dimensional rectangle into 2D space is again a rectangle, which is then used to derive a marker set as a gating strategy. Furthermore, we benchmarked ConvexGating against two supervised binary classification algorithms, namely a linear support vector machine (SVM) classifier and a radial basis function (RBF) kernel SVM^54^. The linear SVM directly searches for one separating hyperplane in M-dimensional marker space that optimally distinguishes target cells from non-target cells. The RBF SVM is based on a nonlinear rbf kernel transformation that maps the input data into a vector space with enhanced separability of targets and non-targets. However, the increased separability induced by a nonlinear mapping comes at the cost of interpretability. In contrast to Hypergate and ConvexGating, the output of the SVM classifiers does not directly translate into a gating strategy. Both SVM classifiers were implemented with the aid of the scikit-learn Python package v. 1.1.2^54^.

For executing hypergate, we made use of the most recent hypergate R package version 0.8.3 (https://github.com/ebecht/hypergate) in R v. 4.0.3. For all comparisons, we first created train and test splits of all datasets such that all methods were run on the exact same data. To enable sufficient coverage of granular target cell types, we constructed training datasets with a fixed 1:1 ratio of target to non-target cells, using the remaining data as the test set. The training data included 70% of the available target cells, with a maximum total of 50,000 cells. When 70% of the target cells were insufficient to reach the specified 1:1 ratio, fewer cells were used for training to preserve this balance.

The benchmark analysis was conducted for two different flow cytometry datasets (DC panel, large PBMC panel) with two annotation levels each (level 1 and level 2). Furthermore, the human bone marrow cyTOF dataset (ref. ^34^, see Data analysis), which consists of four annotation levels (level 2, level 3, level 4, and level 5), was included in the benchmark analysis. As a performance measure for this benchmark analysis, we referred to precision, recall and ${F}_{1}$ score (see performance evaluation).

### Comparison of heuristic and classifier-based marker selection

We compared marker rankings obtained from ConvexGating’s default heuristic marker-selection approach with marker rankings generated by alternative classifier-based methods using the human bone marrow cyTOF data set. For annotation levels 2, 3, and 4, we randomly subsampled 10,000 cells per sample. For each cell type within the respective annotation level and sample, we computed marker importance for distinguishing that cell type from all others using ConvexGating default heuristic selection method, a single decision tree classifier (DecisionTreeClassifier, scikit-learn v1.4.2), and a single SVM classifier (LinearSVC, scikit-learn v1.4.2). We then calculated pairwise Spearman rank correlations between the rankings and recorded computation time.

### Performance evaluation

The goodness of a gating strategy is evaluated based on recall, precision and ${F}_{1}$ score. Recall quantifies the portion of target cells (positives) identified by the gating strategy. Precision refers to the fraction of target cells inside the gated population. A high recall value indicates that most target cells are captured by the gating strategy, while a high precision value indicates purity in the gated population with target cells predominating over non-target cells (negatives).

4 $${{\mathrm{recall}}}\,=\frac{{{\mathrm{true}}}\,{{\mathrm{positives}}}}{{{\mathrm{true}}}\,{{\mathrm{positives}}}\,+\,{{\mathrm{false}}}\,{{\mathrm{negatives}}}}$$

5 $${{\mathrm{precision}}}=\frac{{{\mathrm{true}}}\,{{\mathrm{positives}}}}{{{\mathrm{true}}}\,{{\mathrm{positives}}}+{{\mathrm{false}}}\,{{\mathrm{positives}}}}$$

${F}_{1}$ is the harmonic mean of precision and recall. For a gating strategy, a high ${F}_{1}$ indicates both a high identification rate of target cells and purity in the gated population.

6 $${F}_{1}=\frac{2*{{\mathrm{recall}}}*{{\mathrm{precision}}}}{{{\mathrm{recall}}}+{{\mathrm{precision}}}}$$

### Contour-based density visualization

For visualization of the derived gating strategies, density plots were generated for each applicable hierarchy level using 2D histograms of target and non-target populations in the corresponding 2D marker space, followed by Gaussian smoothing to obtain continuous density estimates. Contour levels were adaptively selected based on population size, enabling clear visualization of target and non-target distributions in large flow-cytometry datasets.

### ConvexGating algorithm

Let ${X}^{{{\mathrm{data}}}}\epsilon \,{R}^{{{\mathrm{NxM}}}}$ be our data matrix where N denotes the number of cells and M denotes the number of markers. Optionally, we add a small amount of random noise from a uniform distribution over [-5${e}^{-5}$, 5${e}^{-5}$) per entry of ${X}^{{{\mathrm{data}}}}$ to ensure robust internal processes within our algorithm, particularly in cases where many cells exhibit zero expression for a specific feature. Cells are assumed to be labeled, i.e., assigned to one of C cell populations (clusters). Our model is capable of learning a gating strategy for extracting specific cell populations from the remaining cells in an interpretable way, mimicking the behavior of human experts performing manual gating. Cells of the desired population are referred to as targets. All other cells are referred to as non-targets.

#### Deriving gating strategies

We define T as the maximum number of hierarchies in the gating strategy, while F denotes the set of available markers. Per hierarchy, we have a target population ${P}^{t}$ and a non-target population ${P}^{{nt}}$. Finding an appropriate gate per hierarchy is a three-step procedure, which we describe in the following.

##### Step 1. Selecting a marker combination for 2D marker space

The goal of our heuristic marker selection approach is to identify those two markers along which the distribution of the target population ${P}^{t}$ and the distribution of the non-target population ${P}^{{nt}}$ differ the most. For ${P}^{t}$ and ${P}^{{nt}}$, we calculate the $1{st}$, the $50{th}$ and the $99{th}$ percentile along each marker fϵF. These statistical quantities are used as a heuristic for the distribution of ${P}^{t}$ and ${P}^{{nt}}$ along marker f. We select those two markers that promise the largest difference in distribution for ${P}^{t}$.

For each marker *f ϵF*, we define

7 $${{\mathbf{q}}}_{f}({P}^{t}): \! \!=\,{({p}_{1,f}{p}_{50,f}{p}_{99,f})}^{T}\,$$

8 $${{\mathbf{q}}}_{f}({P}^{{nt}}): \! \!={({\tilde p}_{1,f}{\tilde p}_{50,f}{\tilde p}_{99,f})}^{T}$$

where ${p}_{u,f}$ denotes the ${uth}$ percentile of ${P}^{t}$ along marker *f* and ${\tilde p}_{u,f}$ the ${uth}$ percentile of ${P}^{{nt}}$ along marker *f*. We then calculate for all ${f}\epsilon {F}$ the heuristic

9 $${d}_{f}: \! \!=\,\frac{1}{2}{MSE}({q}_{f}({P}^{t}),{q}_{f}({P}^{{nt}}))$$

where MSE denotes mean squared error. We select those two markers with the highest heuristic value ${d}_{{f}}$ for the corresponding hierarchy.

Optional alternative marker selection strategies are implemented, in which either a shallow decision tree (max depth = 4) or a class-balanced linear SVM is trained on each marker independently to separate the target from the non-target population. Markers are then ranked by the resulting F1 scores, and the top two markers are selected for the corresponding hierarchy.

##### Step 2. Learning gate location

After selection of markers ${m}_{1}$ and ${m}_{2}$, we learn the optimal location of gate G in 2D marker space by minimizing a problem-specific loss via stochastic gradient descent.

Gate specification

We define a gate in 2D marker space as the intersection of K halfspaces ${H}^{+}$ in 2D, a design that allows for flexible adaptation of gate boundaries in response to the location of the target and non-target population. A halfspace ${H}^{+}$ is determined by a 2D hyperplane *C* with normal vector $w\in {R}^{2}$ and bias b∈R which reads as

10 $$C=\{{{\mathbf{x}}}\in {R}^{2}|{{\mathbf{w}}}^{T}{{\mathbf{x}}}+b=0\}.$$

The corresponding halfspace ${H}^{+}$ takes the form

11 $${H}^{+}=\{{{\mathbf{x}}}\in {R}^{2}|{{\mathbf{w}}}^{T}{{\mathbf{x}}}+b\ge 0\}.$$

Our convex gate G is then specified as

12 $$G={\cap }_{k=1}^{K}\{{{\mathbf{x}}}\in {R}^{2}| {{\mathbf{w}}}_{k}^{T}{{\mathbf{x}}}+{b}_{k}\ge 0\}$$

where ${{\mathbf{w}}}_{k}\in {R}^{2}$ denotes the normal vector and ${b}_{k}\in R$ the bias of the ${kth}$ hyperplane.

Loss function

Our loss *L* quantifies how well gate G captures target cells and separates them from non-target cells, thereby assessing the suitability of G for gating. We apply a weighted version of binary cross-entropy loss. For marker expression input ${{\mathbf{x}}}\in \,{R}^{2}$ with label $y\in \{{{\mathrm{0,1}}}\}$ and weight parameter α, our loss ${L}_{\alpha }$ computes as follows. For all hyperplanes k=1,...,K we calculate

13 $$\,{u}_{k}={{\mathbf{w}}}_{k}^{T}{{\mathbf{x}}}+{b}_{k}$$

which puts the location of input ${{\mathbf{x}}}$ into relation with the ${kth}$ hyperplane. In case ${{\mathbf{x}}}\epsilon {G}$, we have for all ${k}=\,1,...,K$ that ${u}_{k}\ge 0.$ Next, we calculate the predicted label

14 $$\hat{y}={\prod }_{k=1}^{K}{\sigma }_{s}({u}_{k})$$

where ${\sigma }_{s}$ denotes the parameterized sigmoid which is defined as

15 $${\sigma }_{s}(z)=\frac{1}{1+{e}^{-{sz}}}$$

for an input ${z}\epsilon {R}$ and parameter ${s}\epsilon {R}$ (default: s=40). For sufficiently large ${s}\epsilon {R}$, the parameterized sigmoid ${\sigma }_{s}$ approximates the indicator function. Finally, our loss ${L}_{\alpha }$ reads as

16 $${L}_{\alpha }(x)=\alpha {ylog}(\hat{y})+(1-y)\log (1-\hat{y})$$

where α acts as a weight parameter adjusting the influence of target cells to the loss. Let ${n}_{{{\mathrm{target}}}} > 0$ denote the number of target cells and ${n}_{{{{\mathrm{non}}}}_{-}{{\mathrm{target}}}}$ the number of non-target cells.

We set

17 $$\alpha=\frac{{n}_{{{{\mathrm{non}}}}_{-}{{\mathrm{target}}}}}{{n}_{{{\mathrm{target}}}}}{1}_{\{{n}_{{{{\mathrm{non}}}}_{-}{{\mathrm{target}}}} > {n}_{{{\mathrm{target}}}}\}}+{1}_{\{{n}_{{{{\mathrm{non}}}}_{-}{{\mathrm{target}}}}\le {n}_{{{\mathrm{target}}}}\}}$$

where ${1}_{\{\}}$ denotes the indicator function.

Regularized loss

We add two problem-specific regularization terms ${P}_{1}$ and ${P}_{2}$ to the loss ${L}_{\alpha }$ that encourage tight gates around the target population. The regularized loss is referred to as ${L}_{\alpha }^{{{\mathrm{reg}}}}$ (see Supplementary Note 3 for details):

18 $${L}_{\alpha }^{{{\mathrm{reg}}}}(x)={L}_{\alpha }(x)+{\lambda }_{1}{P}_{1}+{\lambda }_{2}{P}_{2}(x).$$

The weight parameters ${\lambda }_{1}$ and ${\lambda }_{2}$ control how much each regularization term affects the loss. Intuitively, P1 penalizes distance from the centroid of the target population, encouraging hyperplanes to stay aligned with the overall center of mass. P2 encourages the hyperplanes near the bulk of the target data, offering robustness against outliers. While these penalties often agree, they capture slightly complementary aspects, such that using both together consistently performed better than using no regularization or only one of the penalty terms.

Loss minimization

We obtain the optimal gate parameters ${{\mathbf{w}}}_{1},...,{{\mathbf{w}}}_{K}$ and ${b}_{1},...,{b}_{K}$ via stochastic gradient descent. We start by a random parameter initialization. Then, we repeatedly take a subset $S\subseteq {P}^{t}\cup {P}^{{nt}}\,$, compute ${L}_{\alpha }^{{reg}}(S)$ and backpropagate the loss to the gate parameters. We then update for k=1,...,K the normal vectors and biases based on the derivatives

19 $$\frac{d{L}_{\alpha }^{{reg}}(S)}{d{{\mathbf{w}}}_{k}}$$

20 $$\frac{d{L}_{\alpha }^{{{\mathrm{reg}}}}(S)}{d{b}_{k}}.$$

To control for the randomness induced by random parameter initialization, we fix four (default) normal vectors aligned with the principal components of the target population ${P}^{t}$. The rationale behind this approach is that the principal components of ${P}^{t}$ reflect the axes of greatest variation within the target population, thereby offering a more targeted and data-driven starting point compared to completely random initialization of all hyperplanes that will eventually form the gate. This strategy is implemented to reduce the risk of non-convergence due to suboptimal random initialization.

Adaptive grid search

Finding an optimal gate G requires a careful selection of the weight parameters ${\lambda }_{1}$ and ${\lambda }_{2}$ that control the influence of the penalty terms ${P}_{1}$ and ${P}_{2}$ in the regularized loss ${L}_{\alpha }^{{{\mathrm{reg}}}}$ (see Supplementary Note 3). We perform an adaptive grid search to find those weight parameters ${\lambda }_{1}$ and ${\lambda }_{2}$ that lead to the best gate G with respect to separation of target cells and non-target cells (performance measure: ${F}_{1}$ (default) or recall). To reduce the computational complexity, we assign equal weight to both penalty terms ${P}_{1}$ and ${P}_{2}$, thus setting ${\lambda }_{1}$ = ${\lambda }_{2}$.

To determine ${\lambda }_{1}$,$\,{\lambda }_{2}$, we define an initial range (default: [0, 2, 4, 8, 10]) of possible weight parameters that is iteratively updated based on the two best-performing weight parameters ${h}_{1}$ and ${h}_{2}$ in the current range of values. Let ${{grid}}_{{divisor}} > 0$ and define

21 $$\Delta h\,: \! \!=|\frac{{h}_{1}-\,{h}_{2}}{{{{\mathrm{grid}}}}_{{{\mathrm{divisor}}}}}|.$$

The updated search range then starts either at $\min \{{h}_{1},{h}_{2}\}-\varDelta {h}$ or at $\min \{{h}_{1},{h}_{2}\}\,+\varDelta {h}$ and extends until $\max ({h}_{1},{h}_{2})+\varDelta {h}$ with uniform steps of size Δh in-between. The number of search range updates can be chosen manually (default: 2). Eventually, the gate leading to best performance during the adaptive grid search is chosen as gate G.

##### Step 3. Gate optimization via convex hull

Let ${P}_{G}^{t}\subseteq {P}^{t}$ denote the subset of the target population that contains all target cells inside gate G after step 2. As final gate ${G}^{{{\mathrm{final}}}}$ we take the convex hull of ${P}_{G}^{t}$, that is

22 $${G}^{{final}}={convex}\,{hull}({P}_{G}^{t}).$$

${G}^{{{\mathrm{final}}}}$ encompasses the same target cells as G, but is only as large as necessary while still being convex. Non-targets contained in G drop out if they lie outside the convex hull of ${P}_{G}^{t}$.

#### Evaluation

The goodness of a gating strategy is evaluated based on recall, precision and ${F}_{1}\,$(see section Performance evaluation). Our model is encouraged to optimize ${F}_{1}$ with an emphasis on precision (default) as we aim to construct gates with high cell type purity, such that our gating strategies can be applied for cell sorting and subsequent functional testing of the cells.

##### Statistics and reproducibility

Boxplots are visualized as boxes representing 25th percentile, median and 75th percentile, respectively, and whiskers showing 1.5 * interquartile range. For visualization in the volcano plots, a threshold of *p* < 0.05 and a Log_2_ fold change >0.5 were applied to define differentially expressed genes. Data were filtered for quality control as described in the respective data analysis section. The investigators were not blinded to allocation during experiments and outcome assessment, no statistical method was used to predetermine sample size, and the experiments were not randomized as the experiments were performed to validate the ConvexGating algorithm. Original files, data and codes are publicly available to reproduce data reported in this manuscript.

### Reporting summary

Further information on research design is available in the Nature Portfolio Reporting Summary linked to this article.

## Supplementary information

Supplementary Information

Reporting Summary

Transparent Peer Review file

## Source data

Source Data Excel File

Source Data Figure 3f

Source Data Figure 6i

## Data Availability

CyTOF data of human bone marrow (Oetjen et al.) were obtained from FlowRepository.org (accession codes: “FR-FCM-ZYQ9 [http://flowrepository.org/id/FR-FCM-ZYQ9]”, “FR-FCM-ZYQB [http://flowrepository.org/id/FR-FCM-ZYQB]”). CyTOF data set of T cells of COVID-19 patients was downloaded from record number “5771937 [https://zenodo.org/record/5771937/files/data_Tcells_annotated.csv.gz]”. We obtained the compensated flow cytometry data set of T cells of COVID-19 patients (Georg et al., “2021.12.040 [https://doi.org/10.1016/j.cell.2021.12.040]”) and were shared courtesy of the authors. CITE-seq data set of the immune cell compartment of COVID-19 patients and healthy donors was downloaded from the “COVID-19 Cell Atlas [https://www.covid19cellatlas.org/]”. We obtained the compensated flow cytometry data set of PBMCs samples from five healthy human donors measured on a BD Symphony A5 instrument, encompassing 27 markers, which was previously published by Knoll et al. (“fimmu.2023.1275136, [https://doi.org/10.3389/fimmu.2023.1275136]”) and was shared courtesy of the authors. The flow cytometry measurements of whole blood using six markers to identify DCs were obtained as an in-house dataset. The informed consent obtained from donors did not cover public release of individual-level raw data. The processed data required to interpret, verify, and extend the reported findings—including gating strategy, compensation matrix, summary statistics per marker/population, and representative plots—are provided as supplementary material. The raw and processed FLASH-seq data of human CD8 T cells generated in this study have been deposited in the European Genome-phenome Archive (EGA) under accession code “EGAS50000001005 [https://ega-archive.org/studies/EGAS50000001005]”. The data are available under restricted access due to data protection requirements under the General Data Protection Regulation (GDPR). Access can be obtained by submitting a data access request through the EGA portal using the accession code. Requests are reviewed by the study’s Data Access Committee (DAC), which evaluates eligibility in accordance with the applicable ethical and legal requirements. Decisions on access requests are typically made within a few days. Approved users will be granted access for the duration of the approved research project. The raw and processed FLASH-seq data of mouse WAT cells generated in this are available on ArrayExpress under accession number “E-MTAB-1730 [https://doi.org/10.6019/E-MTAB-17301]”. Any additional requests for information can be directed to, and will be fulfilled by, the corresponding author. Source data are provided with this paper.

## Code availability

ConvexGating is implemented in Python, available on PyPI and on “GitHub [https://github.com/buettnerlab/convexgating]” and on “Code Ocean [https://codeocean.com/capsule/3807581/tree]”. Documentation and tutorials can be found on https://convexgating.readthedocs.io/en/latest/index.html. All scripts and notebooks used to create the figures are available on https://github.com/buettnerlab/reproducibility_convex_gating.
