# Supplementary material for: ConvexGating infers gating strategies from clusters in single cell cytometry data
Source: Nat Commun. 2026 Sep 18;17:9975. doi: 10.1038/s41467-026-77360-z (PMC13588814; doi:10.1038/s41467-026-77360-z)
Supplement: Supplementary file 1 [file 41467_2026_77360_MOESM1_ESM.pdf]

Supplementary Figure 1

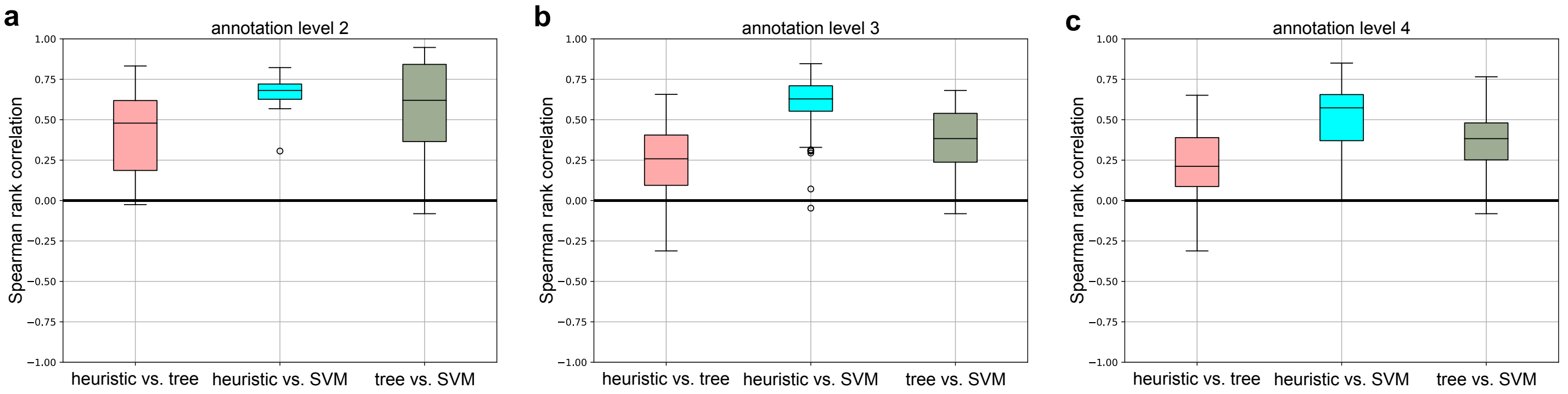

Supplementary Figure 1. Comparison of heuristic marker selection with feature importance from classifiers. Boxplots showing Spearman rank correlations between the rankings of marker importance derived from the heuristic marker selection, a tree-based classifier, and an SVM classifier. Results are shown for all cell types across all samples in the human bone marrow cyTOF data set at annotation level 2 (a), annotation level 3 (b), and annotation level 4 (c).

Supplementary Figure 2

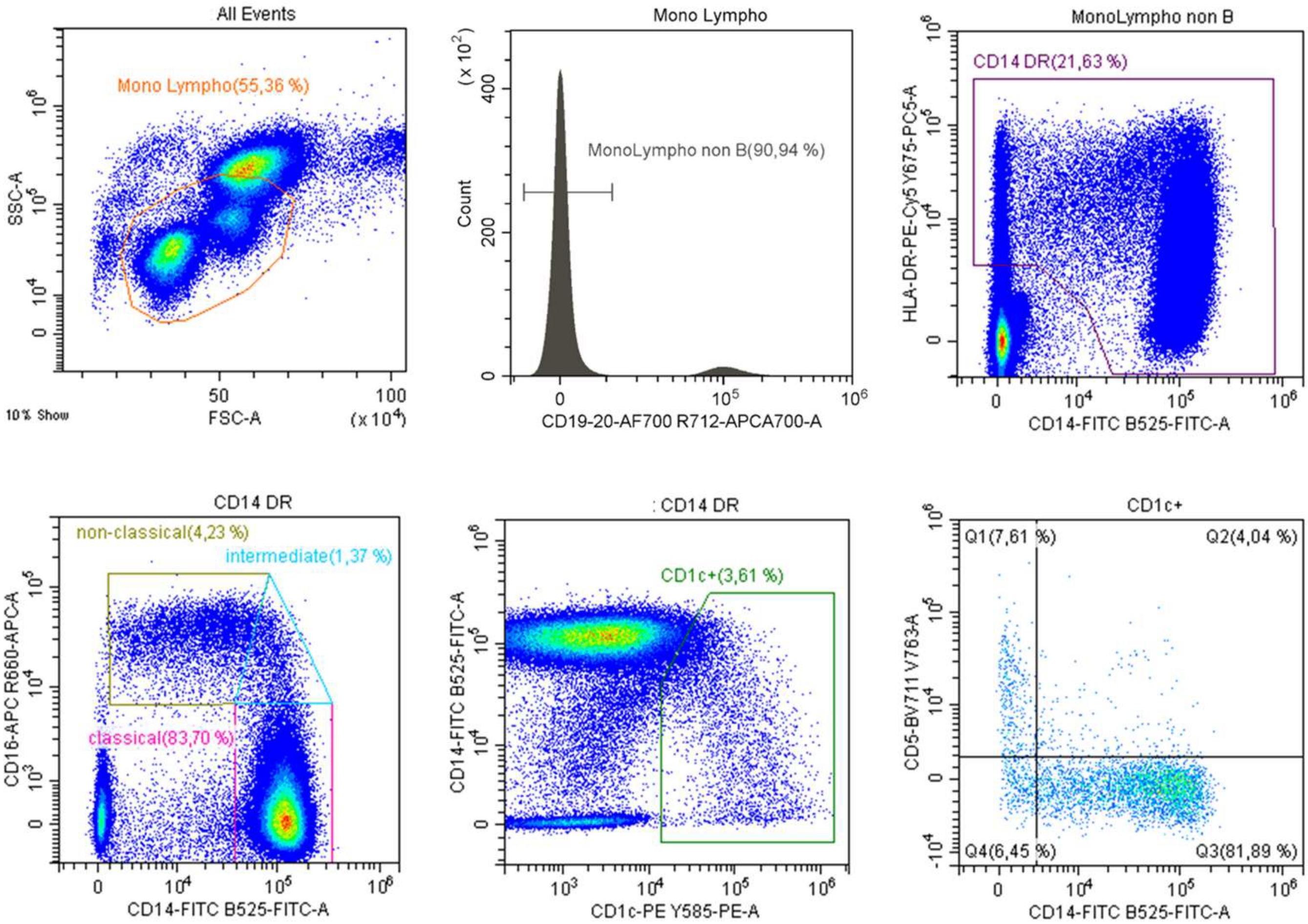

Supplementary Figure 2. Overview of the manual gating strategy on the DC panel. We remove debris (top left panel) and identify B cells (CD19/20+) (top middle panel), monocytes and DC2s (HLA-DR+ or CD14+) (top right panel). We then identify monocyte subtypes (based on CD14 and CD16) (bottom left panel), separate DC2 from monocytes (based CD14 and CD1c) (bottom middle panel), and identify DC2 subtypes (CD1c+, based on CD5 and CD14) (bottom right panel). The gating schema is exemplified for donor ID KW.

Supplementary Figure 3

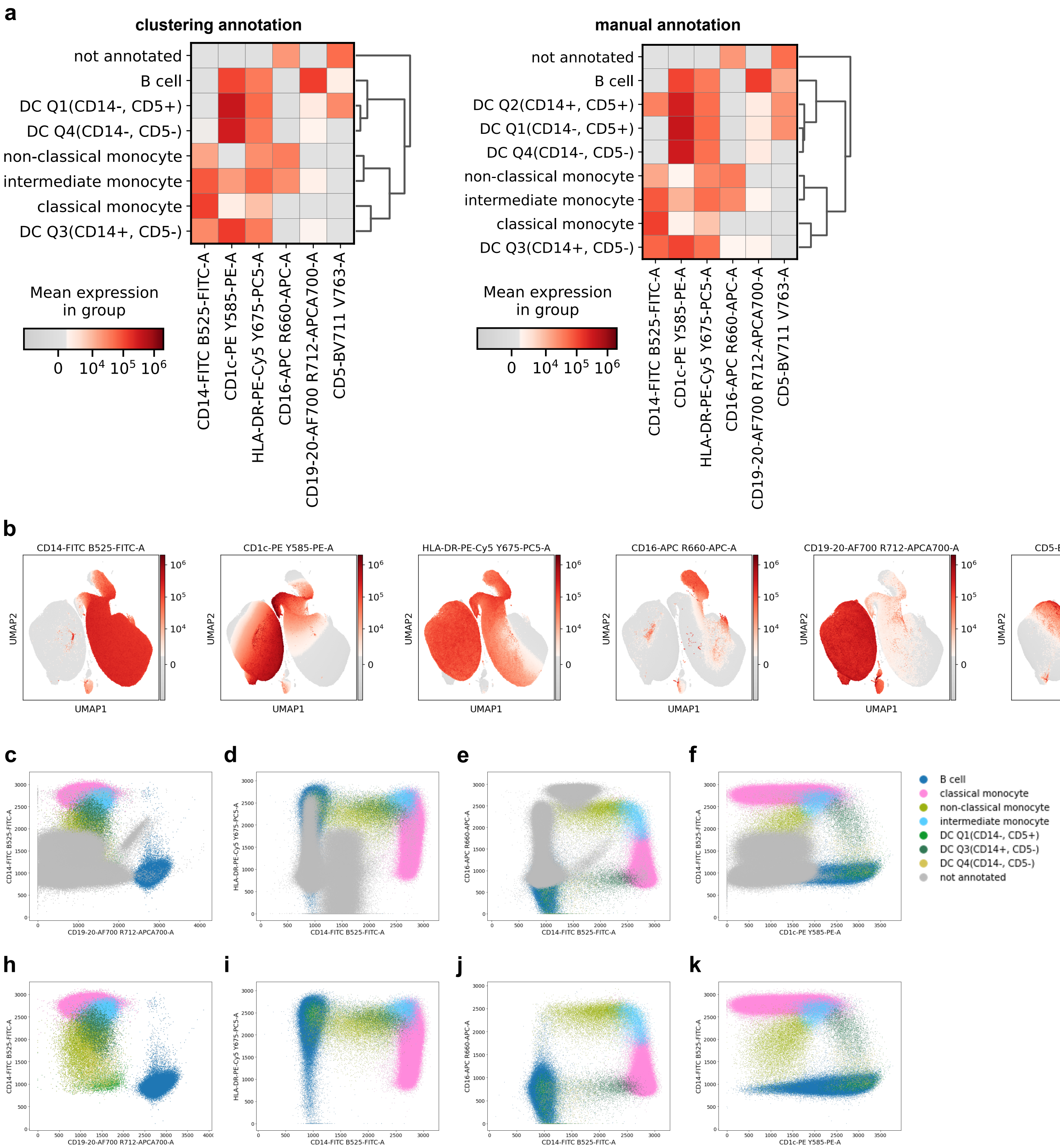

Supplementary Figure 3. Comparison of analysis manual and cluster-based analysis of the DC panel. (a) Mean marker levels for cell types identified through clustering (left panel) and manual annotation (right panel). (b) Marker level distribution for each cell displayed as UMAPs. (c-k) 2D scatter plots of the Leiden clustering-based cell type annotation using the DC panel for whole blood staining of three donors. (c-f) All cells colored by annotation level 2 including not annotated cells. (h-k) All annotated cells colored by annotation level 2 excluding not annotated cells.

Supplementary Figure 4

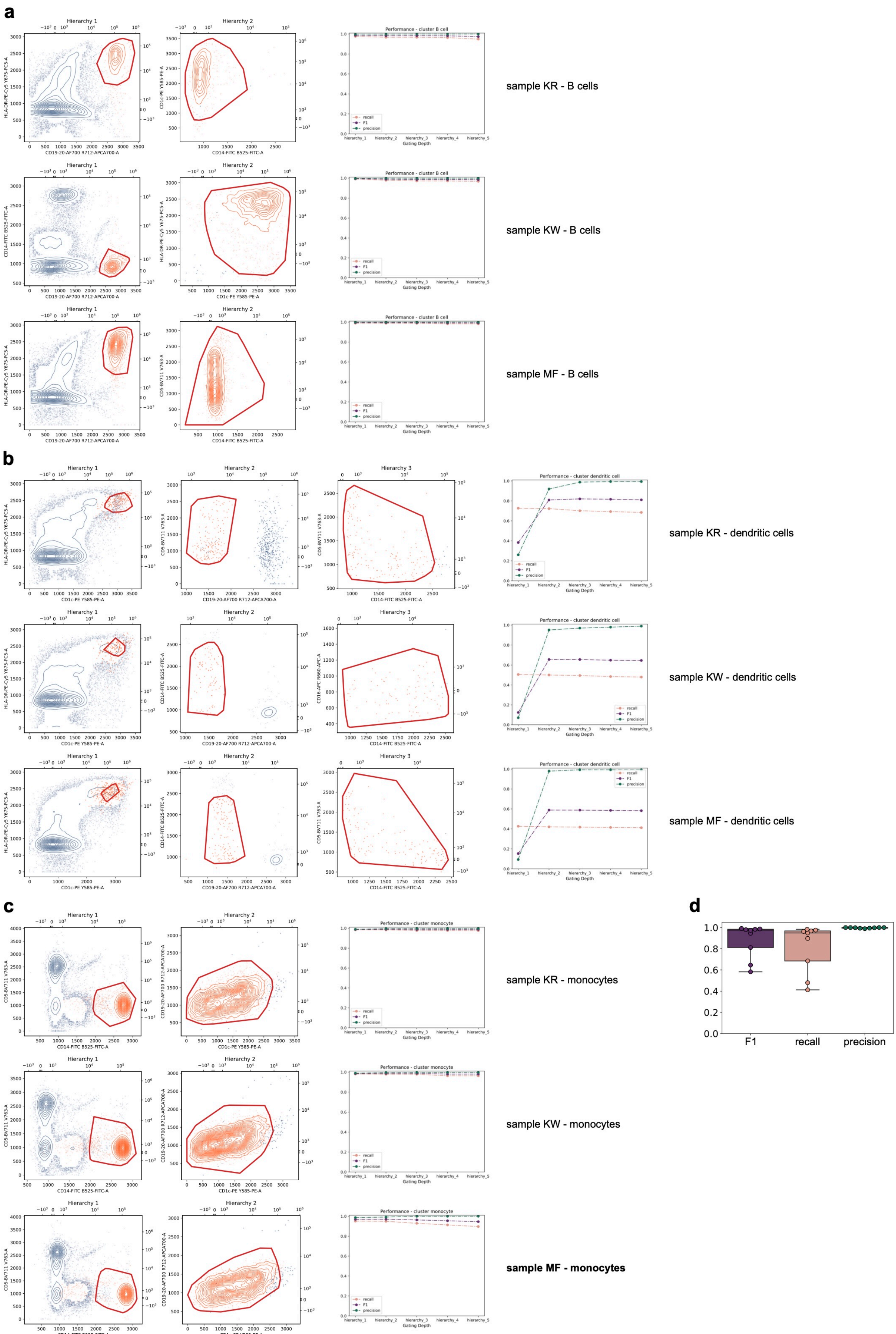

e

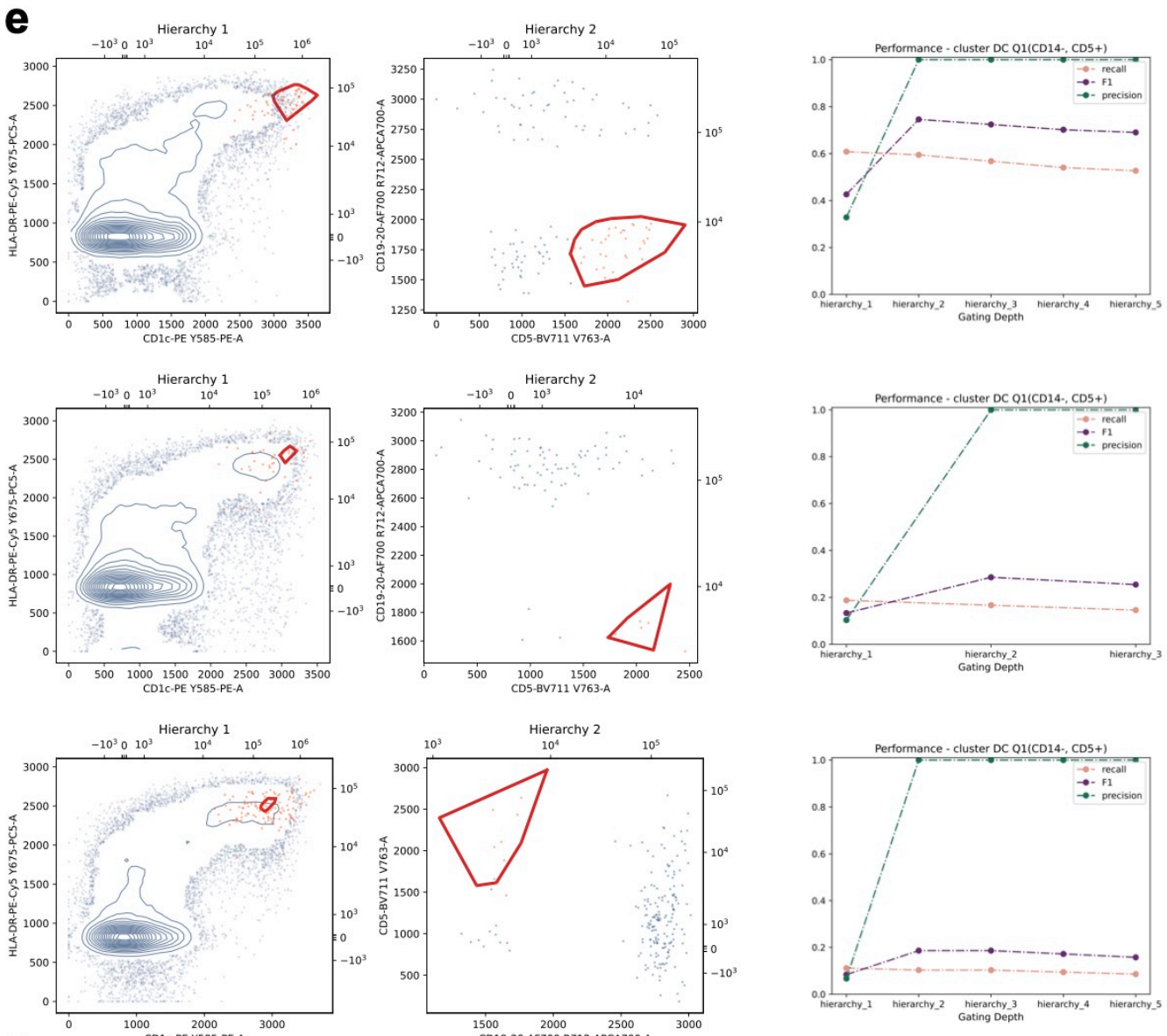

sample KR - dendritic cell Q1 (CD14-, CD5+)

sample KW - dendritic cell Q1 (CD14-, CD5+)

sample MF - dendritic cell Q1 (CD14-, CD5+)

f

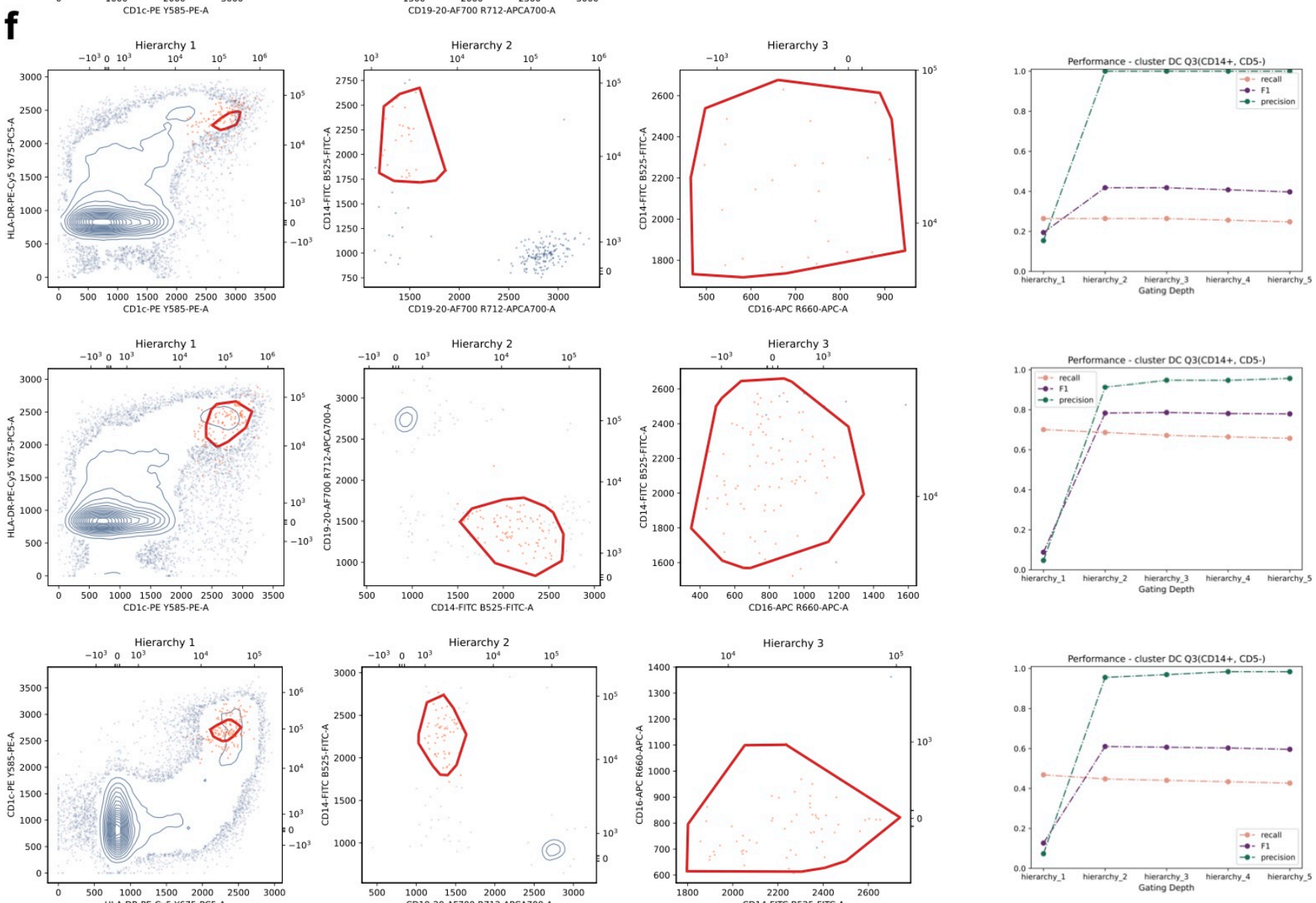

sample KR - dendritic cells Q3 (CD14+, CD5-)

sample KW - dendritic cells Q3 (CD14+, CD5-)

sample MF - dendritic cells Q3 (CD14+, CD5-)

g

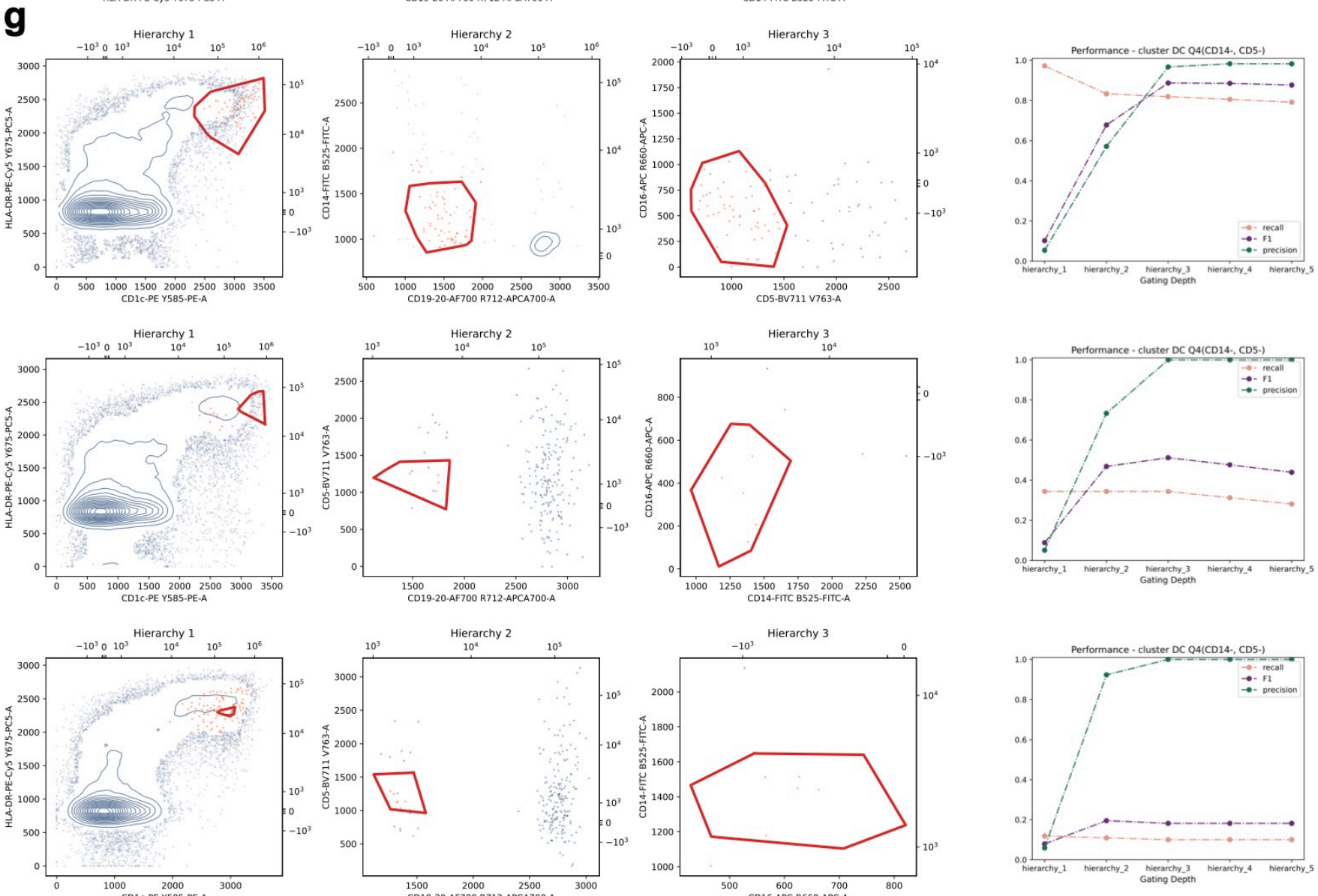

sample KR - dendritic cells Q4 (CD14-, CD5-)

sample KW - dendritic cells Q4 (CD14-, CD5-)

sample MF - dendritic cells Q4 (CD14-, CD5-)

h

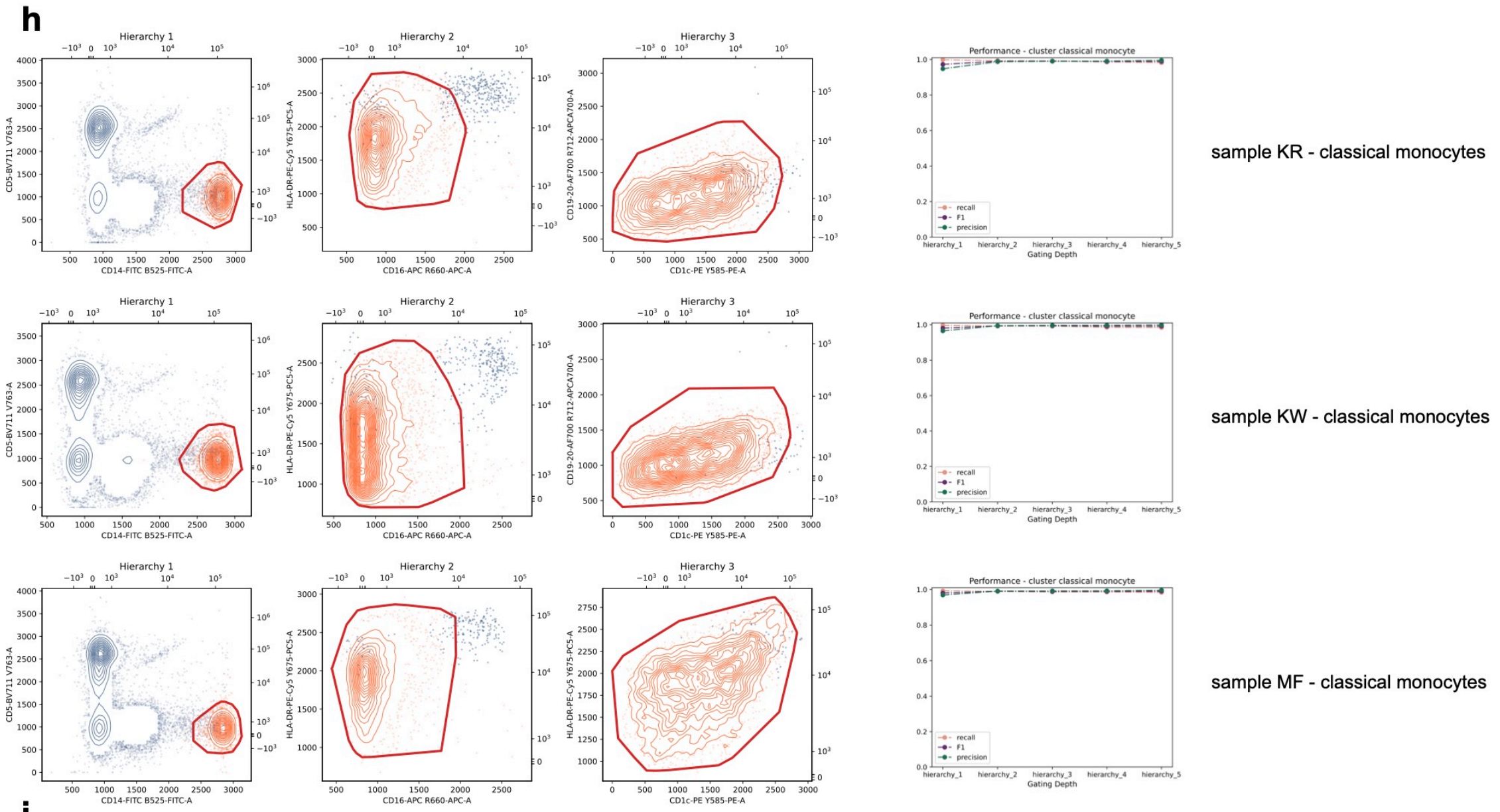

Hierarchy 1
Hierarchy 2
Hierarchy 3
Performance - cluster classical monocyte
sample KR - classical monocytes
sample KW - classical monocytes
sample MF - classical monocytes

i

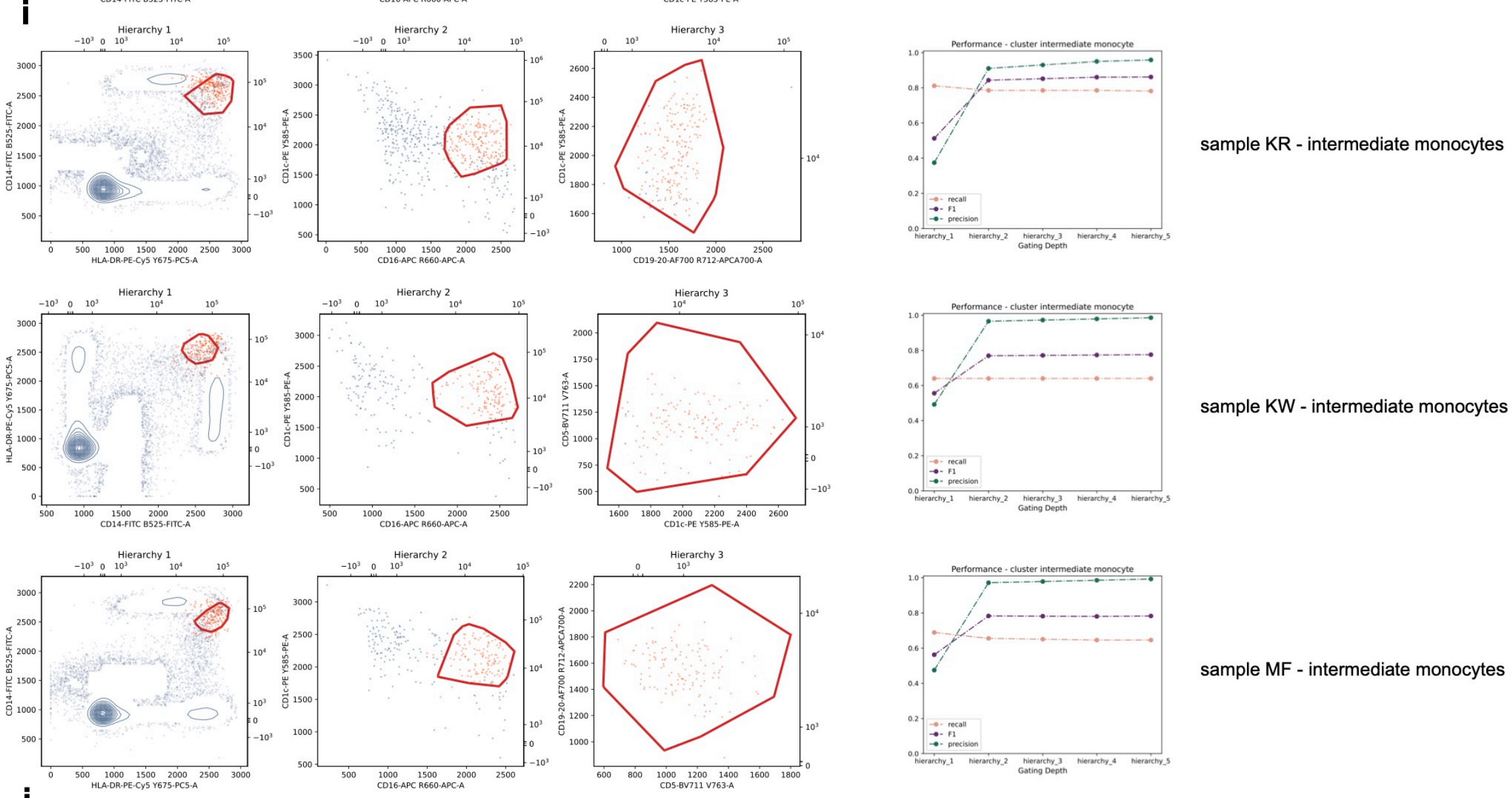

Hierarchy 1
Hierarchy 2
Hierarchy 3
Performance - cluster intermediate monocyte
sample KR - intermediate monocytes
sample KW - intermediate monocytes
sample MF - intermediate monocytes

j

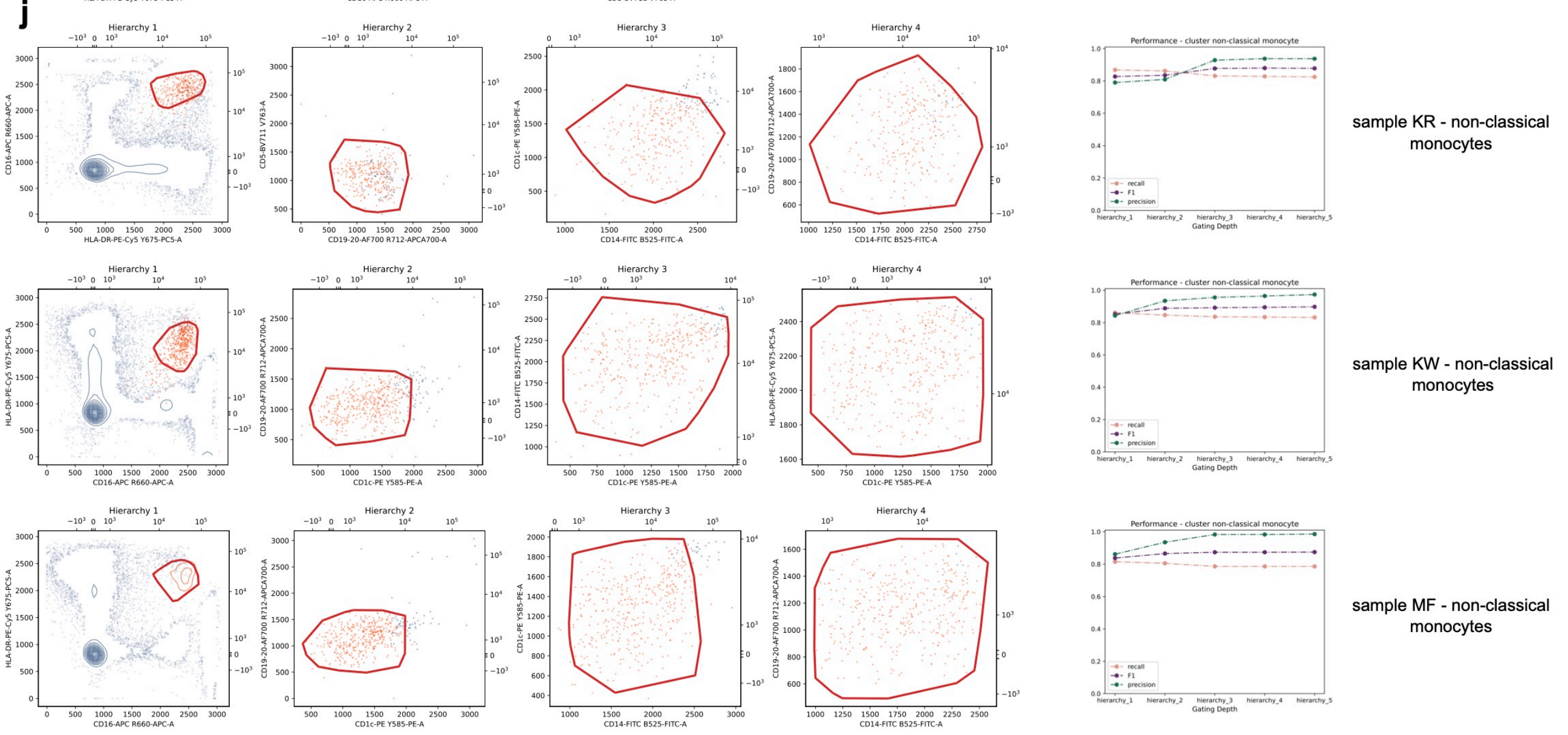

Hierarchy 1
Hierarchy 2
Hierarchy 3
Hierarchy 4
Performance - cluster non-classical monocyte
sample KR - non-classical monocytes
sample KW - non-classical monocytes
sample MF - non-classical monocytes

Supplementary Figure 4. Inferred gating strategies for the DC panel. (a-c) Gating strategies inferred by ConvexGating for cell type annotation level 1. (d) Boxplot showing precision, recall and F1 for all samples in cell type annotation level 1. (e-j) Gating strategies inferred by ConvexGating for cell type annotation level 2.

Supplementary Figure 5

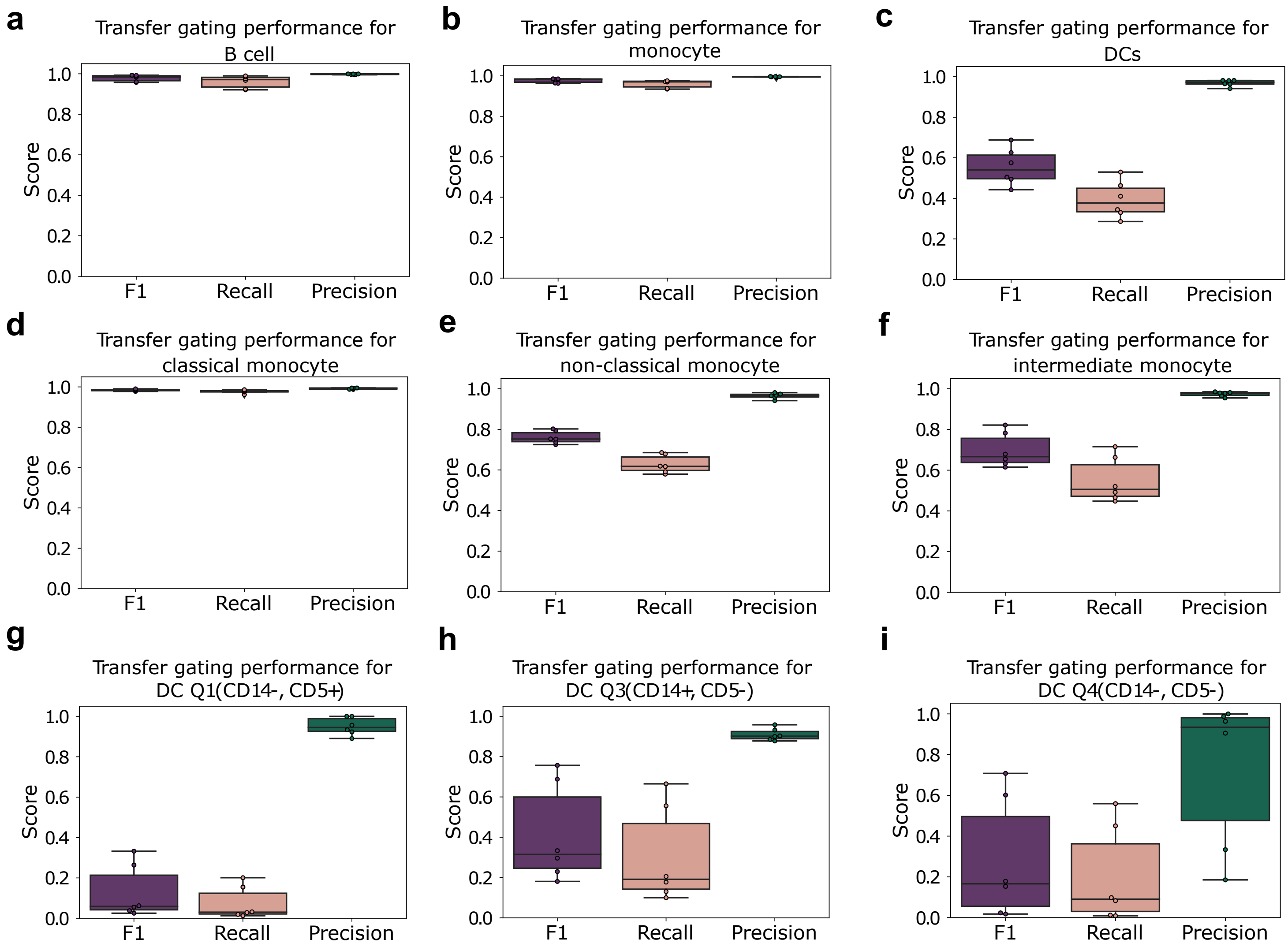

Supplementary Figure 5. Performance of gate transfer for all cell types in the small DC panel. Performance of gate transfer to the two other samples in the small DC panel for all cell types and subsets. Performance metrics for broad cell type annotation of B cells (a), monocytes (b) and DCs (c). Performance metrics subset annotation (cell type level 2) of classical monocytes (d), non-classical monocytes (e), intermediate monocytes (f), DC Q1 (CD14-, CD5+) (g), DC Q3 (CD14+, CD5-) (h), and DC Q4 (CD14-, CD5-) (i).

Supplementary Figure 6

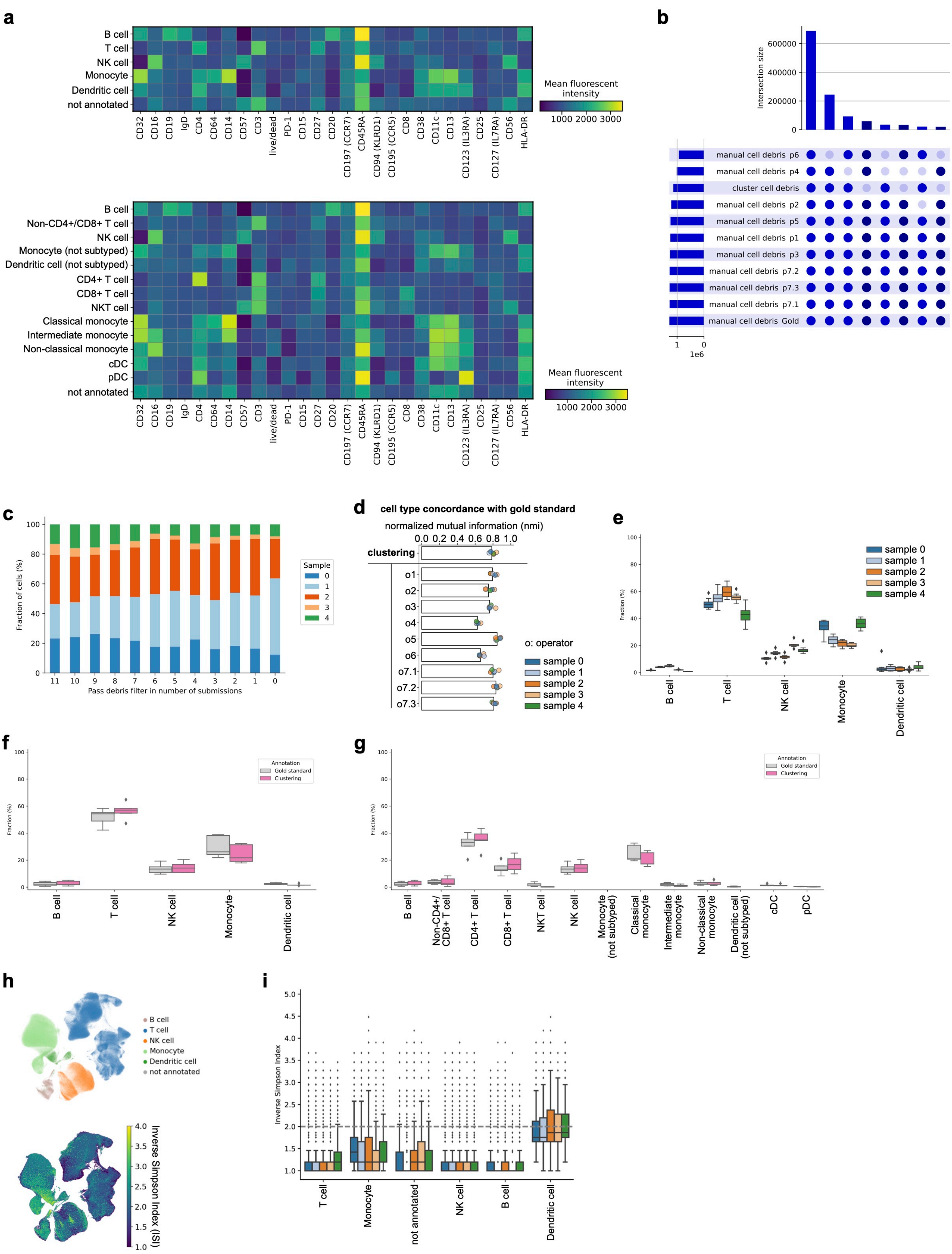

j

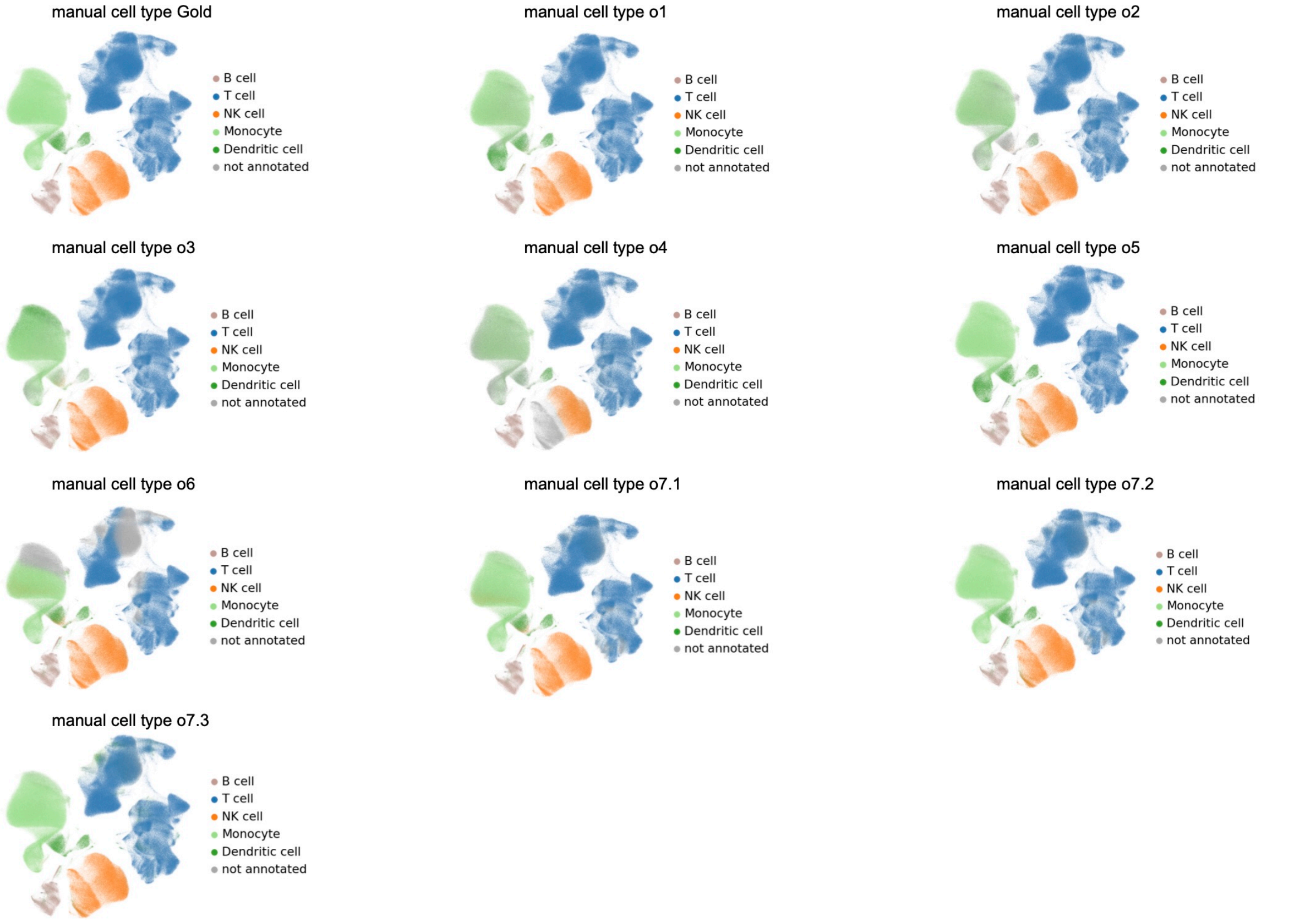
manual cell type Gold
manual cell type o1
manual cell type o2
manual cell type o3
manual cell type o4
manual cell type o5
manual cell type o6
manual cell type o7.1
manual cell type o7.2
manual cell type o7.3
B cell
T cell
NK cell
Monocyte
Dendritic cell
not annotated

k

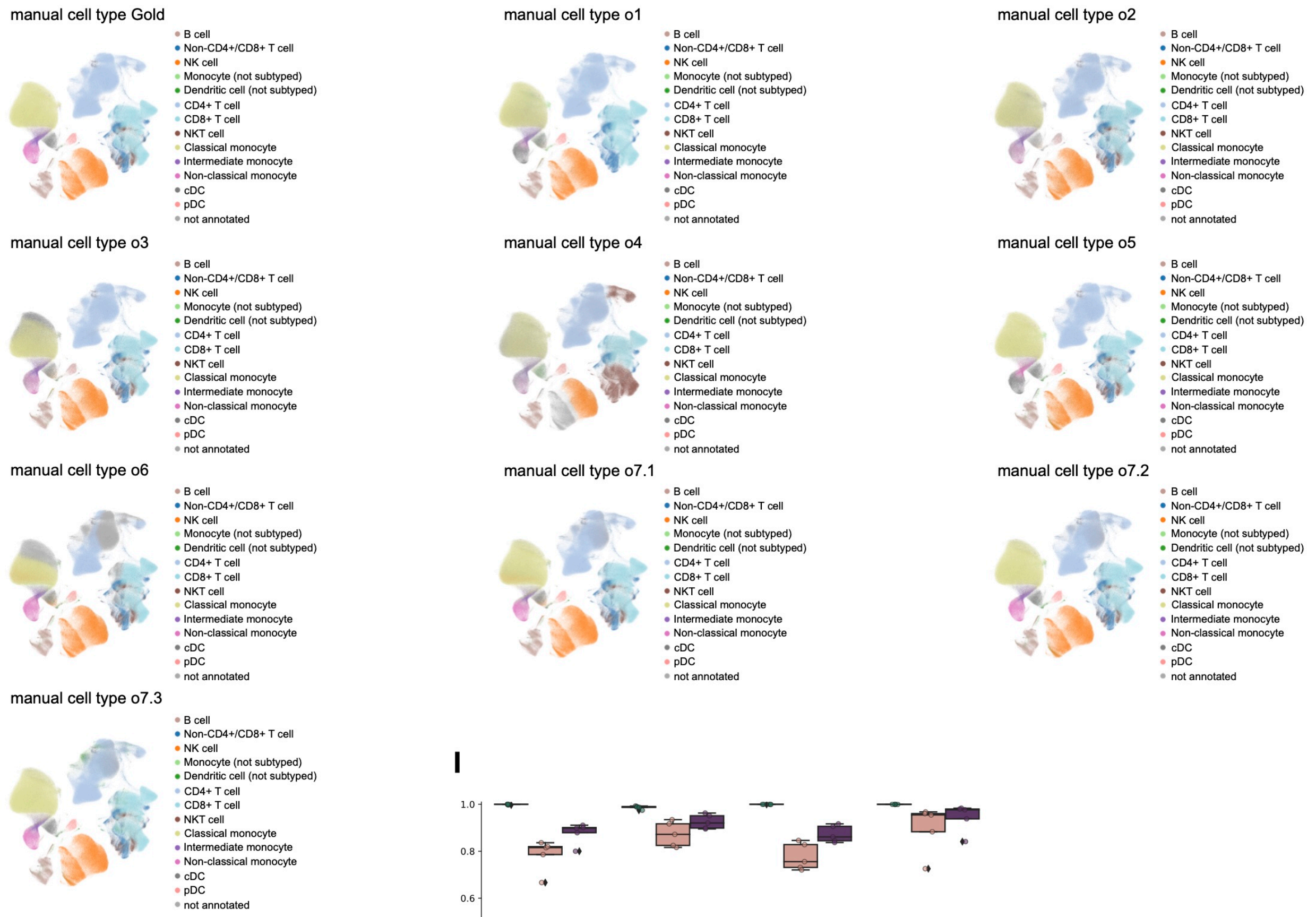
manual cell type Gold
manual cell type o1
manual cell type o2
manual cell type o3
manual cell type o4
manual cell type o5
manual cell type o6
manual cell type o7.1
manual cell type o7.2
manual cell type o7.3
B cell
Non-CD4+/CD8+ T cell
NK cell
Monocyte (not subtyped)
Dendritic cell (not subtyped)
CD4+ T cell
CD8+ T cell
NKT cell
Classical monocyte
Intermediate monocyte
Non-classical monocyte
cDC
pDC
not annotated

l

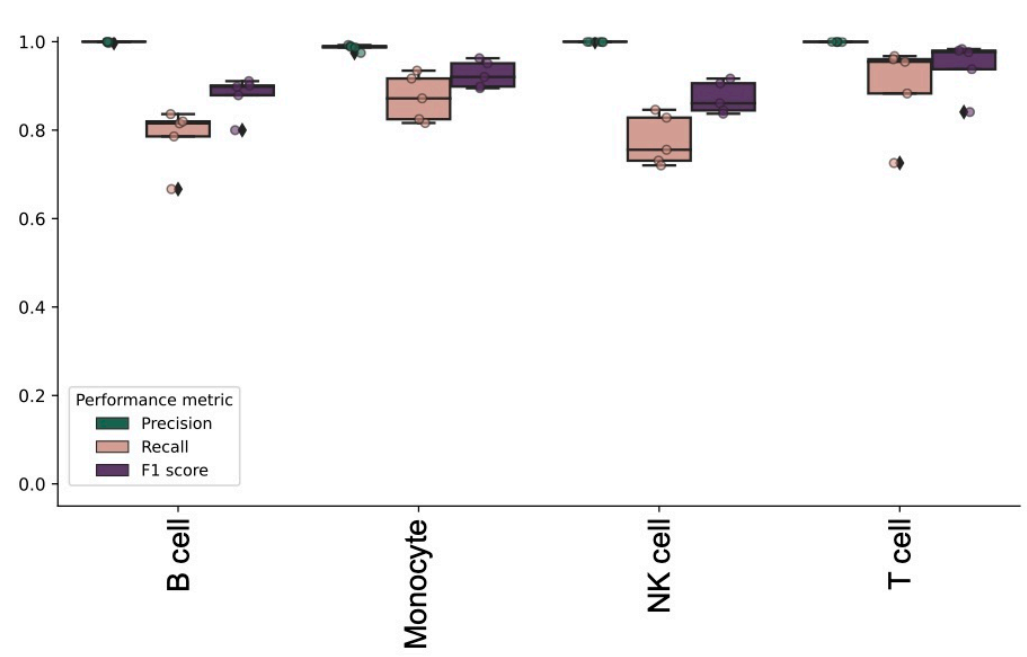
1.0
0.8
0.6
0.4
0.2
0.0
Performance metric
Precision
Recall
F1 score
B cell
Monocyte
NK cell
T cell

m

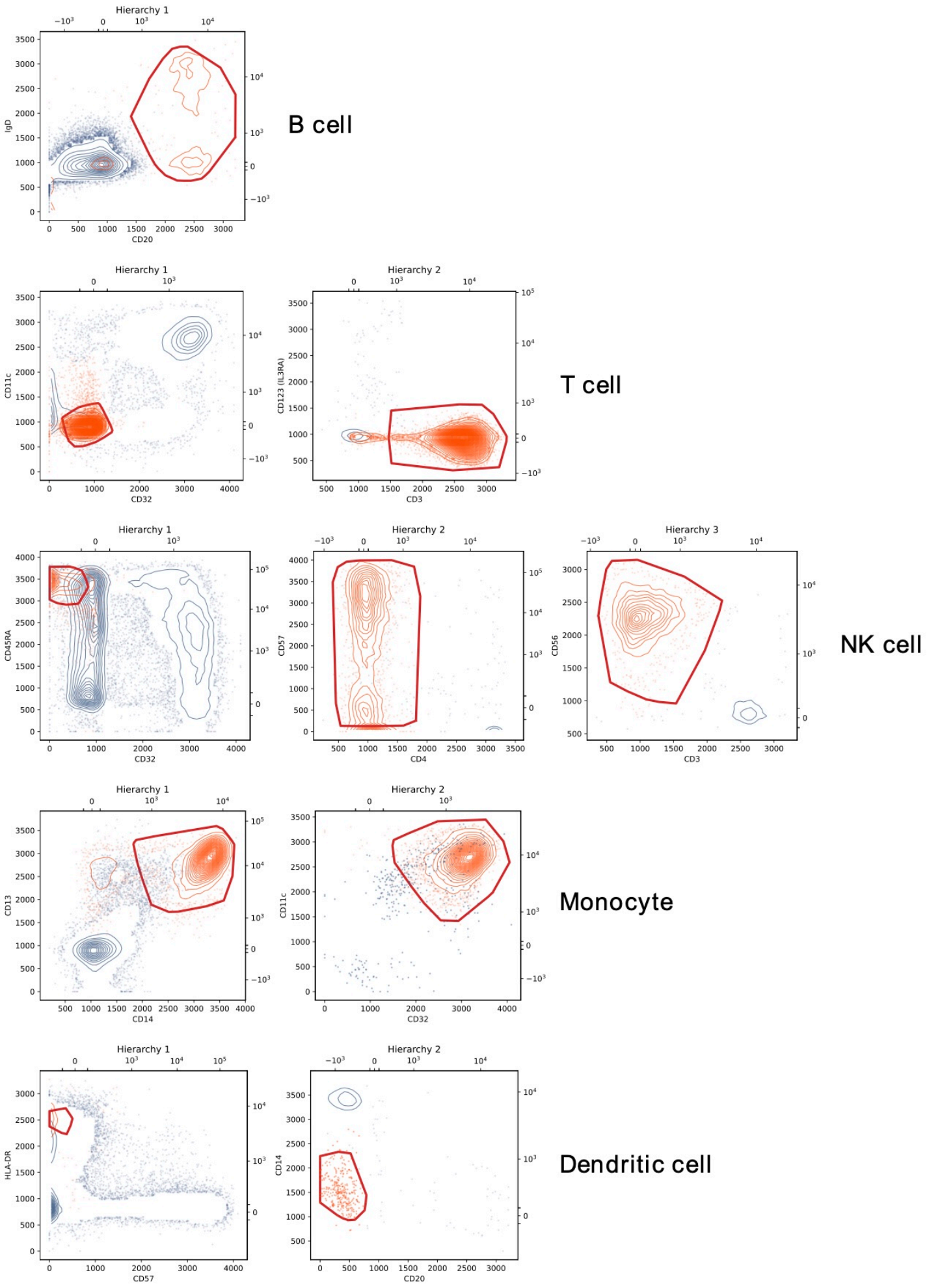

Hierarchy 1
IgD
CD20
B cell
Hierarchy 1
CD11c
CD32
Hierarchy 2
CD123 (IL3RA)
CD3
T cell
Hierarchy 1
CD45RA
CD32
Hierarchy 2
CD57
CD4
Hierarchy 3
CD56
CD3
NK cell
Hierarchy 1
CD13
CD14
Hierarchy 2
CD11c
CD32
Monocyte
Hierarchy 1
HLA-DR
CD57
Hierarchy 2
CD14
CD20
Dendritic cell

n

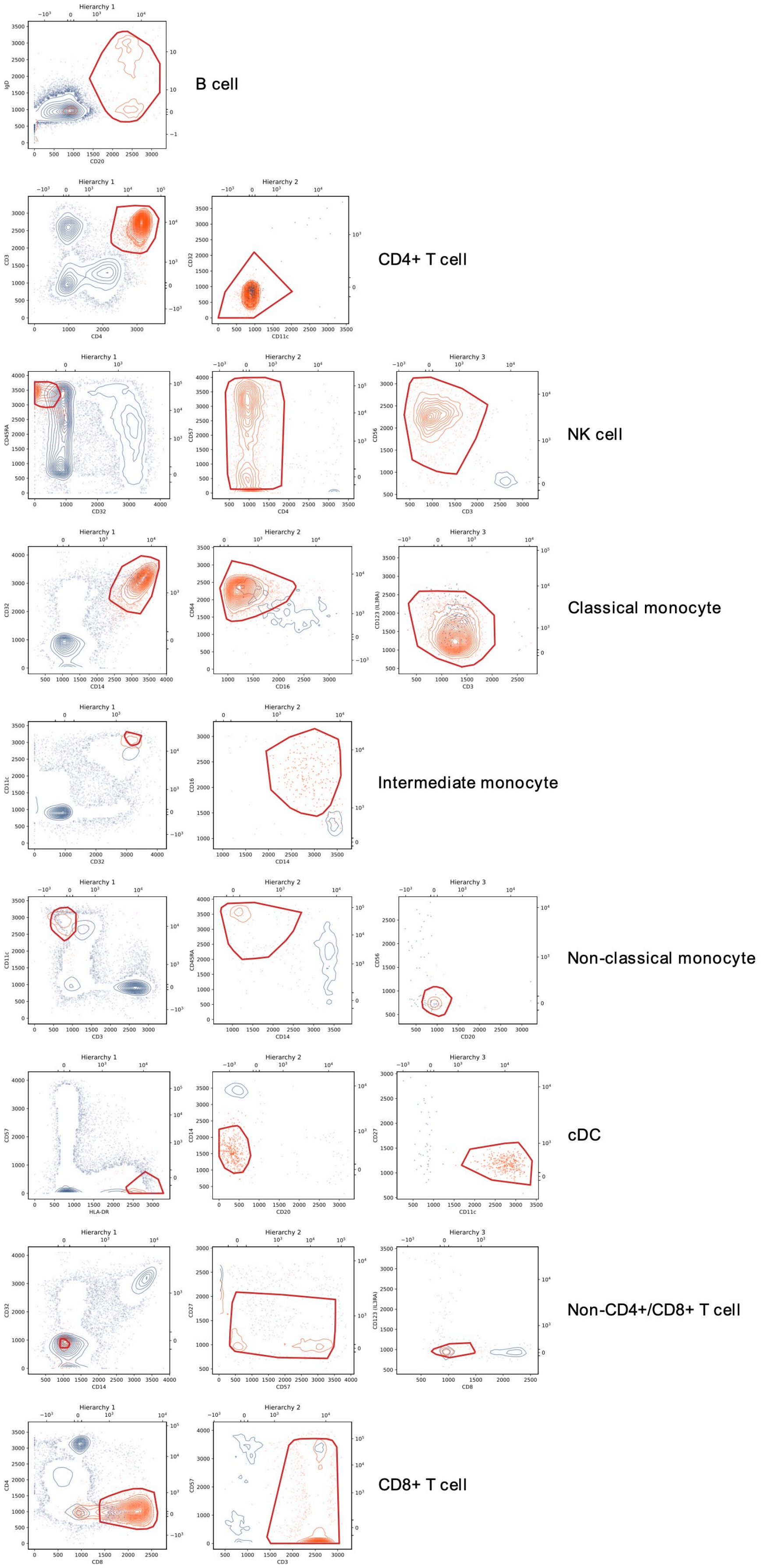

Hierarchy 1
Hierarchy 2
Hierarchy 3
B cell
CD4+ T cell
NK cell
Classical monocyte
Intermediate monocyte
Non-classical monocyte
cDC
Non-CD4+/CD8+ T cell
CD8+ T cell

Supplementary Figure 6. Detailed variability of human expert labeled data. (a) Mean fluorescent intensity of all 27 markers per consensus cell type (upper panel: coarse cell type level 1, lower panel: finer cell type level 2). (b) Upsetplot of the overlap of cells passing the QC filter (“valid cells”) across all submissions. (c) Fraction of valid cells per sample aggregated over all submissions. Cells consistently passing QC have highest scores. We do not observe a sample bias in the QC step. (d) Barplot of overlap of cell type labels for cell type level 1 compared to Gold standard measured by normalized mutual information (NMI). Dots colored by sample indicate NMI per sample. (e) Boxplot of cell type composition (cell type level 1) over all submissions(n=11, boxes represent 25th percentile, median and 75th percentile, respectively, whiskers show 1.5 * interquartile range and black diamonds represent outliers), colored by sample. (f)-(g) Boxplots of cell type composition (boxes represent 25th percentile, median and 75th percentile, respectively, whiskers show 1.5 * interquartile range and black diamonds represent outliers), comparing Gold standard with manual annotation for cell type level 1 (f) and cell type level 2 (g). (h) UMAPs displaying the consensus cell type labels (cell type level 1) over all submissions and Gold standard (upperpanel) and the number of effective labels per cell over all human expert labels measured by Inverse Simpson Index (ISI) (lower panel). (i) Boxplot of number of effective labels per cell over all submissions (n=11 submissions, boxes represent 25th percentile, median and 75th percentile, respectively, whiskers show 1.5 * interquartile range and black diamonds represent outliers), colored by sample, grouped by the consensus label over all submissions and the Gold standard. The grey dashed horizontal line indicates the effective number of 2 cell type labels. (j) UMAPs of all expert label submissions, the Gold standard and clustering for cell type level 1. (k) UMAPs of all expert label submissions, the Gold standard and clustering for cell type level 2. (l) Boxplots summarizing precision, recall and F1 scores per sample obtained by running ConvexGating on all samples for cell populations annotated by clustering on cell type level 1. (m) Gating strategies inferred by ConvexGating for cell type level 1. (n) Gating strategies inferred by ConvexGating for cell type level 2.

Supplementary Figure 7

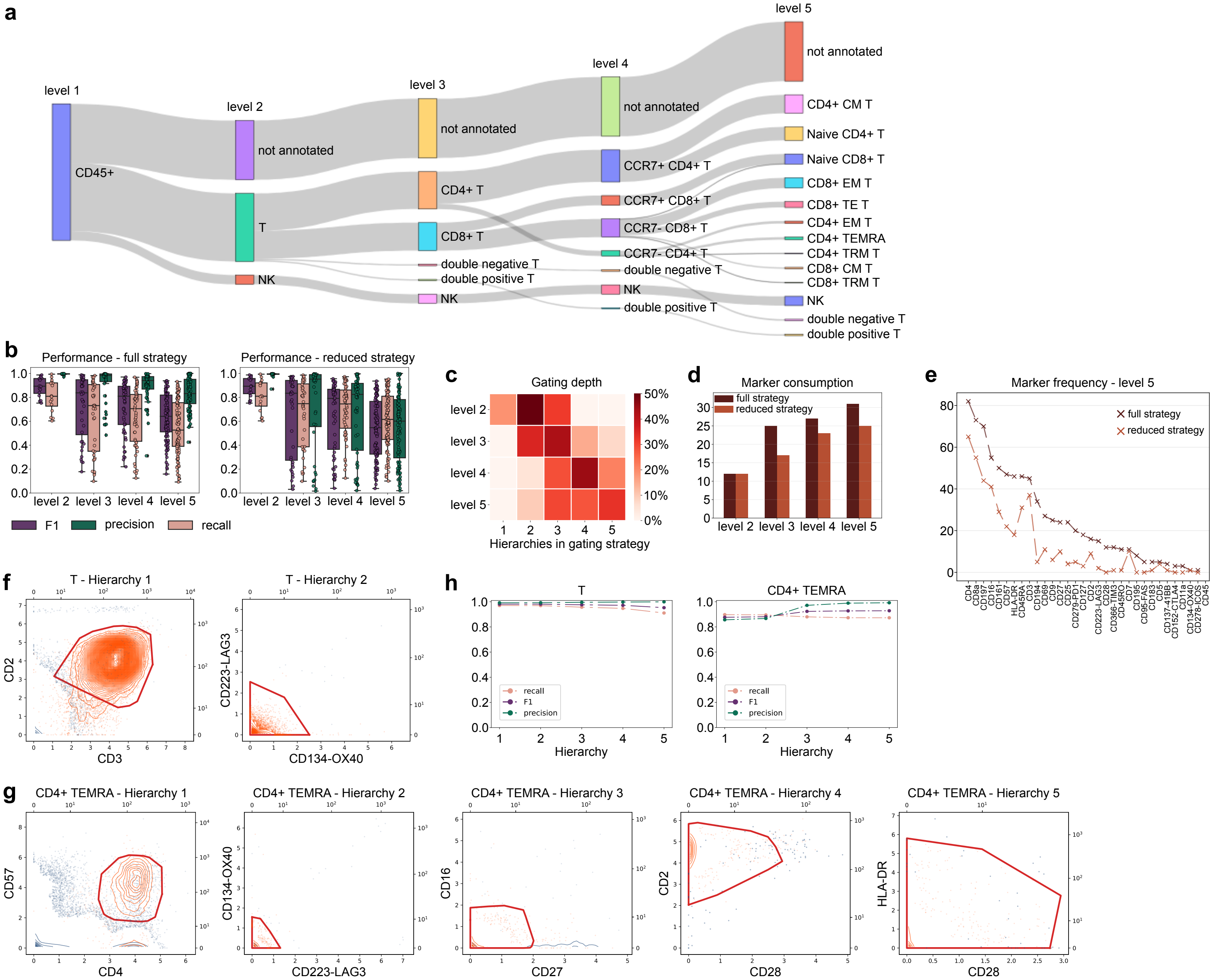

Supplementary Figure 7. ConvexGating finds gating strategies for cell populations (four annotation levels, eight samples) in healthy human bone marrow. (a) Sankey plot visualizing T cell subtyping with increasing annotation level. (b) Performance overview (F1, recall, precision) per cell type annotation level for the full strategies of up to five hierarchies (left panel) and for the reduced strategies of the first two hierarchies consisting of the initial two gating hierarchies (right panel) (boxes represent 25th percentile, median and 75th percentile, respectively, whiskers show 1.5 * interquartile range and black diamonds represent outliers). Dots indicate the performance score for a specific cell type and sample. (c) Distribution of hierarchy depth in gating strategies inferred by ConvexGating per cell type annotation level. (d) Marker usage for gating all subpopulations in all samples per cell type annotation level. The left bar (dark red) shows the marker usage for the full gating strategies while the right bar (light red) shows the marker usage for the reduced strategies. (e) Frequency of marker choice in gating strategies for cell type annotation level 5. Dark red crosses refer to the full gating strategies while light red crosses refer to the reduced gating strategies. (f) Visualization of gating strategy for T cells (annotation level 2, sample a) retrieved by ConvexGating. (g) Visualization of the gating strategy for CD4+ TEMRA cells (annotation level 5, sample a). Target cells (orange) and non-target cells (blue) are represented via scatter plots and/or contour plots per target and non-target population. (h) Overview of ConvexGating performance (F1 score, recall, precision) for T cell cluster (annotation level 2, sample a) and CD4+ TEMRA cell cluster (annotation level 5, sample a).

Supplementary Figure 8

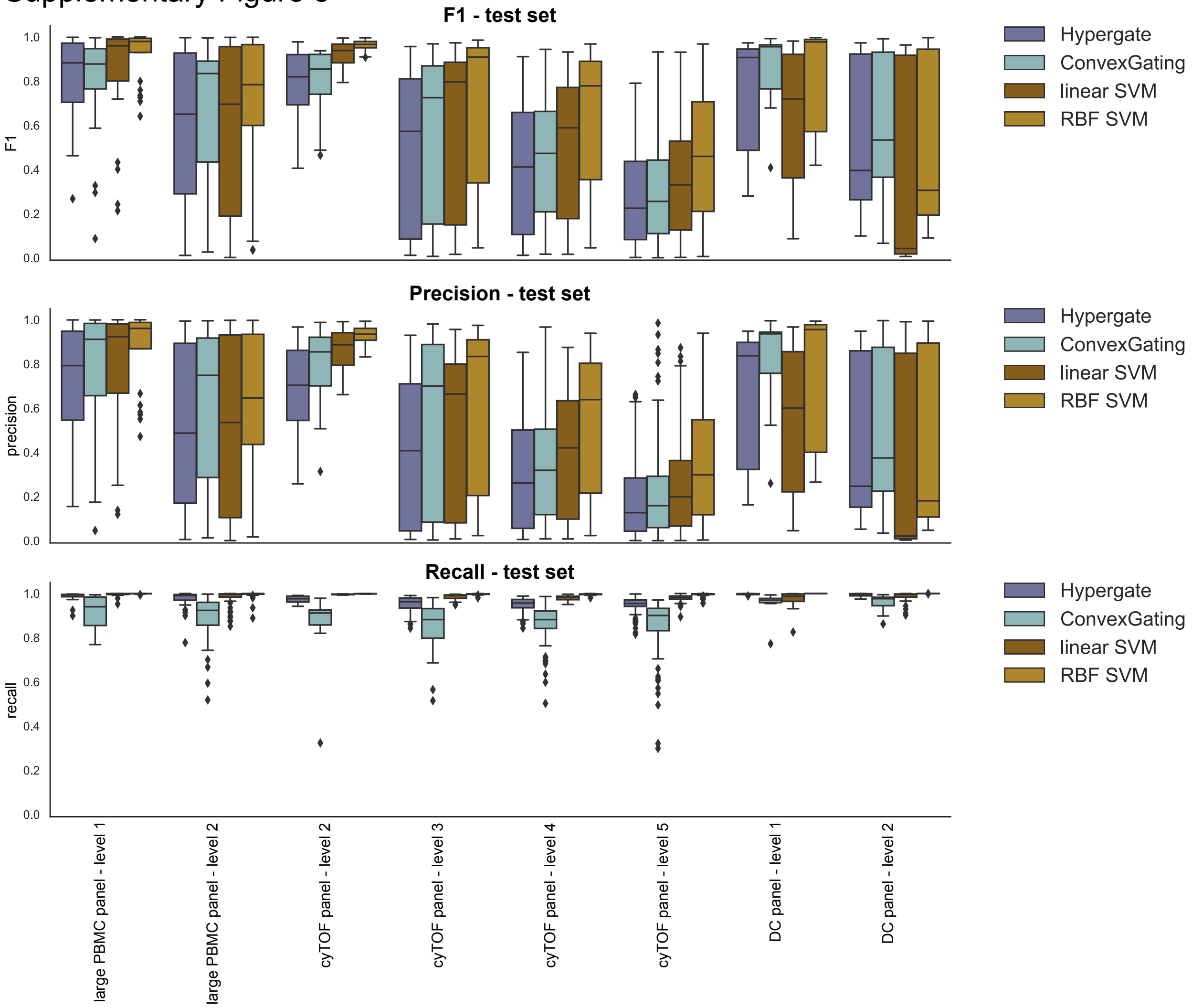

Supplementary Figure 8. Computational benchmark analysis comparing gating strategies from ConvexGating and Hypergate with machine learning classifiers. F1 score (upper panel), precision (middle panel), and recall (lower panel) boxplot results on the test set for gating strategies learned by ConvexGating and Hypergate, and for classification performance of linear SVM and RBF SVM models. Boxes represent the 25th percentile, median, and 75th percentile, respectively; whiskers indicate 1.5× the interquartile range, and black diamonds denote outliers. The benchmark analysis includes the large PBMC panel with two cell type annotation levels, the human bone marrow cyTOF panel with four annotation levels, and the DC panel with two annotation levels.

Supplementary Figure 9

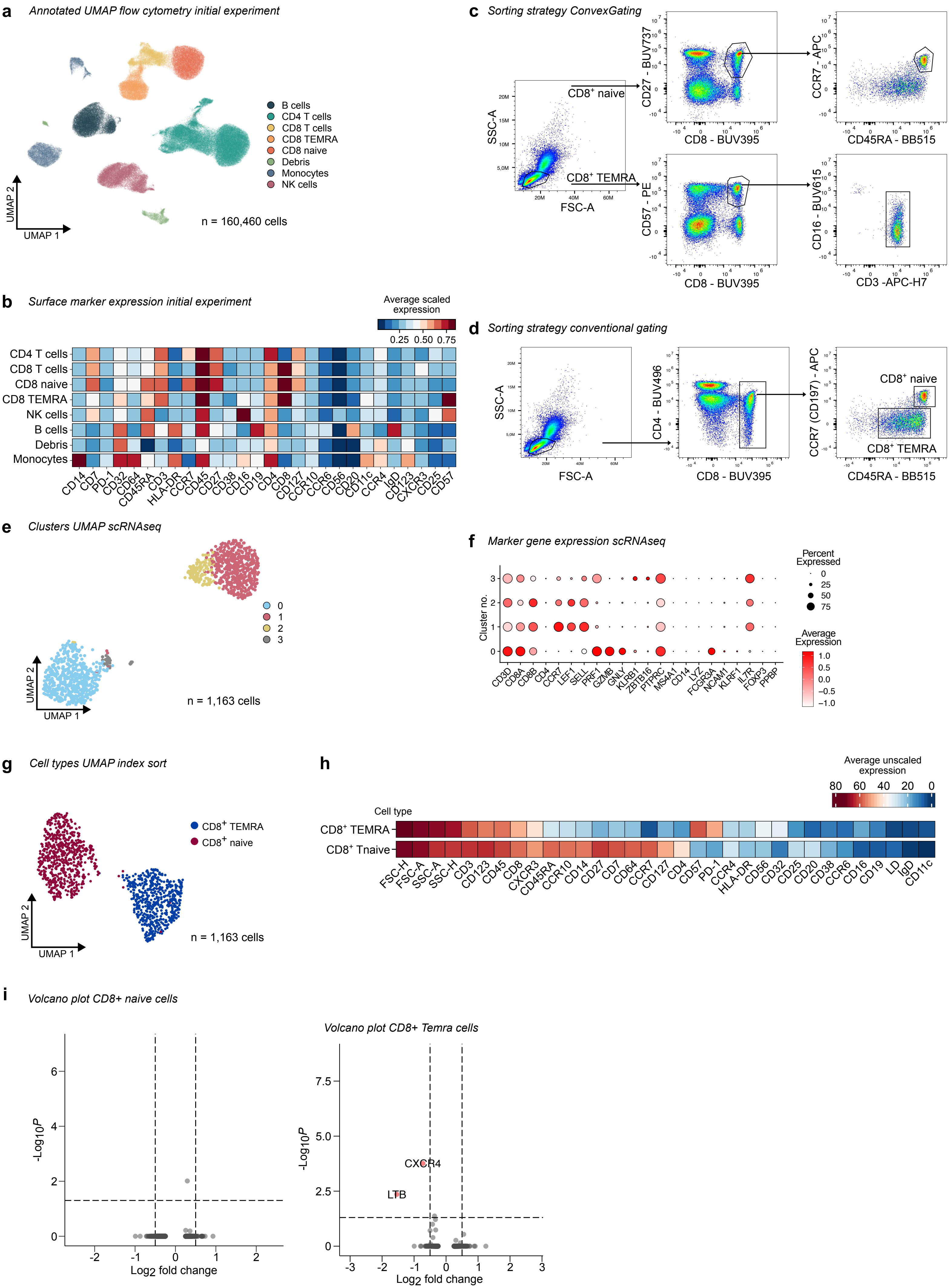

Supplementary Figure 9. Characterization of CD8+ naive and CD8+ TEMRA cell populations sorted by conventional gating and predicted by ConvexGating, respectively. (a) Annotated UMAP of PBMCs from the initial experiment that the ConvexGating strategy was derived from, n=160,460 cells. (b) Average scaled surface marker expression of cell type populations annotated in the initial experiment. (c) ConvexGating strategy for CD8+ naive cells (upper panel) and CD8+ TEMRA cells (lowerpanel) that was transferred to the cell sorter. (d) Conventional sorting strategy for CD8+ naive
cells and CD8+ TEMRA cells (both shown in the last scatter plot). (e) UMAP of the transcriptome of sorted CD8+ naive and CD8+ TEMRA cells, with colors indicating clusters obtained through unsupervised Louvain clustering. (f) Overview of average marker gene expression across clusters. (g) Annotated UMAP of the flow cytometry data of sorted CD8+ naive and CD8+ TEMRA cells. (h) Overview of average unscaled marker protein expression across clusters. (i) Volcano plots depicting differentially expressed genes comparing conventionally sorted vs. ConvexGating-sorted CD8+ naive cells (left panel) and CD8+ TEMRA cells (right panel). Dashed lines indicate a fold change of 1 (Log2 = 0.5) a p-value < 0.05. Genes that surpass these thresholds are colored in red.

Supplementary Figure 10

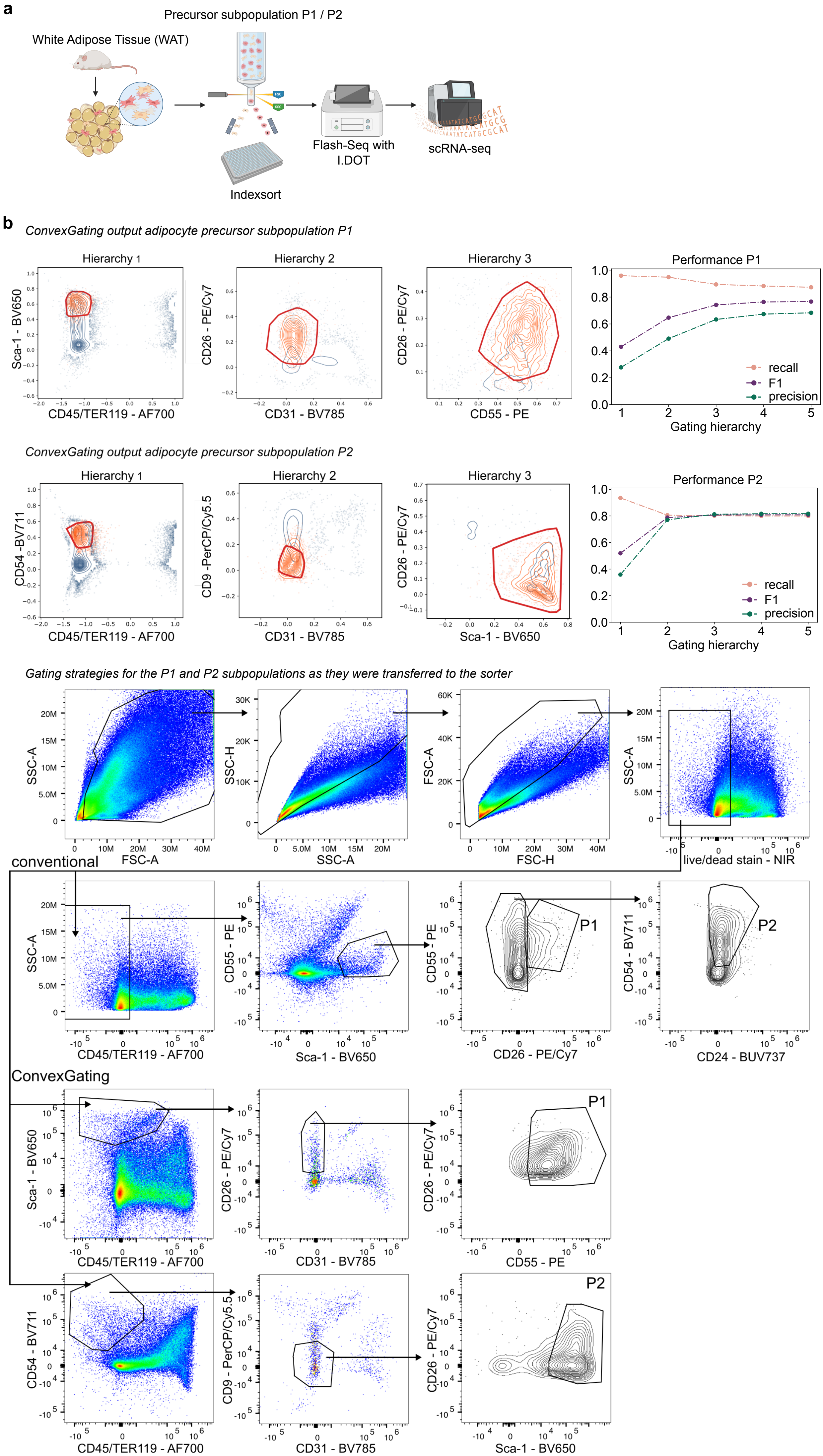

Supplementary Figure 10. Gating on White Adipose Tissue precursor cells with ConvexGating and a conventional gating strategy. (a) Schematic overview of the experimental design used to study adipocyte precursor subpopulations P1 and P2 isolated from white adipose tissue (WAT) of a high-fat diet mouse. Created with BioRender.com (https://BioRender.com/yz825ts). (b) ConvexGating results for target populations P1 (upper panel) and P2 (lower panel). (c) Gating strategy implemented on the FACSDiscover8 for index-sorting adipocyte precursor populations P1 and P2 into 384-well-plates. Initial quality control gates (size, doublet exclusion and live cell filtering) were shared between the two strategies (row 1). Conventional gating strategies for P1 and P2 are shown in row 2, while ConvexGating-derived strategies for P1 and P2 are displayed in rows 3 and 4, respectively.

Supplementary Figure 11

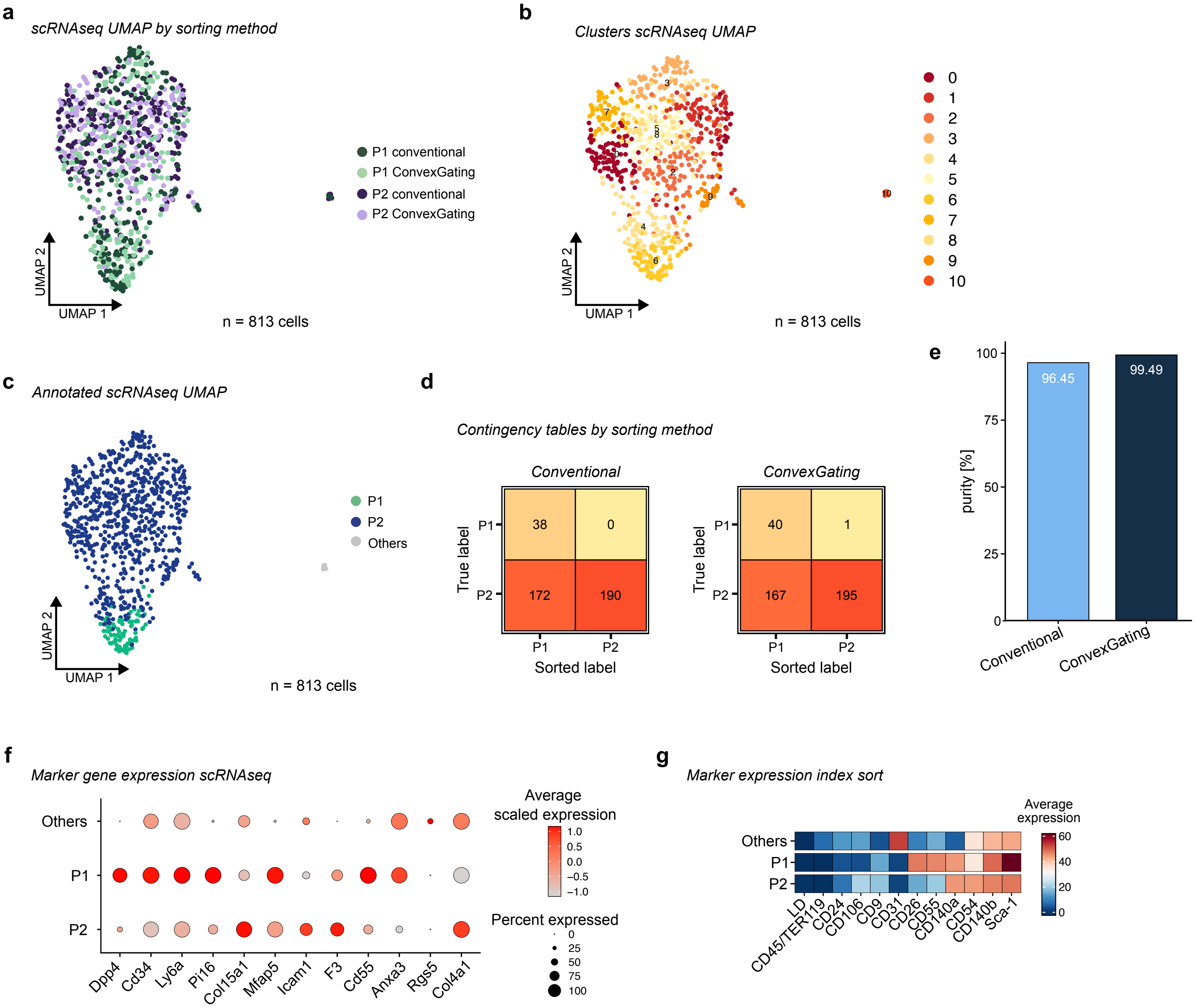

Supplementary Figure 11. Single-cell RNA sequencing validates adipocyte precursor population identities. (a) UMAP of adipocyte precursor populations P1 and P2 colored by index-sort labels (P1 and P2) of the respective gating strategies (ConvexGating and conventional). (b) Clustering of scRNA data from sorted white adipocyte tissue precursor cells. (c) Annotated UMAP of P1 and P2 precursor populations based on scRNA-seq analysis. (d) Contingency tables comparing flow cytometry sort labels with scRNA-seq-derived cell type annotations for conventional and ConvexGating strategies. scRNA-seq labels are annotated as true labels. (e) P2 population purity achieved by conventional versus ConvexGating strategies. (f) Scaled expression heatmap of marker genes distinguishing Others, P1, and P2 clusters. (g) Surface marker protein expression levels across the three annotated cell populations, as measured by index sorting.

Supplementary Figure 12

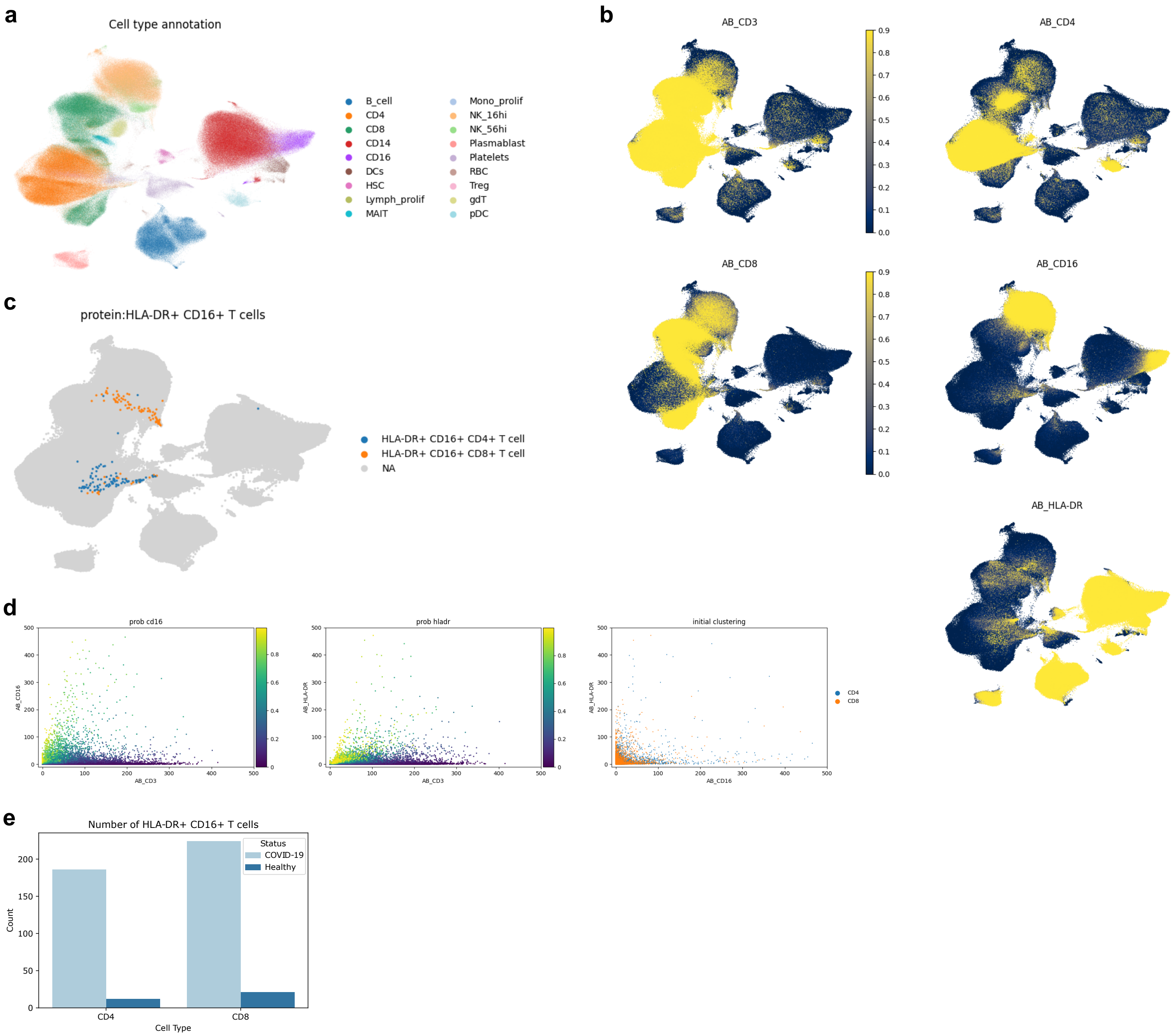

Supplementary Figure 12. Identification of CD16+ T cells in COVID-19 CITEseq data. (a-c) UMAPs showing initial cell type annotation (a), foreground probability of the markers CD3, CD4, CD8, CD16, and HLA-DR (b), and CD16+ T cell annotation (c). (d) Scatter plot of drop-in corrected anti-CD16, anti-HLA-DR and anti-CD3 abundance in the T cell compartment. Dots in the left panels are colored by foreground probability. Dots in the right panel are colored by cell type. (e) Absolute number of HLA-DR CD16+ T cells per disease diagnosis over all samples.

Supplementary Note 1

ConvexGating finds gating strategies for low and high resolution cell types in healthy human bone marrow mass cytometry data

To assess the applicability of ConvexGating to high-dimensional mass cytometry data for optimal panel design, we applied ConvexGating to extract cell populations in eight healthy human bone marrow samples measured by mass cytometry[33] with 34 markers, which focused on T cell subtyping. We used four different annotation levels as described previously[14] (**Supplementary Fig. 7 a**). In the full strategy, we allowed a maximum number of five gating hierarchies. We compared the performance of the full strategy (**Supplementary Fig. 7 b** left panel) to the performance of a reduced strategy (**Supplementary Fig. 7 b** right panel) that only relied on the initial two gating hierarchies of the full strategy. We observed a decrease in F1 score, recall and precision with increasing cell type annotation level. This aligns with the circumstance that certain T cell subpopulations such as *Double negative T cells*, *Double positive T cells* or *CD8+ CM T cells* are much more challenging to capture with a gating strategy. Specifically, mean recall (full strategy) dropped from 0.814 in level 2 to 0.548 in level 5, while mean precision (full strategy) gradually decreased with increasing annotation level (0.994 for annotation level 2 vs. 0.833 for annotation level 5). This showcases how ConvexGating aims at avoiding contamination with undesired cell types in the gated population prioritizing precision over recall. Mean F1 score (full strategy) dropped from 0.889 in annotation level 2 to 0.635 in annotation level 5. The performance of the reduced strategy closely resembled the performance of the full strategy for annotation level 2: With increasing annotation level, finer subpopulations were harder to extract when solely relying on four markers in the reduced strategy. This resulted in significantly lower mean precision scores for the reduced strategy in level 3 (0.729), level 4 (0.663) and level 5 (0.551) compared to the full strategy in level 3 (0.904), level 4 (0.894) and level 5 (0.833). On average, the number of gating hierarchies (full strategy) increased with higher cell type annotation level as finer subpopulations required more steps for extraction (**Supplementary Fig. 7 c**). Next, we inspected the number of markers that ConvexGating required for extracting all cell populations across all eight samples per cell type annotation level (**Supplementary Fig. 7 d**). In level 2, ConvexGating used 12 out of 34 possible markers for retrieving T cells and NK cells across eight different samples, where the full and the reduced strategies were based on the identical marker panel. The number of used markers increased with higher cell type annotation level. Gating strategies for cell types in level 3 were based on 25 markers, in level 4 on 27 markers and in level 5 on 31 markers (full strategy). The reduced strategies consumed fewer markers - 17 for level 3, 23 for level 4 and 25 in level 5 - at the cost of lower precision compared to the full strategies. Notably, the number of markers used by ConvexGating is higher than the number of markers used in the manual gating strategy for this dataset. To further examine the marker choice of ConvexGating, we compared how often each marker was used in the full and the reduced strategies for annotation level 5 (**Supplementary Fig. 7 e**). High marker appearance frequency in the reduced strategies indicates a prominent role in primary, broad identification of a subpopulation. Markers that were frequently used in later hierarchies play an important role in the fine-tuning steps. We observed that T cell markers CD4, CD8 and CD197 (CCR7) as well as monocyte marker CD16 appeared most frequently in both full and reduced gating strategies for annotation level 5. Notably, T cell marker

CD3 was mostly used in the initial two hierarchies (37 out 45 total appearances) which suggests a key role in the first filtering steps of T cell subpopulations. In contrast, marker CD194 (CCR4) was frequently used in the full strategies (34 times) but barely in the initial two hierarchies (5 times) which suggests an important role in the fine-tuning steps. Therefore, ConvexGating offers the possibility of panel size reduction while maintaining high precision. Markers with very few appearances in the full strategies or the reduced strategies, respectively, could be removed with very little gating performance sacrifice. We showcase the performance overview (**Supplementary Fig. 7 h**) and the complete gating strategies for level 2 T cells from sample A (**Supplementary Fig. 7 f**) and level 5 CD4+ TEMRA cells from sample A (**Supplementary Fig. 7 g**) found by ConvexGating. The optimal gating strategy for level 2 T cells could be found in two hierarchies. Notably, the precision of the gating strategy for T cells increased marginally in the second hierarchy (**Supplementary Fig. 7 h** left panel). Therefore, the T cell gating strategy could be even simplified to a single step of gating CD3 vs CD2. For level 5 CD4+ TEMRA cells, ConvexGating required five gating hierarchies for an optimal strategy, where it starts already with the markers CD4 and CD57, instead of CD3 to label CD4+ T cells (**Supplementary Fig. 7 g**). This demonstrates an important difference of ConvexGating to traditional gating strategies: While manual gating infers a full hierarchy from broadly to finely resolved cell type definitions, ConvexGating directly infers a gating strategy for a target population, regardless of potential parent populations.

Supplementary Note 2

ConvexGating strategies show better applicability on the test set across multiple scenarios compared to Hypergate

We sought to compare the performance of ConvexGating against the most recent gating tool Hypergate[26] and the SVM classifier (with linear and RBF kernel, see **methods** and **Supplementary Fig. 8**). We benchmarked all tools on three test scenarios: the DC panel with two cell type annotation levels (**Fig. 2**), the large PBMC panel with two cell type annotation levels (**Fig. 3**) and the T cell cyTOF panel of human bone marrow data with four annotation levels[14,33] (**Supplementary Fig. 7** and **Supplementary Note 1** for details). We constructed training datasets with a fixed 1:1 ratio of target to non-target cells, using the remaining data as the test set. For all samples and cell type annotation, we evaluated performance on the test set using F1 score, precision and recall (see **metrics**). We observed a higher mean F1 score for ConvexGating compared to Hypergate on all three test scenarios (**Supplementary Fig. 8** upper panel).

The higher F1 scores were based on the substantially higher precision of the gating strategies derived by ConvexGating (**Supplementary Fig. 8** mid panel). The extracted populations were less cross-contaminated with undesired non-target cells when relying on the gating strategies learned by ConvexGating. Gating strategies from Hypergate exhibited higher recall compared to ConvexGating (**Supplementary Fig. 8** lower panel), however, the higher recall did not suffice to equalize the substantially lower precision compared to gating strategies learned by ConvexGating. We further compared the performance of ConvexGating to the performance of a linear and an RBF kernel SVM classifier, which directly operated in M-dimensional marker space at the cost of interpretability and direct translatability into a gating strategy (**Supplementary Fig. 8**). ConvexGating outperformed the linear SVM classifier on the DC panel and annotation level 2 of the large PBMC panel while the linear SVM classifier achieved on average higher F1 scores on annotation level 1 of the large PBMC panel and on the cyTOF panel. ConvexGating also achieved on average higher F1 scores than the non-linear rbf SVM classifier at annotation level 2 of the DC panel. In all other tested scenarios, the non-linear rbf SVM classifier achieved the highest mean F1 scores. This shows that more flexible machine-learning models find decision boundaries that separate target cells from non-target cells more accurately. However, this comes at the expense of decision boundaries in full marker space that are difficult to interpret and lack direct transferability into a gating strategy which limits application in real-world lab routines.

Supplementary Note 3

Regularized loss.

Let $C \epsilon R^2$ be the center of the target population P^t and let H_k denote the kth hyperplane. As first regularization component we compute

$$P_1 = \frac{1}{K}\sum_{k=1}^{K} dist(C, H_k)$$

which is the average distance between the hyperplanes and target center C.
For all $k = 1, \dots, K$ $dist$ reads as

$$dist(C, H_k) = \frac{|b_k + w_k^T C|}{||w_k||}$$

where w_k and b_k denote *normal vector* and *bias* of the kth hyperplane. In our second regularization term we consider the location of the target cells relative to the hyperplanes. We exclude those target cells that exhibit the most extreme marker expression profiles in order to reduce the impact of outliers. Let

$$q(x) = \mathbf{1}\left[\left(p_{1,m_1}\ p_{1,m_2}\right)^T \leq x \leq \left(p_{99,m_1}\ p_{99,m_2}\right)^T\right]$$

where $\mathbf{1}[\cdot]$ denotes the indicator function and $p_{u,f}$ the uth percentile of targets P^t along marker f. Our second penalty term then reads as

$$P_2(x) = \frac{q(x)}{K}\sum_{k=1}^{K} \left|w_k^T x + b_k\right|.$$

We introduce penalty parameters $\lambda_1 \epsilon R$ and $\lambda_2 \epsilon R$ that control to which extent the regularization components P_1 and P_2 affect the *loss*. Our *regularized loss* then takes the form

$$L_\alpha^{reg}(x) = L_\alpha(x) + \lambda_1 P_1 + \lambda_2 P_2(x).$$

For a subset $S \subseteq P^t \cup P^{nt}$ the regularized loss over S reads as

$$L_\alpha^{reg}(S) = \frac{1}{|S|} \sum_{x \epsilon S} L_\alpha(x) + \lambda_1 P_1 + \frac{\lambda_2}{|S|} \sum_{x \epsilon S} P_2(x).$$

Supplementary Table 1

	heuristic_time_median (s)	tree_time_median (s)	svm_time_median (s)
Oetjen lvl2	0.14783895	0.401801944	11.6166972
Oetjen lvl3	0.179860234	0.633355856	20.3051076
Oetjen lvl4	0.326566339	1.229722142	40.5085475

Supplementary Table 1. Computation times for the heuristic method tree-based and SVM-based classifiers for distinguishing between target and non-target population.

Time in seconds (s) for the different annotation levels 2 (lvl2), 3 (lvl3) and 4 (lvl4).

Supplementary Table 2

	CD14-FITC B525-FITC-A	CD1c-PE Y585-PE-A	HLA-DR-PE-Cy5 Y675-PC5-A	CD16-APC R660-APC-A	CD19-20-AF700 R712-APCA700-A	CD5-BV711 V763-A
**CD14-FITC B525-FITC-A**	1	0.001924	0	0.001	0.001382	0.002144
**CD1c-PE Y585-PE-A**	0.001928	1	0.053791	0.005	0	0.002369
**HLA-DR-PE-Cy5 Y675-PC5-A**	0.000236	0.002241	1	0.09	0.069112	0.04
**CD16-APC R660-APC-A**	0	0	0.39	1	0.64	0.055
**CD19-20-AF700 R712-APCA700-A**	0	0.000137	0.047502	0.008	1	0.09
**CD5-BV711 V763-A**	0	0.00015	0.004049	0.001388	0.3	1

Supplementary Table 2. Compensation matrix of the DC panel including manual adjustment.

Supplementary Table 3

Antibody	Clone	Fluorochrome	Cat No.	Maufacturer	RRID	Dilution
CD8	RPA-T8	BUC395	563796	BD	(BD Biosciences Cat# 563795, RRID:AB_2722501)	1:100
CD4	SK3	BUV496	612936	BD	(BD Biosciences Cat# 612936, RRID:AB_2870220)	1:100
CD19	SJ25C1	BUV563	612917	BD	(BD Biosciences Cat# 612917, RRID:AB_2870202)	1:100
CD16	3G8	BUV615	751572	BD	(BD Biosciences Cat# 751572, RRID:AB_2875567)	1:100
CD38	HIT2	BUV661	612969	BD	(BD Biosciences Cat# 612969, RRID:AB_2870242)	1:100
CD27	L128	BUCV737	612829	BD	(BD Biosciences Cat# 612829, RRID:AB_2870151)	1:100
CD45	HI30	BUV805	612891	BD	(BD Biosciences Cat# 612891, RRID:AB_2870179)	1:100
CCR4 (CD194)	1g1	BV421	562579	BD	(BD Biosciences Cat# 562579, RRID:AB_2737663)	1:100
IgD	IA6-2	BV480	566138	BD	(BD Biosciences Cat# 566138, RRID:AB_2739536)	1:100
CD11c	B-ly6	BV570	630027	BD	custom conjugate, (BD Biosciences Cat# 550375, RRID:AB_393646)	1:100
CD20	2H7	BV605	747736	BD	(BD Biosciences Cat# 747736, RRID:AB_2872209)	1:100
CD56	NCAM16.2	BV650	564057	BD	(BD Biosciences Cat# 564057, RRID:AB_2738568)	1:100
CCR6 (CD196)	11A9	BV711	563923	BD	(BD Biosciences Cat# 563923, RRID:AB_2738489)	1:100
CCR10	1B5	BV750	624380	BD	custom conjugate, BD Biosciences Cat# 750937, RRID:AB_2875021	1:100
CD127	HIL-7R-M21	BV786	563324	BD	(BD Biosciences Cat# 563324, RRID:AB_2738138)	1:100
CD45RA	HI100	BB515	564552	BD	(BD Biosciences Cat# 564552, RRID:AB_2738841)	1:100
CD32	FLI8.26	BB630-P	624294	BD	custom conjugate, BD Biosciences Cat# 557333, RRID:AB_396647	1:100
CD64	10.1	BB660-P	624295	BD	custom conjugate, (BD Biosciences Cat# 555525, RRID:AB_395911)	1:100
PD1 (CD279)	EH12.1	BB700	566460	BD	(BD Biosciences Cat# 566460, RRID:AB_2744348)	1:100
CD7	M-T701	BB755-P	624391	BD	custom conjugate, (BD Biosciences Cat# 555359, RRID:AB_395762)	1:100
CD14	MP9	BB790-P	755762	BD	(BD Biosciences Cat# 755762, RRID:AB_3688077)	1:100
CD57	NK-1	PE	560844	BD	(BD Biosciences Cat# 560844, RRID:AB_2033965)	1:1000
CD25	M-A251	PE-CF594	562403	BD	(BD Biosciences Cat# 562403, RRID:AB_11151919)	1:100
CXCR3 (CD183)	1C6/CXCR3	PE-Cy5	551128	BD	(BD Biosciences Cat# 551128, RRID:AB_394061)	1:100
CD123 (IL-3)	7G3	PE-Cy7	560828	BD	custom conjugate, (BD Biosciences Cat# 554527, RRID:AB_395455)	1:100
CCR7 (CD197)	2-L1-A	APC	566762	BD	(BD Biosciences Cat# 566762, RRID:AB_2869854)	1:40
HLA-DR	L203.RMAB	R718	752501	BD	(BD Biosciences Cat# 752501, RRID:AB_2917494)	1:100
CD3	SK7	APC-H7	560176	BD	(BD Biosciences Cat# 560176, RRID:AB_1645475)	1:100

Supplementary Table 3. Information on antibodies used for the surface marker staining of human CD8 T cells. Specificity, clone, fluorochrome, catalogue number, manufacturer and final dilution for all antibodies used in the surface marker staining of human CD8 T cells (Figure 4).

Supplementary Table 4

Antibody	Clone	Fluorochrome	Maufacturer	RRID	Dilution
CD140b (Pdgfrb)	APB5	SB600	invitrogen	(Thermo Fisher Scientific Cat# 63-1402-82, RRID:AB_2784865)	1:100
Ly-6A/E (Sca-1)	D7	Brilliant Violet 650	Biolegend	(BioLegend Cat# 108143, RRID:AB_2629684)	1:200
CD31 (PECAM-1)	390	Brilliant Violet 785	Biolegend	(BioLegend Cat# 102435, RRID:AB_2810334)	1:200
CD106 (VCAM-1)	429 (MV CAM.A)	Alexa Fluor® 594	Biolegend	(BioLegend Cat# 105724, RRID:AB_2563962)	1:200
CD140a (Pdgfra)	APA5	Brilliant Violet 421	Biolegend	(BioLegend Cat# 135923, RRID:AB_2814036)	1:400
CD54 (ICAM-1)	YN/1.7.4	Brilliant Violet 711	Biolegend	(BioLegend Cat# 116143, RRID:AB_2876429)	1:400
CD55 (DAF)	RIKO-3	PE	Biolegend	(BioLegend Cat# 131804, RRID:AB_1279265)	1:400
CD24	M1/69	BUV737	BD	(BD Biosciences Cat# 612832, RRID:AB_2870154)	1:400
CD26 (DPP-4)	H194-112	PE/Cy7	Biolegend	(BioLegend Cat# 137810, RRID:AB_2564312)	1:400
CD9	MZ3	PerCP/Cyanine5.5	Biolegend	(BioLegend Cat# 124818, RRID:AB_2783077)	1:800
CD45	I3/2.3	Alexa Fluor® 700	Biolegend	(BioLegend Cat# 147716, RRID:AB_2750449)	1:800
TER119	TER-119	Alexa Fluor® 700	Biolegend	(BioLegend Cat# 116220, RRID:AB_528963)	1:800

Supplementary Table 4. Information on antibodies used for the surface marker staining of mouse white adipose tissue cells. Specificity, clone, fluorochrome, manufacturer and final dilution used in the surface marker staining of mouse white adipose tissue cells (Supplementary Figure 10 and 11).
