# Supplementary material for: ConvexGating infers gating strategies from clusters in single cell cytometry data
Source: Nat Commun. 2026 Sep 18;17:9975. doi: 10.1038/s41467-026-77360-z (PMC13588814; doi:10.1038/s41467-026-77360-z)
Supplement: Supplementary file 2 [file 41467_2026_77360_MOESM2_ESM.pdf]

nature portfolio

Corresponding author(s): Maren Büttner

Last updated by author(s): Jul 27, 2026

Reporting Summary

Nature Portfolio wishes to improve the reproducibility of the work that we publish. This form provides structure for consistency and transparency in reporting. For further information on Nature Portfolio policies, see our Editorial Policies and the Editorial Policy Checklist.

Statistics

For all statistical analyses, confirm that the following items are present in the figure legend, table legend, main text, or Methods section.

n/a	Confirmed	
☐	☒	The exact sample size (n) for each experimental group/condition, given as a discrete number and unit of measurement
☐	☒	A statement on whether measurements were taken from distinct samples or whether the same sample was measured repeatedly
☐	☒	The statistical test(s) used AND whether they are one- or two-sided<br>*Only common tests should be described solely by name; describe more complex techniques in the Methods section.*
☐	☒	A description of all covariates tested
☒	☐	A description of any assumptions or corrections, such as tests of normality and adjustment for multiple comparisons
☐	☒	A full description of the statistical parameters including central tendency (e.g. means) or other basic estimates (e.g. regression coefficient) AND variation (e.g. standard deviation) or associated estimates of uncertainty (e.g. confidence intervals)
☐	☒	For null hypothesis testing, the test statistic (e.g. F, t, r) with confidence intervals, effect sizes, degrees of freedom and P value noted<br>*Give P values as exact values whenever suitable.*
☒	☐	For Bayesian analysis, information on the choice of priors and Markov chain Monte Carlo settings
☒	☐	For hierarchical and complex designs, identification of the appropriate level for tests and full reporting of outcomes
☒	☐	Estimates of effect sizes (e.g. Cohen's d, Pearson's r), indicating how they were calculated

Our web collection on statistics for biologists contains articles on many of the points above.

Software and code

Policy information about availability of computer code

Data collection	FlowJo v. 10.8 and v.10.7.1, bcl2fastq2 v2.20, FASTQC 0.11.9, multiQC v. 1.14, kllisto 0.48.0, SnakeMake 7.20.0, tximport v1.20.0, biomaRt v2.48.3, R v 4.1.0
Data analysis	Python v. 3.8, scanpy v. 1.8.1 and v. 1.8.2, anndata v. 0.8.0, pytometry v. 0.1.2 and v 0.1.3, , dplyr v1.0.7, Seurat 4.0.4, SeuratObject v. 4.0.2, R v. 4.1.0

For manuscripts utilizing custom algorithms or software that are central to the research but not yet described in published literature, software must be made available to editors and reviewers. We strongly encourage code deposition in a community repository (e.g. GitHub). See the Nature Portfolio guidelines for submitting code & software for further information.

Data

Policy information about availability of data

All manuscripts must include a data availability statement. This statement should provide the following information, where applicable:

- Accession codes, unique identifiers, or web links for publicly available datasets
- A description of any restrictions on data availability
- For clinical datasets or third party data, please ensure that the statement adheres to our policy

CyTOF data of human bone marrow (Oetjen et al.) was obtained from FlowRepository.org (accession codes: FR-FCM-ZYQ9, FR-FCM-ZYQB).
CyTOF data set of T cells of COVID-19 patients was downloaded from https://zenodo.org/record/5771937/files/data_Tcells_annotated.csv.gz
We obtained the compensated flow cytometry data set of T cells of COVID-19 patients (Georg et al,) and was shared courtesy of the authors.

CITE-seq data set of the immune cell compartment of COVID-19 patients and healthy donors was downloaded from https://www.covid19cellatlas.org/
We obtained the compensated flow cytometry data set of PBMCs samples from five healthy human donors measured on a BD Symphony A5 instrument, encompassing 27 markers, which was previously published by Knoll et al. and was shared courtesy of the authors.
We obtained the flow cytometry measurements of whole blood staining of 6 markers specific for DCs as an in-house dataset.
The raw and processed FLASH-seq data of human CD8 T cells generated in this study have been deposited in the European Genome-phenome Archive (EGA) under accession code EGAS50000001005 (https://ega-archive.org/studies/EGAS50000001005). The data are available under restricted access due to data protection requirements under the General Data Protection Regulation (GDPR). Access can be obtained by submitting a data access request through the EGA portal using the accession code. Requests are reviewed by the study's Data Access Committee (DAC), which evaluates eligibility in accordance with the applicable ethical and legal requirements. Decisions on access requests are typically made within a few days. Approved users will be granted access for the duration of the approved research project.
The raw and processed FLASH-seq data of mouse WAT cells generated in this are available on ArrayExpress under accession number E-MTAB-17301 (10.6019/E-MTAB-17301).
Source data are provided with this paper.
Data to reproduce the figures of this manuscript are available on GitHub under https://github.com/buettnerlab/reproducibility_convex_gating and on Code Ocean under https://codeocean.com/capsule/3807581/tree.

Research involving human participants, their data, or biological material

Policy information about studies with human participants or human data. See also policy information about sex, gender (identity/presentation), and sexual orientation and race, ethnicity and racism.

Reporting on sex and gender	The human participant was male.
Reporting on race, ethnicity, or other socially relevant groupings	This information has not been collected in this study.
Population characteristics	The human participant was 37-year-old male.
Recruitment	The individuals in this study were members of the respective labs and volunteered blood draws for the purpose of this study.
Ethics oversight	For the experiments of the DC panel: Informed consent was obtained in compliance with the Declaration of Helsinki and ethical approval was obtained from the Ethics Committee of the Ludwigs-Maximilians University of Munich under the reference number 071-06 - 075-06.<br>For experiments of human CD8+ naive and TEMRA T cells: Ethical approval was obtained from the Ethics Committee of the University of Bonn under the reference 272/23-EP.<br>For mouse WAT cells: Ethical approval was obtained from the Ethics Committee of the University of Cologne under the reference A2021.080

Note that full information on the approval of the study protocol must also be provided in the manuscript.

Field-specific reporting

Please select the one below that is the best fit for your research. If you are not sure, read the appropriate sections before making your selection.

☒ Life sciences ☐ Behavioural & social sciences ☐ Ecological, evolutionary & environmental sciences

For a reference copy of the document with all sections, see nature.com/documents/nr-reporting-summary-flat.pdf

Life sciences study design

All studies must disclose on these points even when the disclosure is negative.

Sample size	Not applicable
Data exclusions	Not applicable
Replication	Not applicable
Randomization	Not applicable
Blinding	Not applicable

Reporting for specific materials, systems and methods

We require information from authors about some types of materials, experimental systems and methods used in many studies. Here, indicate whether each material, system or method listed is relevant to your study. If you are not sure if a list item applies to your research, read the appropriate section before selecting a response.

Materials & experimental systems

n/a	Involved in the study
☐	☒ Antibodies
☒	☐ Eukaryotic cell lines
☒	☐ Palaeontology and archaeology
☐	☒ Animals and other organisms
☒	☐ Clinical data
☒	☐ Dual use research of concern
☒	☐ Plants

Methods

n/a	Involved in the study
☒	☐ ChIP-seq
☐	☒ Flow cytometry
☒	☐ MRI-based neuroimaging

Antibodies

Antibodies used

A complete list of antibodies used in the respective validation experiment is provided in Supplementary Tables 2 and 3:
Antibody Clone Fluorochrome Cat No. Maufacturer RRID Dilution
CD8 RPA-T8 BUC395 563796 BD (BD Biosciences Cat# 563795, RRID:AB_2722501) 1:100
CD4 SK3 BUV496 612936 BD (BD Biosciences Cat# 612936, RRID:AB_2870220) 1:100
CD19 SJ25C1 BUV563 612917 BD (BD Biosciences Cat# 612917, RRID:AB_2870202) 1:100
CD16 3G8 BUV615 751572 BD (BD Biosciences Cat# 751572, RRID:AB_2875567) 1:100
CD38 HIT2 BUV661 612969 BD (BD Biosciences Cat# 612969, RRID:AB_2870242) 1:100
CD27 L128 BUCV737 612829 BD (BD Biosciences Cat# 612829, RRID:AB_2870151) 1:100
CD45 HI30 BUV805 612891 BD (BD Biosciences Cat# 612891, RRID:AB_2870179) 1:100
CCR4 (CD194) 1g1 BV421 562579 BD (BD Biosciences Cat# 562579, RRID:AB_2737663) 1:100
IgD IA6-2 BV480 566138 BD (BD Biosciences Cat# 566138, RRID:AB_2739536) 1:100
CD11c B-ly6 BV570 630027 BD custom conjugate, (BD Biosciences Cat# 550375, RRID:AB_393646) 1:100
CD20 2H7 BV605 747736 BD (BD Biosciences Cat# 747736, RRID:AB_2872209) 1:100
CD56 NCAM16.2 BV650 564057 BD (BD Biosciences Cat# 564057, RRID:AB_2738568) 1:100
CCR6 (CD196) 11A9 BV711 563923 BD (BD Biosciences Cat# 563923, RRID:AB_2738489) 1:100
CCR10 1B5 BV750 624380 BD custom conjugate, BD Biosciences Cat# 750937, RRID:AB_2875021 1:100
CD127 HIL-7R-M21 BV786 563324 BD (BD Biosciences Cat# 563324, RRID:AB_2738138) 1:100
CD45RA HI100 BB515 564552 BD (BD Biosciences Cat# 564552, RRID:AB_2738841) 1:100
CD32 FLI8.26 BB630-P 624294 BD custom conjugate, BD Biosciences Cat# 557333, RRID:AB_396647 1:100
CD64 10.1 BB660-P 624295 BD custom conjugate, (BD Biosciences Cat# 555525, RRID:AB_395911) 1:100
PD1 (CD279) EH12.1 BB700 566460 BD (BD Biosciences Cat# 566460, RRID:AB_2744348) 1:100
CD7 M-T701 BB755-P 624391 BD custom conjugate, (BD Biosciences Cat# 555359, RRID:AB_395762) 1:100
CD14 MP9 BB790-P 755762 BD (BD Biosciences Cat# 755762, RRID:AB_3688077) 1:100
CD57 NK-1 PE 560844 BD (BD Biosciences Cat# 560844, RRID:AB_2033965) 1:1000
CD25 M-A251 PE-CF594 562403 BD (BD Biosciences Cat# 562403, RRID:AB_11151919) 1:100
CXCR3 (CD183) 1C6/CXCR3 PE-Cy5 551128 BD (BD Biosciences Cat# 551128, RRID:AB_394061) 1:100
CD123 (IL-3) 7G3 PE-Cy7 560828 BD custom conjugate, (BD Biosciences Cat# 554527, RRID:AB_395455) 1:100
CCR7 (CD197) 2-L1-A APC 566762 BD (BD Biosciences Cat# 566762, RRID:AB_2869854) 1:40
HLA-DR L203.RMAB R718 752501 BD (BD Biosciences Cat# 752501, RRID:AB_2917494) 1:100
CD3 SK7 APC-H7 560176 BD (BD Biosciences Cat# 560176, RRID:AB_1645475) 1:100

Antibody Clone Fluorochrome Maufacturer RRID Dilution
CD140b (Pdgfrb) APB5 SB600 invitrogen (Thermo Fisher Scientific Cat# 63-1402-82, RRID:AB_2784865) 1:100
Ly-6A/E (Sca-1) D7 Brilliant Violet 650 Biolegend (BioLegend Cat# 108143, RRID:AB_2629684) 1:200
CD31 (PECAM-1) 390 Brilliant Violet 785 Biolegend (BioLegend Cat# 102435, RRID:AB_2810334) 1:200
CD106 (VCAM-1) 429 (MV CAM.A) Alexa Fluor® 594 Biolegend (BioLegend Cat# 105724, RRID:AB_2563962) 1:200
CD140a (Pdgfra) APA5 Brilliant Violet 421 Biolegend (BioLegend Cat# 135923, RRID:AB_2814036) 1:400
CD54 (ICAM-1) YN/1.7.4 Brilliant Violet 711 Biolegend (BioLegend Cat# 116143, RRID:AB_2876429) 1:400
CD55 (DAF) RIKO-3 PE Biolegend (BioLegend Cat# 131804, RRID:AB_1279265) 1:400
CD24 M1/69 BUV737 BD (BD Biosciences Cat# 612832, RRID:AB_2870154) 1:400
CD26 (DPP-4) H194-112 PE/Cy7 Biolegend (BioLegend Cat# 137810, RRID:AB_2564312) 1:400
CD9 MZ3 PerCP/Cyanine5.5 Biolegend (BioLegend Cat# 124818, RRID:AB_2783077) 1:800
CD45 I3/2.3 Alexa Fluor® 700 Biolegend (BioLegend Cat# 147716, RRID:AB_2750449) 1:800
TER119 TER-119 Alexa Fluor® 700 Biolegend (BioLegend Cat# 116220, RRID:AB_528963) 1:800

Validation

Validation data for each antibody is available on the respective vendor's website.

Animals and other research organisms

Policy information about studies involving animals; ARRIVE guidelines recommended for reporting animal research, and Sex and Gender in Research

Laboratory animals	C57bl/6n male high fat diet feeding from 3 to 20 weeks. Mice (C57Bl/6N background) were kept in IVC at a specific pathogen free facility at 22-24 °C with a 12 hours light-dark cycle with 45-65% humidity. Mice had ad libitum access to water and a high-fat diet (HFD; EF D12492-(I); Ssniff Diet).
Wild animals	N/A
Reporting on sex	This study presents a validation experiment for a newly developed tool, in which a single sample was used to demonstrate its usability and technical validity. As the primary objective was to evaluate the performance and reliability of the tool rather than to investigate biological or population-level effects, only one sample, and consequently one sex, was included in this analysis. Sex-based analysis was therefore not applicable in this context.
Field-collected samples	N/A
Ethics oversight	Ethical approval was obtained from the Ethics Committee of the University of Cologne under the reference A2021.080.

Note that full information on the approval of the study protocol must also be provided in the manuscript.

Plants

Seed stocks	N/A
Novel plant genotypes	N/A
Authentication	N/A

Flow Cytometry

Plots

Confirm that:

- [x] The axis labels state the marker and fluorochrome used (e.g. CD4-FITC).
- [x] The axis scales are clearly visible. Include numbers along axes only for bottom left plot of group (a 'group' is an analysis of identical markers).
- [x] All plots are contour plots with outliers or pseudocolor plots.
- [x] A numerical value for number of cells or percentage (with statistics) is provided.

Methodology

Sample preparation

Staining of whole blood of healthy donors (DC panel):
The antibody panel was designed to analyze monocytes and cDC2 subsets32. 100 µl of heparinized venous blood from three apparently healthy donors was used and subjected to erythrocyte lysis using Q-Prep (Coulter). After centrifugation, the cell pellet was resuspended in 100 µl staining-buffer (PBS + 2% FCS + 2 mM EDTA) and cells were incubated with CD1c-PE, CD5-BV711, CD14-FITC, CD16-APC, HLA-DR-PE-Cy7, CD19-AF700, and CD20-AF700 for 20 min in the dark on ice. After washing, the cells were resuspended in a staining-buffer and analyzed using a Beckman-Coulter CytoFLEX LX flow cytometer. A compensation matrix was generated using single stained Versa Comp compensation beads (Coulter) and manually adjusted. Fcs-files were analyzed using FlowJo v10.8. After gating on singlets plus HLA-DR+ non-B cells, monocyte subsets were defined based on expression of CD14 and CD16, and cDC2 subpopulations were identified using CD1c, CD14, and CD5.
Isolation of human PBMCs to sort CD8+ naive and CD8+ TEMRA cells:
We isolated PBMCs of one healthy donor from EDTA-treated peripheral blood via Pancoll density centrifugation at 800 xg for 20 minutes at room temperature without break. Cells were collected with a Pasteur pipette and washed twice with MACS buffer (1x PBS with 0.5% BSA and 0.074% EDTA). Then, we stained the cells with the surface antibodies listed in Supplementary Table 2 at 4°C for 30 minutes. We washed twice and proceeded with FACS sorting.
Library preparation for CD8+ naive and CD8+ TEMRA cells
We FACS-sorted stained CD8+ naive and CD8+ TEMRA cells directly into 384-well plates pre-filled with 1 µL of lysis buffer containing 0.2% Triton X-100, RNase inhibitor, dithiothreitol, betaine, and supplied with dNTPs, SMART-dT30VN primer, dCTP, and FLASH-seq-Template Switching Oligo (FS-TSO), using the BD FACSDiscover S8. Plates were sealed, snap-frozen on dry ice and stored at −80°C until further processing. We prepared libraries following the FLASH-seq protocol as described by Picelli and Hahaut (2023)35 with minor modifications. Specifically, after a 3-minute incubation at 72°C, reverse transcription

polymerase chain reaction (RT-PCR) was performed by adding 4 µL of RT-PCR mix containing Superscript IV (Thermo Fisher Scientific) to each well using the I.DOT Non Contact dispenser (Dispendix). The RT-PCR program consisted of: 60 minutes at 50°C, 3 minutes at 98°C, followed by 21 cycles of (20 seconds at 98°C, 20 seconds at 67°C, and 6 minutes at 72°C). Amplified products were purified using the C.WASH noncontact centrifugal plate washer (CYTENA) for the beads clean up step. After plate normalisation by adding nuclease-free water to, in average, a final concentration of 200pg/ µL , 0.5 µL cDNA underwent tagmentation and enrichment using the Tagmentase (Tn5 transposase- unloaded, Diagenode). Loading of the Tn5 was conducted as described by Scholz et al.44. For preparing the final sequencing libraries, a working dilution of 0.01 µM was employed; however, optimization might be required depending on the specific activity of each batch of Tn5.

The final libraries were quantified using a Qubit Fluorometer with the Qubit dsDNA HS Assay (Thermo Fisher) and the fragment size distribution was measured using the Agilent high sensitivity D1000 assay on a TapeStation 4200 system (Agilent Technologies). Sequencing was performed in paired-end mode (101 cycles) on a NovaSeq 6000 system (Illumina) with NovaSeq S1 v 1.5 (200 cycles) chemistry. Raw sequencing data were demultiplexed using bcl2fastq2 v2.20.

Isolation and processing of visceral adipose tissue

The isolation of progenitor cells from the stromal vascular fraction (SVF) followed the protocol outlined by Sieckmann et al. In brief, inguinal white adipose tissue (WAT) from high-fat diet-fed mice was excised, finely chopped, and enzymatically digested using 2 mg/mL collagenase II (Merck) and 15 kU/mL DNAse I (PanReac AppliChem) in PBS containing 0.5% bovine serum albumin (BSA; Sigma) at 37 °C under continuous agitation. The enzymatic reaction was terminated by adding AT buffer (PBS with 0.5% BSA). Following digestion, the cell suspension was filtered through a 100-µm strainer (Corning) and centrifuged at 500 × g for 10 min. The supernatant, which contained mature adipocytes, was removed, while the pellet comprising the stromal vascular fraction was resuspended in red blood cell lysis buffer (BioLegend) and incubated for 2 min at room temperature. This lysis step was halted by the addition of AT buffer, followed by centrifugation at 500 × g for 10 min. The isolated cells were subsequently kept in BD Omics Guard at 4°C overnight. On the next day, cells were washed, then stained with the surface antibodies listed in Supplementary Table 3 and incubated at 4°C for 30 minutes. Then, index sorting on a BD FACSDiscover S8 was performed.

Instrument

BD FACSDiscover S8, Beckman-Coulter CytoFLEX LX

Software

FlowJo v. 10.8 and 10.7.1, pytometry v. 0.1.2, Python v 3.8
CytExpert (Beckman-Coulter) Version 2.6

Cell population abundance

Cell type abundance was determined in two ways: One follows a manual gating strategy and the second one employs community-based clustering. We compared the results of manual gating strategies with clustering based results as a part of the publication.

Gating strategy

For the identification of subsets of human DCs related to Fig. 2 in the manuscript, we used a specifically designed 6-marker panel tailored to identify cDC2 subsets (CD1c-PE, CD5-BV711, CD14-FITC, CD16-APC, HLA-DR-PE-Cy7, CD19-AF700, and CD20-AF700) (see Heger et al.). The gating strategy is presented in Supplementary Fig. 2 starting with FSC-A/SSC-A.

For the identification of human CD8+ T naive and TEMRA cells related to Fig. 4 in the manuscript, we used the following markers: CD8-BUV395, CD4-BUV496, CD45RA-BB515, CCR7 (CD197)-APC, CD57-PE, CD27-BUB737. The gating strategies are presented in Supplementary Fig. 9 C-D starting with FSC-A/SSC-A.

For the identification of mouse progenitor cell populations P1 and P2 of white adipose tissue related to Supplementary Figures 10-11, we used the markers CD45-AF700, TER119-AF700, CD55-PE, Sca-1-BV650, CD26-PE/Cy7, CD24-BUV737, CD54-BV711, CD9-PerCP/Cy5.5,CD31-BV785, CD140b-SB600, CD106-AF594, CD140a-BV421. The gating strategies are presented in Supplementary Fig. 10 C starting with FSC-A/SSC-A.

☒ Tick this box to confirm that a figure exemplifying the gating strategy is provided in the Supplementary Information.
