# Supplementary material for: ConvexGating infers gating strategies from clusters in single cell cytometry data
Source: Nat Commun. 2026 Sep 18;17:9975. doi: 10.1038/s41467-026-77360-z (PMC13588814; doi:10.1038/s41467-026-77360-z)
Supplement: Supplementary file 3 [file 41467_2026_77360_MOESM3_ESM.pdf]

nature portfolio

Peer Review File

ConvexGating infers gating strategies from clusters in single cell cytometry data

Corresponding Author: Dr Maren Büttner

This file contains all reviewer reports in order by version, followed by all author rebuttals in order by version.

Attachments originally included by the reviewers as part of their assessment can be found at the end of this file.

Version 0:

Reviewer comments:

Reviewer #1

(Remarks to the Author)
Summary:

The author proposed to a new gating strategy called ConvexGating for single cell cytometry data and showed its usage in several real data examples. It follows from cell clustering results and can be a good alternative to manual gating for more data-driven gating strategy. The method is novel, however, the paper failed to convey a consistent message about the strength and best usage of their method throughout all examples. In addition, I also have more technical questions on reliance on linear boundary (where non-linear SVM seems to perform better), the effect of the two regularizations terms in the objective function, etc. Here are my comments:

1. I have some concerns on how consistent is the strength of ConvexGating throughout the many examples presented. In showcase 1, the authors used manual gating as optimal and compared ConvexGating (following clustering) to it (and some variation in those with more overlap vs less overlap). Then in showcase 2, the original gating results was used as the gold standard (when manual gating is not feasible?), and the results for different cell populations varied. In showcase 4, ConvexGating is compared to other gating methods Hypergate and SVM, and it is not the best performer with tradeoff between high recall and precision. In the other showcase, ConvexGating demonstrates higher precision for cell sorting but is not compared to any other gating methods. Is ConvexGating regarded as a good alternative to manual gating strategy when it is not available or it has the flexibility to follow from clustering methods or something else? Where is its main strength lie in as compared to other methods? And is this strength context-dependent?

2. As a gating strategy, ConvexGating follows from cell clustering results, with cell populations already defined. Given there are many machine learning methods used to cluster cell populations, is the method very sensitive to the clustering methods being used and the clustering results?

3. How well the gating strategy (and the clustering as well) performs seems to be highly dependent on the marker gene selection. The authors mentioned in one showcase that ConvexGating supports optimal design of marker panels. ConvexGating selects marker first (the two markers with the highest heuristic value for the hierarchy) then learn the gate location. How sensitive is the method to the number of markers (or combinations) selected (is it only one pair with highest heuristic value picked, then how about joint effect of multiple marker pairs that can the separate the target vs non-target population distributions well)? What's the difference between reduced vs full strategy in terms of the method (seems at each hierarchy you are not only restricted to one pair of markers)? Additionally, how does the method ensure the feature selection and gate locating well align at each hierarchy?

4. In Fig S7, as the authors mentioned, the non-linear RBF SVM performed better than ConvexGating, basically this shows the boundary between cell populations is essentially non-linear. Then why the gating strategy has to use simple geometric shapes while knowing the true boundary is not simple? The 2D marker space is unique to ConvexGating, there could be variability between different cell populations that are not simply visible using two most discriminative markers.

5. Following the above question, in the Methods for the ConvexGating Algorithm, the way gate is defined is very similar to

the way SVM defines the boundary, but more on a linear sense, if the authors could utilize the kernel function as in SVM and should be extensible to non-linear case, which is closer to real data scenario.

6. In Methods section regularized loss, the authors include two problem specific regularization terms P1 and P2 (Supp Note 3) to tighten the gate, both terms look at distance between target population to the hyperplane (one for center, the other for all cells). In the objective function you minimize, you will penalize on larger P1 and P2, i.e. penalize larger distances, is this against a clustering or classification tuition where you wish to maximize the margin or enlarge the distance? Also, are there tradeoff between the two penalization terms? I thought they usually agree with each other.

(Remarks on code availability)

Reviewer #2

(Remarks to the Author)
This article by Friedrich et al., describe ConvexGating, a new algorithm implemented in a Python R package to derive gating strategies targeting user-input cell populations (e.g. cell clusters). The proposed method relies on the heuristic selection of K pairs of markers (designated as a "hierarchy" in the article) based on expression quantiles computed on the target cell population and the rest of the cells. For each hierarchy, a set of K hyperplanes is then optimized by stochastic gradient descent, optimizing for a weighted cross-entropy loss function and with regularization terms. Finally, a convex hull is applied to further adjust the convex polygon to target cells, and the process is repeated for the next level of the hierarchy. The authors demonstrate the use of ConvexGating on a number of datasets covering a wide range of technologies (flow cytometry, mass cytometry, CITE-seq, spectral flow cytometry).
In my opinion, this type of research is useful to make experimental design in flow cytometry more data-driven. There are however a number of limitations in this study, notably the performance of the method is possibly overstated. Consistently testing the gating strategies on held-out data and independent samples appear important as gating strategies that would not generalize would have little practical utility.

Major points:

1.All results of the ConvexGating algorithm are reported on the sample on which the algorithm was ran. This carries a risk of overfitting. The authors need to show cross-sample performance (training on a sample and testing on another), as well as train/test split performance on single samples. This seems relevant since the choice of markers for the different levels of the hierarchy varies substantially acros samples, for instance in supplementary fig 3A.
2.In many instances, it seems that markers are selected (with the heuristic "step 1" described in the method) but end up having little influence on the final output. For instance, when gating on pDCs in fig 3h, the authors report that ConvexGating identified CD57-CD123+CD13-CD45RA+ as the phenotype of pDCs. But when examining Fig 3H, it appears that CD57- has no effect to gate on pDCs since CD123+ cells are all CD57- ? Worryingly, the contribution of CD57 appears to be mostly to gate out CD123+ events that are below the axis for CD57 intensity. This suggests that CD57 does not meaningfully, but rather artifactually, contribute to the identification of pDCs in this instance.
3.All the comparisons to previous methods such as Hypergate should be done with each method trained on a random subsample of the data and evaluated on left-out events to account for potential overfitting. Additionally, the authors report to have subsetted 2,000 positive events and 2,000 negative events before running Hypergate, and state that this is the recommended procedure. I do not think this is the recommended procedure nor that this is a fair comparison. The two methods should be ran and evaluated on the exact same data.
4.In supplementary fig 3E/F/G, the authors show the output of ConvexGating for dendritic cells subsets. The first step involves a very tiny convex polygon drawn on CD1c+HLA-DR+ cells, resulting in very high precision and low recall. Can the authors show that the learnt gating strategies for DC subsets actually apply to the other samples ?
5.The authors use ConvexGating to define a gating strategy for Temra and Tnaive cells, and compare it for sorting to a manual gating strategy. This manual gating strategy is however not shown. The methods mention "canonical markers" but these are not explicitly mentioned.
6.After sorting Temra and Tnaive cells, the authors report the average protein expression of the 5 identified cell transcriptomic clusters based on the index sort data (Sup fig 8d). The data is scaled column-wise which does not allow interpreting the raw expression data. Worryingly, many markers appear "differentially-expressed" on this heatmap across Temra and Tnaive cells that should not be expressed in these cell population (e.g. CD56). This could be due to the scaling, but may highlight compensation issues. In particular, the CD8 - 'BUC395' versus CD27 - BUV805 plot shown in fig S8A has CD27+ CD8- events that are leaning towards the negative, which suggests overcompensation.
7.The last panel of figure S8A (CD3-APC-H7 versus CD16-BUV615) features a blurry background which abruptly stops horizontally, and absolutely no event outside of the second gate. I am therefore confused about what is being shown. Has the image been edited in some way ? Why is this gate used although no events are being excluded ?
8.2D dotplots are shown with solid colors (e.g. fig 2d, 2h, 3b) or as contour plots with no outliers (e.g. fig 5). This contradicts a statement made in the nature portfolio reporting summary checklist ("All plots are contour plots with outliers or pseudocolor plots").
9.The plots shown are in transformed scale using biexponential transformations. Original scale tickmarks are not present. The biexponential plots have a number of free parameters that are not reported, so it becomes difficult to evaluate the data

shown. The checklist states “The axis scales are clearly visible” which although ambiguous suggests that decades for raw intensities should be reported ?

Minor points:
1.While it is not the main focus of the paper, there are many statements that advocate for clustering-based phenotyping over manual gating. While the authors advocate for clustering, a fair acknowledgements of manual gating advantages over clustering should also be stated (e.g. that the phenotype is informed by previous knowledge and generates interpretable cell labels)
2.Fig 1B puts a lot of emphasis on the convex hull but the core of the method seems to be the stochastic gradient descent step
3.In Fig 3C, the authors compare manual labels generated by multiple independently-generated manual gating strategies to published labels with normalized mutual information. It is not clear what the benefit of NMI is compared to accuracy in this setting ?
4.Figure 4 is described after figure 5 in the text
5.Line 482 : typographical error, break instea of brake
6.Line 592 : typographical error (104 instead of 10,000)
7.In the methods, the authors mention bi-exponential transformations but the coefficients used is not clear
8.The authors advocate for clustering-based phenotyping as a more unbiased strategy, but the methods describe the use of a “split and merge” strategy. Doesn’t this step introduce arbitrariness in the analysis ?
9.I do not understand the equation stated in line 752 : q_f are length-3 vectors, so I am not sure how to interpret the modulus. Are the authors computing the L1 norm ?

(Remarks on code availability)
I've installed the package and followed the tutorial, which illustrates the use of convexgating on 5000 simulated cells.

A tutorial on real data starting from raw data and showing all the upstream steps (e.g. compensation, transformation, clustering of the data) and application of convexgating would be useful.

Reviewer #3

(Remarks to the Author)
Friedrich et al present ConvexGating, a method for learning gating strategies from cytometry data using convex shapes in 2D marker space. The approach is designed to mimic (and improve upon) manual gating while improving reproducibility and reducing operator bias. The authors apply it across multiple cytometry platforms and validate its performance using transcriptomic data and comparisons to other methods. The overall concept is well-motivated and the results are promising. I found the work to be thoughtfully executed, though I have several suggestions that could help clarify certain aspects and strengthen the evaluation.

Major suggestions:

The heuristic for selecting marker pairs, based on differences in distribution percentiles between target and non-target populations, is intuitive and computationally efficient. However, it may overlook more informative combinations. Including a brief comparison to alternative selection criteria, such as mutual information or other discriminative metrics, could help validate the robustness of this choice.
The authors show that there is considerable variability in human-labeled gating and position ConvexGating as a viable, standardized, algorithmic alternative. However, the manuscript does not include a direct comparison of ConvexGating’s performance against human gating in reproducing a gold standard. Including such a comparison would provide stronger support for the claim that ConvexGating can effectively replace or standardize manual gating workflows.
Most of the validation focuses on immune populations, particularly CD8+ T cells, and primarily within peripheral blood or related contexts. While these are important use cases, it would be helpful to assess whether ConvexGating generalizes to a broader range of tissues and cell types, including non-immune or more heterogeneous populations. This would better demonstrate its applicability across diverse biological settings.

Minor suggestions:
Figure 1 would benefit from a clearer schematic illustrating how the convex gates evolve during training.
It would be helpful to provide an estimated runtime for ConvexGating on a typical dataset (e.g., 50k cells with 30 markers).

(Remarks on code availability)
Code is well documented

Version 1:

Reviewer comments:

Reviewer #2

(Remarks to the Author)
In this revised manuscript, Friedrich et al. have substantially improved the presentation of their results, notably by strengthening the quantitative evaluations of their method by clearly evaluating on held out cells from the same sample, cells from a distinct sample but generated in the same experimental fashion, or on data from a distinct and independently generated sample. The authors have also satisfyingly adressed most of my previous concerns or corrected me in some instances.

I am however still unconvinced by two of their answers to my previous comments.

1.My previous point #2 was:

“In many instances, it seems that markers are selected (with the heuristic “step 1” described in the method) but end up having little influence on the final output. For instance, when gating on pDCs in fig 3h, the authors report that ConvexGating identified CD57-CD123+CD13-CD45RA+ as the phenotype of pDCs. But when examining Fig 3H, it appears that CD57- has no effect to gate on pDCs since CD123+ cells are all CD57- ? Worryingly, the contribution of CD57 appears to be mostly to gate out CD123+ events that are below the axis for CD57 intensity. This suggests that CD57 does not meaningfully, but rather artifactually, contribute to the identification of pDCs in this instance. “

The authors replied by showing that removing CD57 indeed helped ConvexGating identify a gating strategy with higher performance. The way I see it in this example CD123+ cells are way scarcer than CD123- cells and the CD57+ gate is a way for the model to account for that, although examination of figure 3H clearly shows pDCs are CD57-. My argument was that in this instance ConvexGating identified CD57 as a discriminating marker for pDCs when it was not, and the manual exclusion analysis that the authors performed seems to confirm it. I thus think this warrants a discussion on the performance of this approach and that marker selection potentially needs to be refined manually ?

2.My previous point #3 was :
“Additionally, the authors report to have subsetted 2,000 positive events and 2,000 negative events before running Hypergate, and state that this is the recommended procedure. I do not think this is the recommended procedure nor that this is a fair comparison. The two methods should be ran and evaluated on the exact same data.

The authors replied in their rebuttal by quoting the Hypergate paper to justify their choice.
In my opinion, metrics such as Precision and Recall will heavily depend on the balance of classes (% of positive and negative cells in this case), which I think justifies downsampling under stable proportions (as it appears to be done for ConvexGating).
Additionally, I would argue that this is what the authors of the Hypergate paper performed, as they mention “we also sampled a corresponding proportion of negative events.” (proportion, not count). I am the first author of that article so I apologize if that sentence was confusing, but I believe downsampling while maintaining class proportions is more appropriate in this instance. In any case, I don’t think that comparing methods on datasets that were subsampled in different ways makes sense in this context, especially when the metric is F1 or its constituents.

I thus argue that the comparisons to Hypergate should be redone on the same data subsamples.

3.In my previous Minor points #5 I was pointing at a typographical error that the authors could not find. I apologize if my comment lacked clarity, the sentence that needs fixing is in the “Isolation of human PBMCs to sort CD8+ naive and CD8+ TEMRA cells” section of methods, specifically the sentence “We isolated PBMCs of one healthy donor from EDTA-treated peripheral blood via Pancoll density centrifugation at 800 xg for 20 minutes at room temperature without break”. I was saying that it should likely be “without brake” and not “without break” although this is not my main area of expertise.

Otherwise, the authors satisfyingly adressed my comments. The two major points above still need to be discussed or redone.

Etienne Becht

(Remarks on code availability)

Reviewer #3

(Remarks to the Author)
The authors sufficiently addressed all my comments.

(Remarks on code availability)

Reviewer #4

(Remarks to the Author)
I sincerely appreciate the authors' great revision work. The authors have successfully addressed most of the concerns proposed by Reviewer 1. However, here are some points that are not fully discussed.

#1: Where does ConvexGating's main strength lie as compared to other methods?
Many clustering (unsupervised) and auto-gating (supervised or semi-supervised) methods are available for mass and cytometry data gating. Some of these methods apply machine learning approaches and employ markers (prior knowledge) for cell sorting. What are the major novelties and strengths of the proposed ConvexGating? Does ConvexGating present superior performance when quantitatively compared with existing methods? I believe this should be the major information that is expected to be highlighted.

(Remarks on code availability)
The README page is clear, with a user-friendly example demo.

Version 2:

Reviewer comments:

Reviewer #2

(Remarks to the Author)
The authors have addressed all my previous comments.
I have no further remarks

(Remarks on code availability)

Open Access This Peer Review File is licensed under a Creative Commons Attribution 4.0 International License, which permits use, sharing, adaptation, distribution and reproduction in any medium or format, as long as you give appropriate credit to the original author(s) and the source, provide a link to the Creative Commons license, and indicate if changes were made.
In cases where reviewers are anonymous, credit should be given to 'Anonymous Referee' and the source.
The images or other third party material in this Peer Review File are included in the article's Creative Commons license, unless indicated otherwise in a credit line to the material. If material is not included in the article's Creative Commons license and your intended use is not permitted by statutory regulation or exceeds the permitted use, you will need to obtain permission directly from the copyright holder.
To view a copy of this license, visit https://creativecommons.org/licenses/by/4.0/

Point-by-point response

Reviewers' comments:

Reviewer #1 (Remarks to the Author):

Summary:

The author proposed to a new gating strategy called ConvexGating for single cell cytometry data and showed its usage in several real data examples. It follows from cell clustering results and can be a good alternative to manual gating for more data-driven gating strategy. The method is novel, however, the paper failed to convey a consistent message about the strength and best usage of their method throughout all examples. In addition, I also have more technical questions on reliance on linear boundary (where non-linear SVM seems to perform better), the effect of the two regularizations terms in the objective function, etc. Here are my comments:

1. I have some concerns on how consistent is the strength of ConvexGating throughout the many examples presented. In showcase 1, the authors used manual gating as optimal and compared ConvexGating (following clustering) to it (and some variation in those with more overlap vs less overlap). Then in showcase 2, the original gating results was used as the gold standard (when manual gating is not feasible?), and the results for different cell populations varied. In showcase 4, ConvexGating is compared to other gating methods Hypergate and SVM, and it is not the best performer with tradeoff between high recall and precision. In the other showcase, ConvexGating demonstrates higher precision for cell sorting but is not compared to any other gating methods. Is ConvexGating regarded as a good alternative to manual gating strategy when it is not available or it has the flexibility to follow from clustering methods or something else? Where is its main strength lie in as compared to other methods? And is this strength context-dependent?

We thank the reviewer for their constructive feedback regarding the performance consistency of ConvexGating across different showcases. We appreciate the opportunity to clarify ConvexGating's intended positioning and context-dependent strengths.

Primary Purpose and Niche: ConvexGating addresses a specific technical gap in the cytometry workflow (**Figure 1 A**). While clustering methods perform on par or excel at cell type identification and SVMs achieve superior classification performance, neither can be directly implemented on cell sorters, which exclusively operate with 2D convex gating strategies. ConvexGating bridges this gap by translating clustering results into compatible gates for cell sorting.

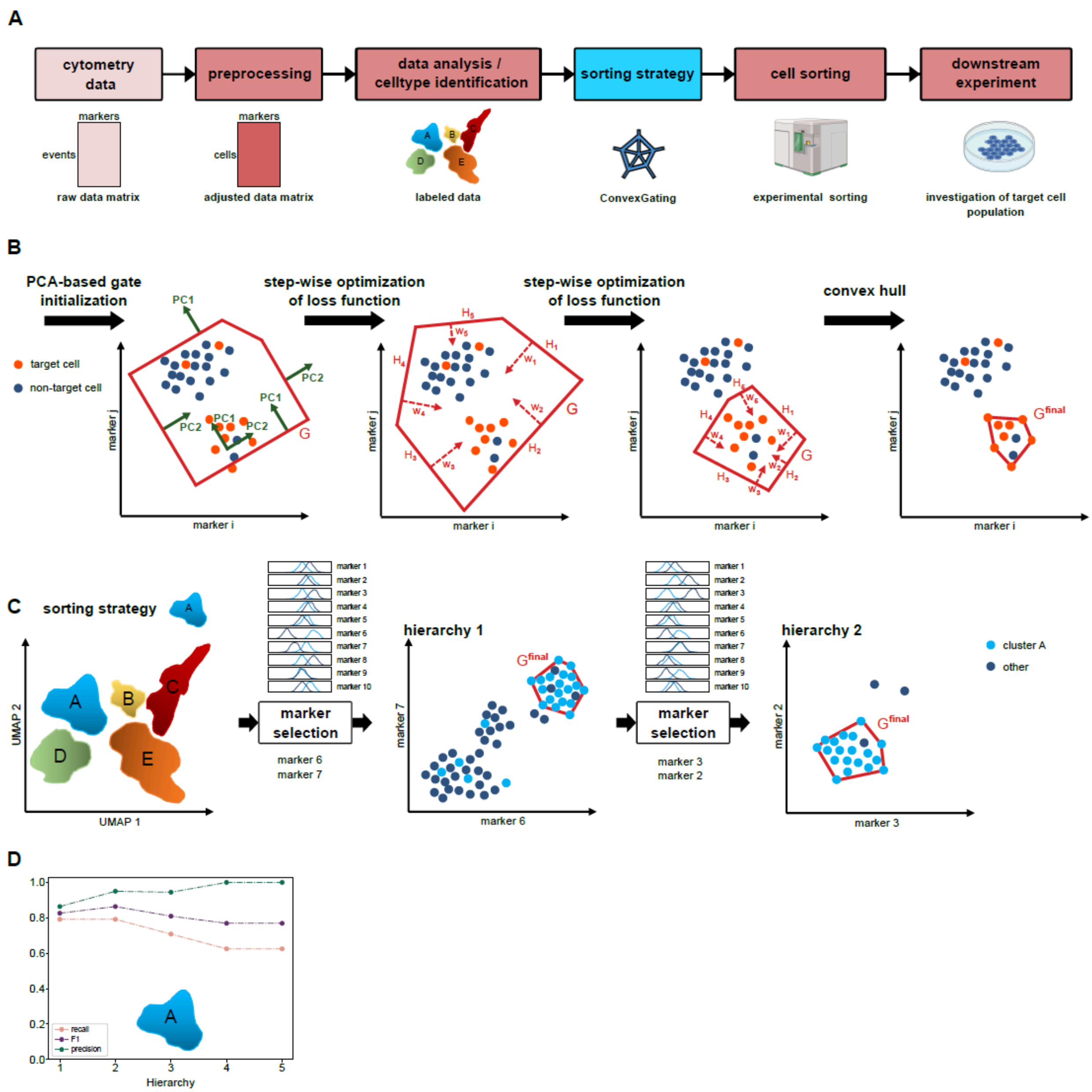

Figure 1. ConvexGating: a data-driven method for hierarchical and interpretable cell population gating. (**A**) Positioning of ConvexGating in a standard cytometry data analysis workflow. (**B**) Gate inference in 2D marker space. The gate is initialized partly at random and partly based on the first two principal components (PCs) derived from the target cell population, then refined through stochastic gradient descent (SGD)-based optimization of an explicitly designed loss function. The final gate corresponds to the convex hull of the SGD-optimized gate boundary. (**C**) Illustration of the algorithmic workflow for a sorting strategy targeting a cell subpopulation within a sample. At each hierarchy, marker selection is performed using a heuristic that quantifies the separability between the target and non-target cell populations. The

gate is then learned in the corresponding 2D marker space, followed by new marker selection and gate inference in the subsequent hierarchy. (**D**) ConvexGating is evaluated by recovery of target cells (recall), by contamination with other cell types (precision), and by the weighted mean of precision and recall (F1 score). Maximum F1 score determines the number of hierarchies in the final gating strategy.

Performance Variation Across Showcases: The reviewer correctly identifies that ConvexGating's performance varies across different contexts, which reflects its intended role as a practical tool rather than an optimal classifier. Importantly, ConvexGating's effectiveness depends on the quality of upstream clustering for cell type identification.

Our showcases demonstrate this context-dependency systematically. In Showcase 1, we establish that clustering appropriately identifies cell types and show that ConvexGating-inferred gates closely match manual gating strategies typically used for cell sorting. Showcase 2 extends this to high-dimensional flow cytometry data, where we compare the interrater variability in cell type definitions between manual gating and clustering approaches. Using clustering-defined cell types, ConvexGating successfully infers sorting-compatible gating strategies that would be impractical to define manually in high-dimensional space.

In Showcase 4, we directly acknowledge ConvexGating's performance limitations: while SVM achieves superior classification performance through high-dimensional flexibility, it cannot be translated to cell sorter operations. ConvexGating thus represents a deliberate trade-off, prioritizing sorting-compatibility over maximum classification accuracy.

Context-Dependent Strengths: ConvexGating's main strength lies in enabling data-driven cell sorting workflows while maintaining compatibility with existing FACS infrastructure. This strength is indeed context-dependent: it is most valuable when (1) clustering-based analysis is preferred over manual gating, (2) physical cell sorting is required, and (3) the performance trade-off is acceptable for the experimental goals.

We position ConvexGating not as universally superior to existing methods, but as a practical solution that enables previously impossible workflows by making clustering results actionable for cell sorting applications.

In the revised version of the manuscript, we highlighted the primary purpose and addressed this niche:

While using clustering to identify cell population has several advantages during data analysis, there is a considerable gap to transfer the cell identity back into a sorting strategy for further experiments. Such a translation of clusters to a sorting strategy requires reverse engineered gating strategies.

In the revised version of the manuscript, we clarified ConvexGating's positioning in the last paragraph of the introduction:

ConvexGating proposes gating strategies from clustering based cell type annotation consistent with manual gating and outperforms existing tools for reverse gate engineering.

We further revised the first paragraph of the results section to describe the workflow for ConvexGating:

Manual gating is an iterative process, where users select a set of markers to separate a population of interest from the remaining cells based on prior knowledge. When using clustering to identify cell types, there is a considerable gap to translate such a cell type definition to an actionable sorting strategy for further experiments. In our approach, ConvexGating, we mimic manual gating in an automatic, fully data-driven way to compute gating strategies for cell sorting from cluster-based cell type labels. The inferred gating strategy can then be applied in a cell sorting experiment, which effectively separates gating as an analysis tool from gating as a cell sorting technique (**Fig. 1 A**). Our approach is based on the assumption that every cell population in a flow or mass cytometry dataset can be identified by an appropriate clustering.

2. As a gating strategy, ConvexGating follows from cell clustering results, with cell populations already defined. Given there are many machine learning methods used to cluster cell populations, is the method very sensitive to the clustering methods being used and the clustering results?

We agree with the reviewer that a wide range of machine learning methods can be used for clustering cell populations. In principle, ConvexGating is agnostic to the clustering algorithm used. The strength of the algorithm is that it can be used independent of the initial machine learning clustering approach to infer a gating strategy solely based on the cluster identified. The accuracy and consistency of the gating output are therefore naturally influenced by the quality and stability of the clustering results, whose quality in turn depends on the thorough assessment of the person analysing the data as well.

To evaluate the sensitivity of ConvexGating to minor variations in input data (and by extension, clustering), we conducted a robustness analysis using KMeans clustering (**Reviewer Figure R1)**. Specifically, we clustered the data with KMeans and applied ConvexGating to generate gates. We then repeated this process across five independent runs, each time randomly removing 5% of the data after clustering. This simulates slight perturbations that may arise due to experimental variability or subsampling.

Our observations showed that:

- The same markers were consistently selected across all perturbations, sometimes with slight variations in the order of selection.

- The gated areas remained highly overlapping, as measured by Intersection-over-Union (IoU) scores, indicating spatial consistency in the gating results.

- Although ConvexGating did not produce identical results, it demonstrated a high degree of stability and robustness to small changes in the input data.

These findings support the conclusion that while ConvexGating depends on the chosen clustering method, it is relatively robust to small perturbations in the data that may influence clustering outcomes.

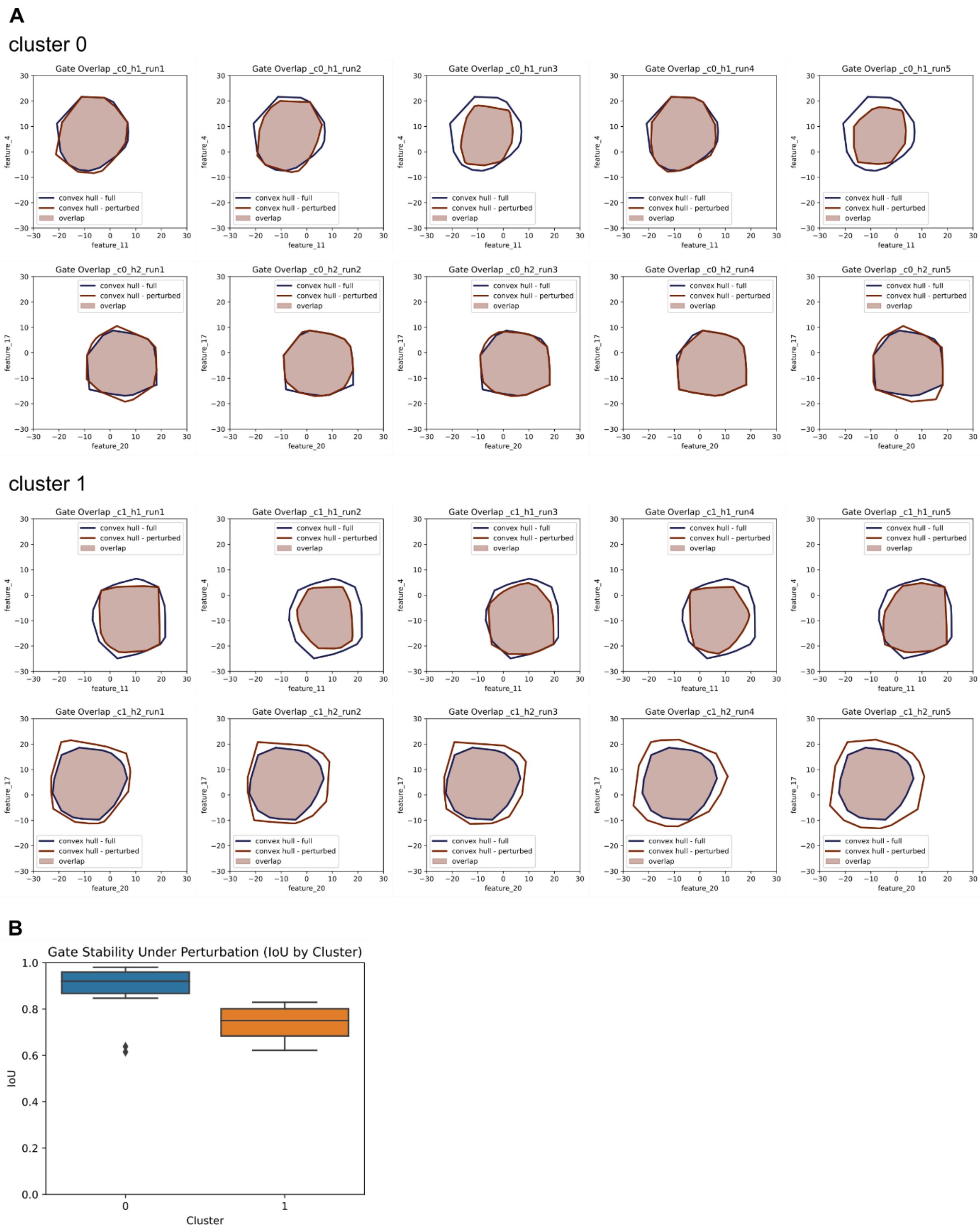

Reviewer Figure R1. Robustness analysis of ConvexGating under minor input perturbations. We generated a synthetic data set consisting of 5,000 cells and 20 markers, simulated with three underlying clusters using *make_blobs* from *sklearn.datasets*. We then applied *KMeans* clustering algorithm to group the cells into two clusters. ConvexGating was

applied to define a gating strategy for each cluster on the full dataset. To assess robustness to input perturbations, the gating was repeated five times, each time after randomly removing 5% of the data following clustering. **(A)** Gate overlaps between full and perturbed gating runs in the first gating hierarchy for cluster 0 (upper panel) and cluster 1 (lower panel). **(B)** Boxplot showing the Intersection-over-Union (IoU) of markers used across the entire gating strategy between full and perturbed gating runs.

3. How well the gating strategy (and the clustering as well) performs seems to be highly dependent on the marker gene selection. The authors mentioned in one showcase that ConvexGating supports optimal design of marker panels. ConvexGating selects marker first (the two markers with the highest heuristic value for the hierarchy) then learn the gate location. How sensitive is the method to the number of markers (or combinations) selected (is it only one pair with highest heuristic value picked, then how about joint effect of multiple marker pairs that can the separate the target vs non-target population distributions well)?

Thank you for this insightful question. We acknowledge that multiple marker pairs may jointly separate populations well; however, ConvexGating selects only the top-ranked marker pair at each hierarchy level based on our heuristic. The hierarchical structure of a gating strategy enables correlated or redundant markers to be selected in downstream branches, provided they remain informative for distinguishing target from non-target cells within the gated subpopulation. That said, it is also possible that only one of a set of correlated markers appears in the gating strategy if the relevant information is already captured earlier in the strategy. If a specific marker is expected or desired to appear in the gating strategy, users may optionally pre-filter correlated features to increase its likelihood of selection. Please also see our response to **Reviewer 2 Question 2** for a related discussion.

What's the difference between reduced vs full strategy in terms of the method (seems at each hierarchy you are not only restricted to one pair of markers)?

We thank you for raising this point and are happy to provide clarification. The difference between the reduced and full strategy lies in the depth of the gating process. Both strategies select a single marker pair per hierarchy level based on our heuristic. The reduced strategy applies gating only to the first two levels, while the full strategy recursively follows the entire learned gating hierarchy. This setup allows us to assess how much performance can be attributed to just the top two hierarchy levels (using only 4 markers in total) versus the full recursive process. We have revised the caption of **Supplementary Figure 7 B** to clarify the distinction between the full and reduced strategies:

Supplementary Figure 7. ConvexGating finds gating strategies for cell populations (four annotation levels, eight samples) in healthy human bone marrow. (**B**) Performance overview (F1, recall, precision) per cell type annotation level for the full strategies of up to five hierarchies (left panel) and for the reduced strategies of the first two hierarchies consisting of the initial two gating hierarchies (right panel) (boxes represent 25th percentile, median and 75th

percentile, respectively, whiskers show 1.5 * interquartile range and black diamonds represent outliers). Dots indicate the performance score for a specific cell type and sample.

Additionally, how does the method ensure the feature selection and gate locating well align at each hierarchy?

We thank the reviewer for raising this point. In ConvexGating, marker pairs are selected using a heuristic that compares the distributions of the target and non-target populations. A high heuristic score suggests that good separation is likely. Most importantly, this approach is computationally very efficient compared to alternative marker selection methods, such as using feature importance from classifiers. Once a marker pair is selected, the gate is learned directly in the corresponding 2D space, so gate location is naturally aligned with the selected features. As shown in the new **Supplementary Figure 1**, we further demonstrate that marker rankings produced by the heuristic strongly correlate with rankings obtained using SVM-based feature importance, indicating that the heuristic provides a reliable proxy for more computationally intensive methods while being substantially faster. While the heuristic may not be perfect, any intermediate hierarchy (marker combination) with limited separability of targets and non-targets is not problematic (though not ideal), as later levels in the hierarchy can introduce new, more informative markers that refine the gating.

4. In Fig S7, as the authors mentioned, the non-linear RBF SVM performed better than ConvexGating, basically this shows the boundary between cell populations is essentially non-linear. Then why the gating strategy has to use simple geometric shapes while knowing the true boundary is not simple? The 2D marker space is unique to ConvexGating, there could be variability between different cell populations that are not simply visible using two most discriminative markers.

Thank you for this important question. We agree that the true decision boundaries between cell populations are in general non-linear in high-dimensional space, especially when using clustering for cell population definition. ConvexGating does not attempt to directly model or recover this boundary in two dimensions. Instead, it translates this high-dimensional output into a hierarchical sequence of interpretable 2D gates to approximate the target population in a way that is compatible with real-world cell sorting platforms, which operate through recursive gating in two-dimensional marker plots.

While perfect recovery of the non-linear boundary in 2D is not feasible, ConvexGating aims to strike a balance between fidelity and physical realizability. This is essential for cell sorting, where cell isolation relies on executable gating strategies that can be implemented directly on cell sorters.

For demonstration purposes, we included comparisons with non-linear classifiers (RBF SVMs) to illustrate the performance upper bound in terms of classification accuracy. However, these models do not produce gating strategies that can be translated into physically executable instructions for cell sorting. ConvexGating aims to bridge this gap by providing interpretable,

hardware-compatible gating schemes, even if that comes at the cost of some classification precision.

In the revised version of the manuscript, we emphasized that SVM classifiers cannot be implemented directly on cell sorting devices in the benchmarking section:

"Notably, while SVM classifiers do not provide directly translatable gating strategies for experimental cell sorting, they provide a theoretical performance estimate for the separation of target cells."

5. Following the above question, in the Methods for the ConvexGating Algorithm, the way gate is defined is very similar to the way SVM defines the boundary, but more on a linear sense, if the authors could utilize the kernel function as in SVM and should be extensible to non-linear case, which is closer to real data scenario.

We agree with the reviewer that non-linear boundaries, such as those modeled with kernel functions in SVMs, often better reflect real biological data. While ConvexGating's gate definition is conceptually similar to linear SVMs, extending it with non-linear kernels (e.g., rbf) would not yield gating strategies that can be executed on physical cell sorters.

ConvexGating is designed to approximate complex, high-dimensional decision boundaries using interpretable, recursive 2D gates that are compatible with cell sorting on traditional flow cytometers. Although this limits expressiveness, it ensures practical applicability. Exploring more flexible but still executable gate shapes remains an interesting direction for future work once the corresponding machines allow for this feature.

6. In Methods section regularized loss, the authors include two problem specific regularization terms P1 and P2 (Supp Note 3) to tighten the gate, both terms look at distance between target population to the hyperplane (one for center, the other for all cells). In the objective function you minimize, you will penalize on larger P1 and P2, i.e. penalize larger distances, is this against a clustering or classification tuition where you wish to maximize the margin or enlarge the distance? Also, are there tradeoff between the two penalization terms? I thought they usually agree with each other.

We thank the reviewer for the thoughtful observation and for engaging closely with the regularization aspects of our method.

In contrast to traditional classification frameworks that aim to maximize margins between classes, ConvexGating is designed for cell sorting, where the objective is to tightly enclose a biologically meaningful target population within a specific region of feature space rather than achieve broad class separation. This is especially important when applying gates to novel data for cell sorting, where overly large gates that brute-force include outliers or capture regions devoid of target cells in the original sample are not desirable, as these regions might be a source of contamination.

To this end, we introduce the two regularization terms, P1 and P2, that penalize hyperplanes for drifting too far from the target population. P1 penalizes distance from the centroid of the target population, encouraging hyperplanes to stay aligned with the overall center of mass. P2 encourages the hyperplanes near the bulk of the target data, offering robustness against outliers. While these penalties often agree, they capture slightly complementary aspects. During tool development, we found that using both together consistently performed better than using no regularization or only one of the penalty terms.

In the revised version of the manuscript, we added the following explanation of the two penalty terms in the methods section:

Intuitively, P1 penalizes distance from the centroid of the target population, encouraging hyperplanes to stay aligned with the overall center of mass. P2 encourages the hyperplanes near the bulk of the target data, offering robustness against outliers. While these penalties often agree, they capture slightly complementary aspects, such that using both together consistently performed better than using no regularization or only one of the penalty terms.

Reviewer #2 (Remarks to the Author):

This article by Friedrich et al., describe ConvexGating, a new algorithm implemented in a Python R package to derive gating strategies targeting user-input cell populations (e.g. cell clusters). The proposed method relies on the heuristic selection of K pairs of markers (designated as a “hierarchy” in the article) based on expression quantiles computed on the target cell population and the rest of the cells. For each hierarchy, a set of K hyperplanes is then optimized by stochastic gradient descent, optimizing for a weighted cross-entropy loss function and with regularization terms. Finally, a convex hull is applied to further adjust the convex polygon to target cells, and the process is repeated for the next level of the hierarchy. The authors demonstrate the use of ConvexGating on a number of datasets covering a wide range of technologies (flow cytometry, mass cytometry, CITE-seq, spectral flow cytometry).
In my opinion, this type of research is useful to make experimental design in flow cytometry more data-driven. There are however a number of limitations in this study, notably the performance of the method is possibly overstated. Consistently testing the gating strategies on held-out data and independent samples appear important as gating strategies that would not generalize would have little practical utility.

Major points:

1.All results of the ConvexGating algorithm are reported on the sample on which the algorithm was ran. This carries a risk of overfitting. The authors need to show cross-sample performance (training on a sample and testing on another), as well as train/test split performance on single samples. This seems relevant since the choice of markers for the different levels of the hierarchy varies substantially across samples, for instance in supplementary fig 3A.

We thank the reviewer for their critical assessment of the algorithm. To address the concern of overfitting, we conceptualize several types of generalization. The first one is generalization on single samples (e.g. within a donor sample) via train-test split. The second one is cross-sample performance with training on one sample and testing on another. In the context of machine learning, this represents a validation set. Even more generalizable is cross-sample performance with independent samples, i.e. when we can exclude information leakage due to joint cell type annotation. The last scenario represents a validation setup that is closest to a real-world application.

To address the generalization on single samples, we modified the method comparison benchmark with a train/test split setup on a single sample as suggested by the reviewer (see **major comment 3** of this reviewer for details). Briefly, ConvexGating created more precise gates than Hypergate on the test sets in the majority of runs, see new **Supplementary Figure 8** and **Reviewer Figure R3**.

To address cross-sample performance, we extended the functionality of ConvexGating to transfer gating strategies and used it to assess cross-sample performance on all cell types and respective subsets in the small DC panel (see also **major comment 4** of this reviewer for

details), i.e. the gate learned on one donor sample was transferred to the other two donor samples and we assessed the performance (see new **Supplementary Figure 5**). In almost all cases, the transferred gates allow for high precision. This experiment shows that transferring the gate to a different sample results in high precision sorting of the target population, i.e. low cross-contamination, even though the marker choice differed in different levels of the hierarchy. This result shows that the algorithm learns to robustly separate the target cell type from all other cells.

In the revised version of the manuscript, we referred to the gate transfer experiment as follows:

“As optimized for highest precision, over all populations, our computational gating strategy has very high precision scores and for most cell types also high F1 scores (**Fig. 2 F** and **Supplementary Fig. 4 D**), and performance is stable when gating strategies are applied to unseen samples (**Supplementary Fig. 5**).”

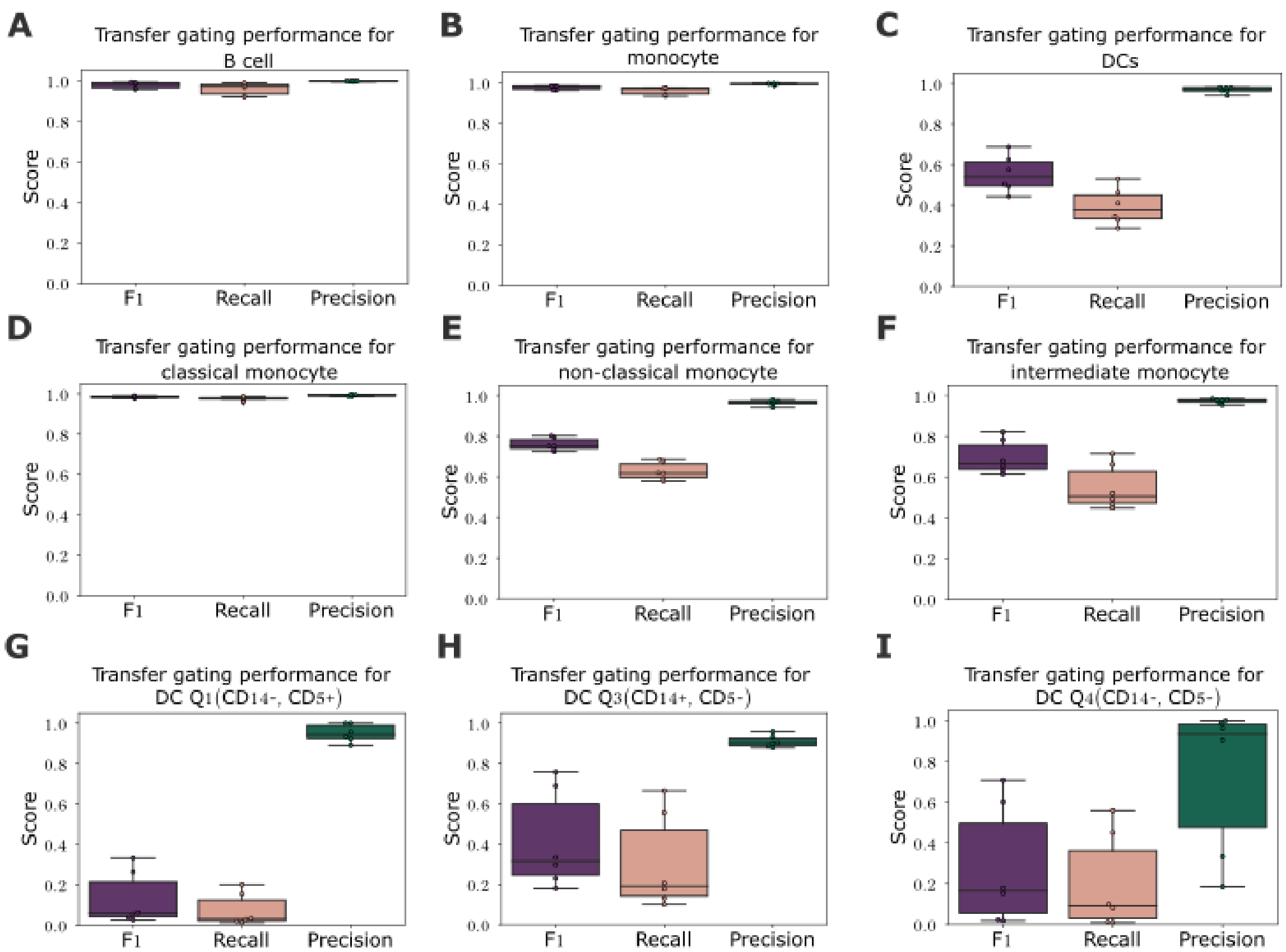

Supplementary Figure 5. Performance of gate transfer to the two other samples in the small DC panel for all cell types and subsets. Performance metrics for broad cell type annotation of B cells **(A)**, monocytes **(B)** and DCs **(C)**. Performance metrics subset annotation (cell type level 2) of classical monocytes **(D)**, non-classical monocytes **(E)**, intermediate monocytes **(F)**, DC Q1 (CD14-, CD5+) **(G)**, DC Q3 (CD14+, CD5-) **(H)**, and DC Q4 (CD14-, CD5-) **(I)**.

Please note that in order to demonstrate the generalizability of the gating strategies of ConvexGating to unseen samples, we applied the inferred gating strategy to sort for CD8+ TEMRA T cells in the validation experiment, which demonstrated the utility of the tool on a completely new sample, where we validated the cell identity with single-cell RNAseq (see paragraph "ConvexGating-sorted CD8+ TEMRAs show higher purity than conventionally sorted cells" with **Figure 4** and **Supplementary Figure 9**).
We further added a new set of experiments for an additional example from mouse white adipose tissue, where we validated the cell identity of two progenitor populations with single-cell RNAseq (see new paragraph "ConvexGating-sorted progenitor cells of white adipose tissue show higher purity than conventionally sorted cells" with new **Supplementary Figures 10-11**).

2.In many instances, it seems that markers are selected (with the heuristic "step 1" described in the method) but end up having little influence on the final output. For instance, when gating on pDCs in fig 3h, the authors report that ConvexGating identified CD57-CD123+CD13-CD45RA+ as the phenotype of pDCs. But when examining Fig 3H, it appears that CD57- has no effect to gate on pDCs since CD123+ cells are all CD57- ? Worryingly, the contribution of CD57 appears to be mostly to gate out CD123+ events that are below the axis for CD57 intensity. This suggests that CD57 does not meaningfully, but rather artifactually, contribute to the identification of pDCs in this instance.

We thank the reviewer for this thoughtful observation regarding marker selection in the pDC gating example (**Figure 3 H** and **Reviewer Figure R2 A**). This provides an excellent opportunity to demonstrate ConvexGating's flexibility and to highlight how systematic marker exploration can further refine algorithmically inferred gating strategies.
To investigate how marker choice shapes gating performance, we examined the feature distribution for each marker for pDCs versus non-target cells (**Reviewer Figure R2 B**) and conducted a series of runs with ConvexGating in which markers were successively excluded. This analysis illustrates how users can actively guide the tool toward biologically parsimonious solutions.
In the first experiment, excluding CD57 led ConvexGating to select CD3- CD123+ cells in the first hierarchy and CD4+ CD45RA+ cells in the second hierarchy (**Reviewer Figure R2 C**), achieving precision 1.000, recall 0.810, and F1 0.895. Excluding both CD57 and CD3 further improved performance: ConvexGating identified CD11c- CD123+ cells in the first hierarchy and CD4+ CD45RA+ in the second (**Reviewer Figure R2 D**), yielding precision 1.000, recall 0.865, and F1 0.928. Three out of the four markers — CD11c, CD123, and CD45RA — are well-established identifiers of human pDCs, with CD11c serving as a classical marker distinguishing conventional DC subsets[1]. In a third experiment, excluding CD57, CD3, and CD11c together produced a streamlined one-step hierarchy selecting CD123+ CD45RA+ cells, with precision 0.980, recall 0.920, and F1 0.949 (**Reviewer Figure R2 E**). As this configuration already relied exclusively on positive markers and precision began to decline with further exclusions (**Reviewer Figure R2 F**), we did not pursue additional iterations.
Taken together, these results demonstrate that ConvexGating can achieve strong pDC identification across multiple marker configurations, and that iterative, user-guided marker

selection is a powerful way to balance computational performance with biological interpretability. The analysis confirms that negative markers such as CD3 and CD11c can contribute meaningfully to precision, while the positive-marker-only strategy in a one-step hierarchy retains excellent, yet slightly lower performance. This interactive refinement workflow — in which users leverage ConvexGating's output as a starting point and apply domain knowledge to improve parsimony — represents a key strength of the tool.

Looking ahead, incorporating co-correlation structure and biological relevance scoring alongside geometric utility metrics is an exciting direction for future development that would further enhance the tool's ability to favor biologically canonical markers automatically.

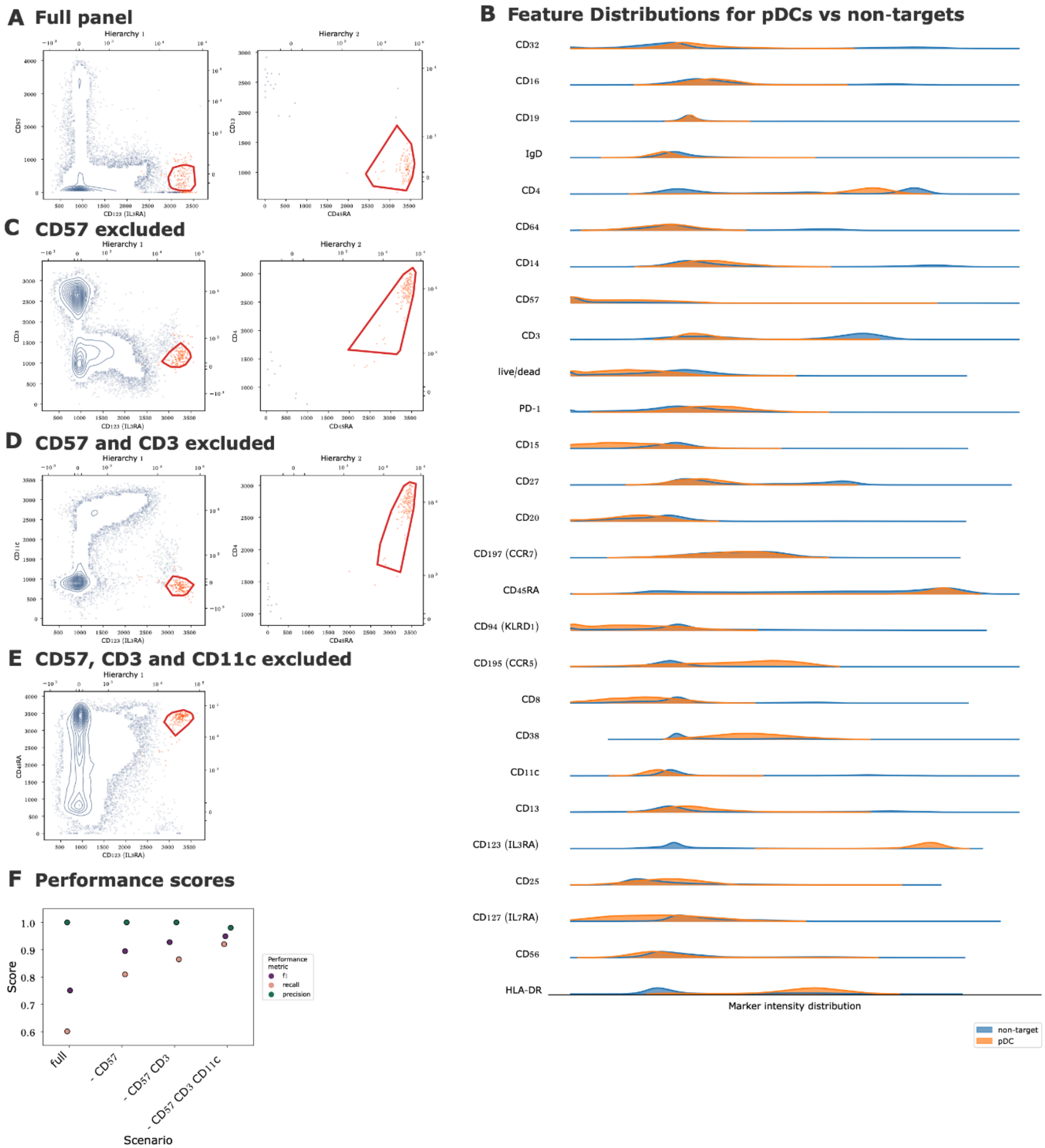

Reviewer Figure R2. Changes in performance and marker selection for pDCs upon exclusion of selected markers. (A, B-E) Inferred gating strategy on full marker panel **(A)**, excluding CD57 **(C)**, excluding CD57 and CD3 **(D)**, and excluding CD57, CD3 and CD11c **(E)**. **(B)** Feature distribution for pDCs (orange) and non-target cells (blue) for all markers in the panel. **(F)** Performance scores showing F1, recall and precision for all scenarios.

3.All the comparisons to previous methods such as Hypergate should be done with each method trained on a random subsample of the data and evaluated on left-out events to account for potential overfitting. Additionally, the authors report to have subsetted 2,000 positive events

and 2,000 negative events before running Hypergate, and state that this is the recommended procedure. I do not think this is the recommended procedure nor that this is a fair comparison. The two methods should be ran and evaluated on the exact same data.

We thank the reviewer for their critical assessment of our comparison methodology.

Regarding subsampling for Hypergate: The reviewer suggests that subsampling is not the recommended procedure for Hypergate. We respectfully disagree, as the original Hypergate publication (Becht et al., Bioinformatics 2019) explicitly describes this approach as follows:

"Binary classification: From each dataset and for each cell population manually gated by the authors', we sampled 1000 events if the population was larger than 2000 events, or half of the events otherwise. We also sampled a corresponding proportion of negative events."

In addition, the authors of Hypergate explicitly discuss the question of data input size in the github vignette of the tool (https://github.com/ebecht/hypergate#how-big-can-the-input-matrix-be), where they state that the size of the input is essentially limited by the memory of your system. Our use of 2,000 positive and 2,000 negative events follows this established methodology as outlined in the original publication (Becht et al., Bioinformatics 2019). This subsampling addresses Hypergate's computational limitations and ensures balanced training data, which is critical for the algorithm's performance. Running Hypergate on the full, imbalanced dataset would deviate from its validated methodology and likely result in suboptimal performance that unfairly disadvantages the method. We modified our benchmark to use a maximum total of 50,000 cells for training to balance runtime vs appropriate representation of the data with a detailed description in the next section.

Regarding train/test splits: We modified the performance test to incorporate train/test splits of the data and evaluated the performance in the exact same data in all methods. In order to address the different subsampling recommendations of Hypergate vs ConvexGating, we created training datasets with a fixed ratio of target/non-target cells, while the remaining data were used as test dataset. The training data contained 70% of the target cells. We used a maximum subset of 50,000 cells for training, and we used fewer cells for training, if 70% of the total amount of target cells were less than the specified ratio of target to non-target cells, in order to maintain the ratio. To be in accordance with the original Hypergate publication, we used a ratio of 1:1 of target to non-target cells.

We revised **Supplementary Figure 8** to display the new benchmark results and specifically highlighted the comparison of Hypergate vs. ConvexGating in **Reviewer Figure R3**. ConvexGating outperformed Hypergate across all annotation levels and tested scenarios in terms of mean F1 score and precision.

In the revised version of the manuscript, we incorporated the new benchmark results in the section "ConvexGating strategies show better applicability across multiple scenarios compared to Hypergate":

"We benchmarked ConvexGating, the gating tool Hypergate[27] and off-the-shelf SVM classifiers on three test scenarios (see **methods** and **Supplementary Note 2**). Notably, while SVM classifiers do not provide directly translatable gating strategies for experimental cell sorting, they provide a theoretical performance estimate for the separation of target cells. ConvexGating showed higher mean F1 scores on the test set compared to Hypergate across all annotation levels and tested scenarios (**Supplementary Fig. 8**). ConvexGating focused on high precision while Hypergate prioritized high recall. Compared to SVM classifiers, we found that ConvexGating outperformed the linear SVM classifier on the DC panel and annotation level 2 of the large PBMC panel, while the linear SVM achieved higher mean F1 score at annotation level 1 of the large PBMC and the cyTOF panels. ConvexGating also achieved higher mean F1 score than the non-linear rbf SVM at annotation level 2 of the DC panel; however, the rbf SVM showed the highest mean F1 scores in all other tested scenarios (**Supplementary Fig. 8**). The rbf SVM employs a non-linear kernel transformation to project the data into a high-dimensional space where it creates a decision boundary separating the target from the non-target population. However, this boundary cannot be directly translated into a hierarchical gating strategy using simple geometric shapes such as polygons in two-dimensional marker space, which is essential for cell sorting. In contrast, ConvexGating is designed to provide gating strategies that are directly applicable to cell sorting experiments. This practical applicability comes at the expense of a certain degree of separation power compared to more complex classifiers such as the rbf SVM."

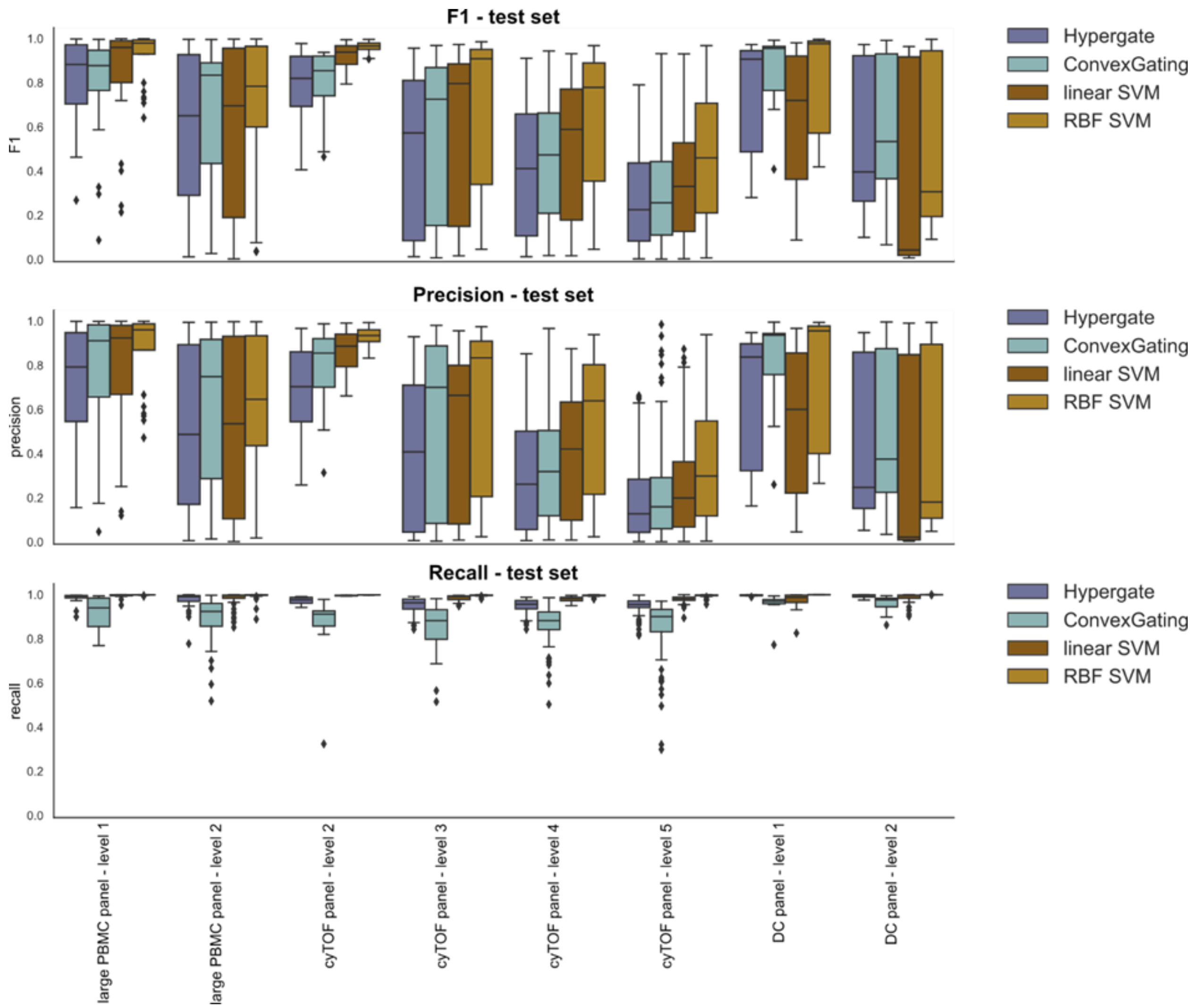

Supplementary Figure 8. Computational benchmark analysis comparing gating strategies from ConvexGating and Hypergate with machine learning classifiers. F1 score (upper panel), precision (middle panel), and recall (lower panel) boxplot results on the test set for gating strategies learned by ConvexGating and Hypergate, and for classification performance of linear SVM and RBF SVM models. Boxes represent the 25th percentile, median, and 75th percentile, respectively; whiskers indicate 1.5× the interquartile range, and black diamonds denote outliers. The benchmark analysis includes the large PBMC panel with two cell type annotation levels, the human bone marrow cyTOF panel with four annotation levels, and the DC panel with two annotation levels.

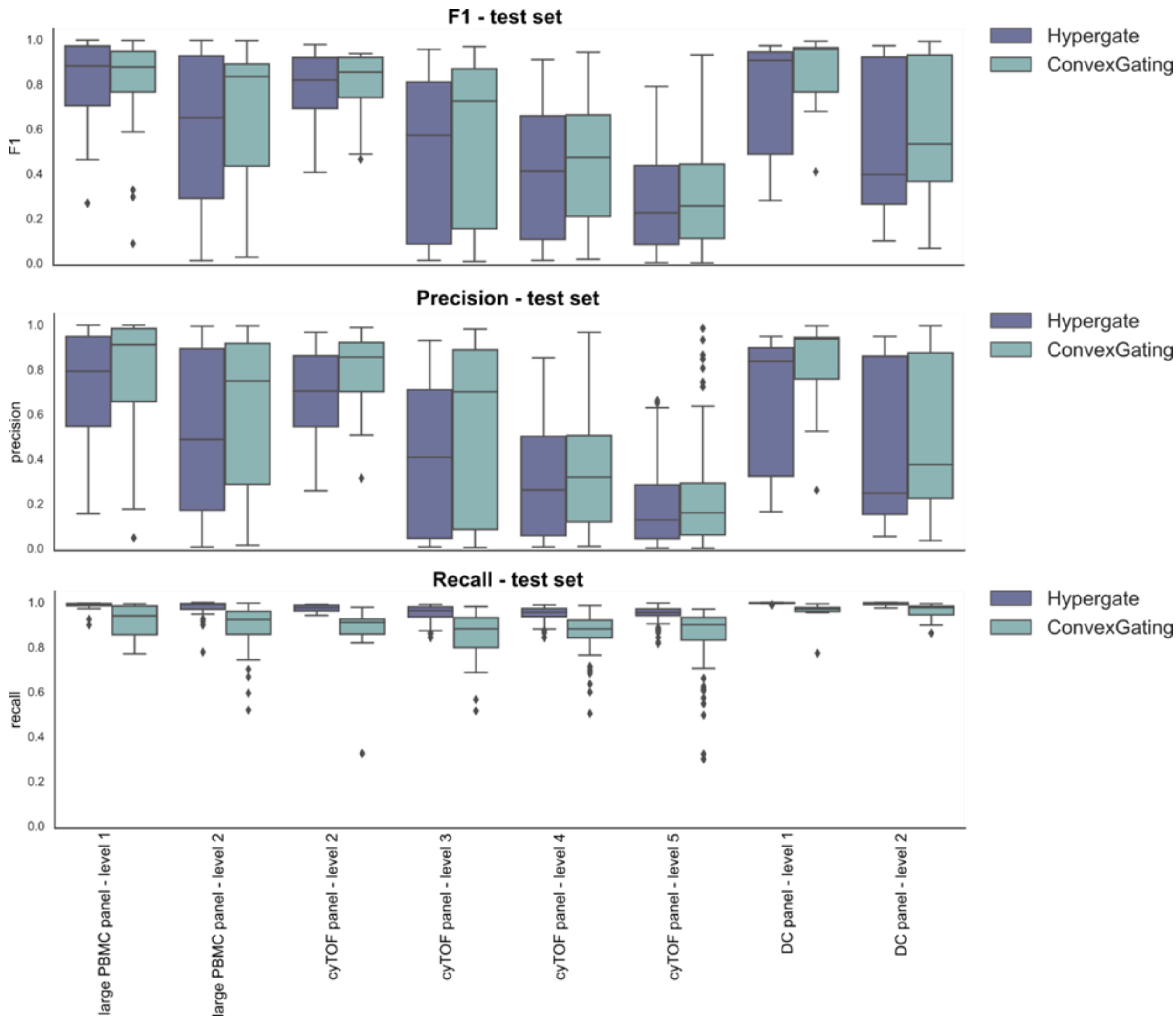

Reviewer Figure R3. Benchmark Hypergate vs. ConvexGating. Boxplots showing F1 score (upper panel), precision (middle panel), and recall (lower panel) for sorted cell populations in the test set, obtained using gating strategies derived from either Hypergate or ConvexGating on the training set. Results are shown for the large PBMC, cyTOF, and DC panels across different annotation levels.

4.In supplementary fig 3E/F/G, the authors show the output of ConvexGating for dendritic cells subsets. The first step involves a very tiny convex polygon drawn on CD1c+HLA-DR+ cells, resulting in very high precision and low recall. Can the authors show that the learnt gating strategies for DC subsets actually apply to the other samples ?

We appreciate the reviewer's interest in the inferred gates for the DC subsets. To address the concern of transferability of the gates, we implemented a gate transfer function, which applies the inferred gate from one sample to another. Please note that the gate transfer function takes

the data “as is” without any batch correction, which clearly limits its applicability across time, devices or labs.

We specifically applied the inferred gating strategy for the cell types DC Q1 (CD14+ CD5-), DC Q3 (CD14- CD5+) and DC Q4 (CD14- CD5-), which were learnt on one sample, and applied it to all cells of each of the other two samples (i.e. gate inferred from sample KR was transferred to samples KW and MF, respectively), and computed the performance metrics F1, recall and precision (see **Supplementary Figure 5 G-I**). We observe that the precision of the transferred gates is in the range of 0.9 - 1.0 in all cases with two exceptions (see **Supplementary Figure 5 I**). The outliers in DC subset DC Q4 (CD14- CD5-) were caused by gating strategy inferred from sample MF. This could be due to a particularly low F1 and recall for this case (see **Supplementary Figure 4 G**). In all other cases, the gate transfer yielded high precision gates even though the gates in the first hierarchy were comparatively small. The overall high precision is in line with the goal of ConvexGating to inform cell sorting experiments. We believe that the gate transfer function is a useful complement to assess the generalizability of the inferred gating strategies.

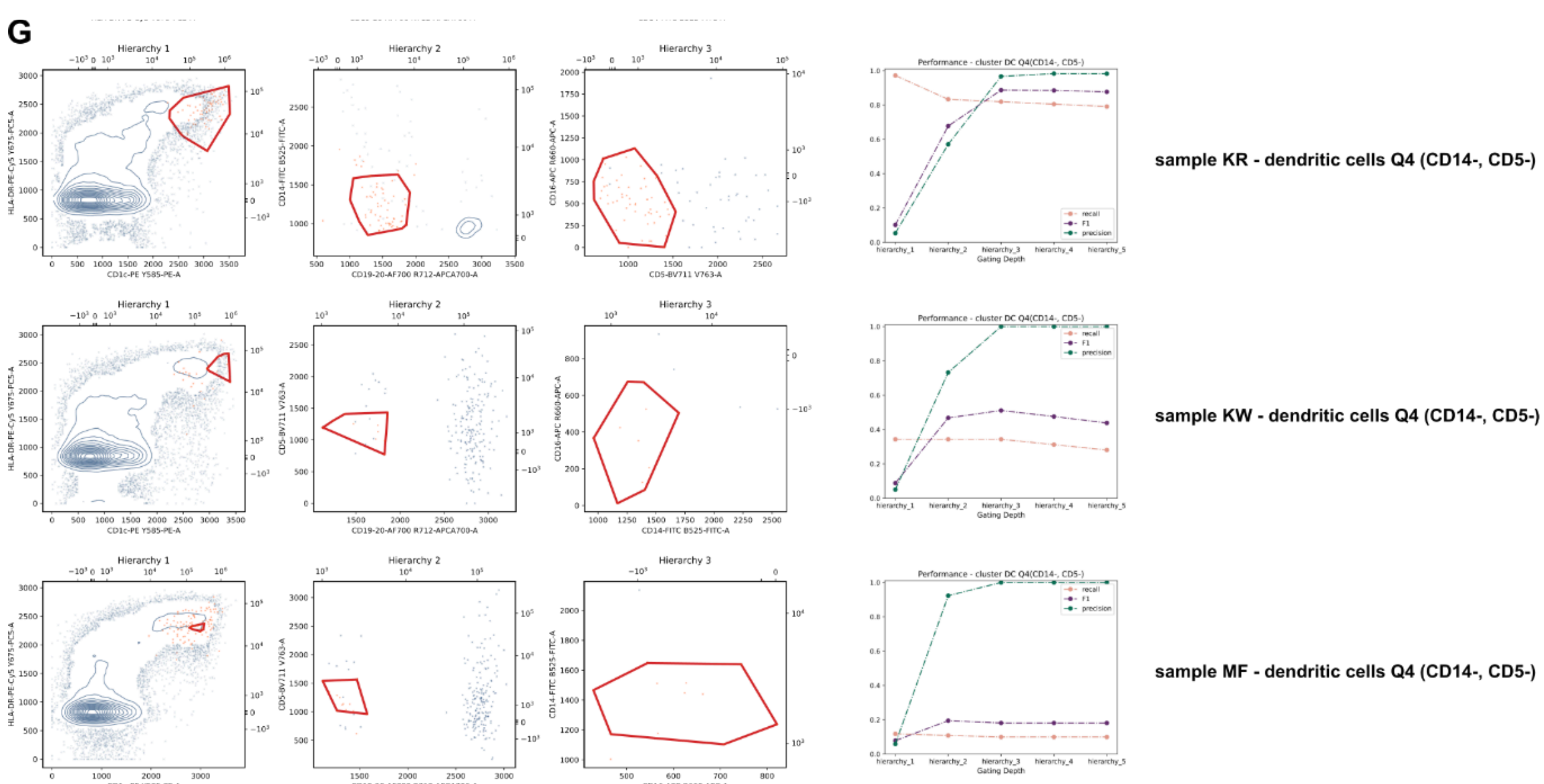

Supplementary Figure 4. (**G**) Inferred gating strategies for the DC panel for cell type annotation level 2, dendritic cells Q4 (CD14-, CD5-).

5.The authors use ConvexGating to define a gating strategy for Temra and Tnaive cells, and compare it for sorting to a manual gating strategy. This manual gating strategy is however not shown. The methods mention “canonical markers” but these are not explicitly mentioned.

We appreciate the reviewer’s point on the manual gating strategy. The conventional gating strategy uses FSC/SSC to remove doublet events, then selects CD19- and CD3+ cells to remove B cells and select T cells. Naive T cells are selected via CCR7+ CD45RA+ gate while Temra are selected via CCR7- CD45RA+ gate. This strategy is in accordance with Muroyama et

al.[2] and Verma et al.[3]. We included the gating strategy for the conventional gating strategy in **Supplementary Figure 9 D**:

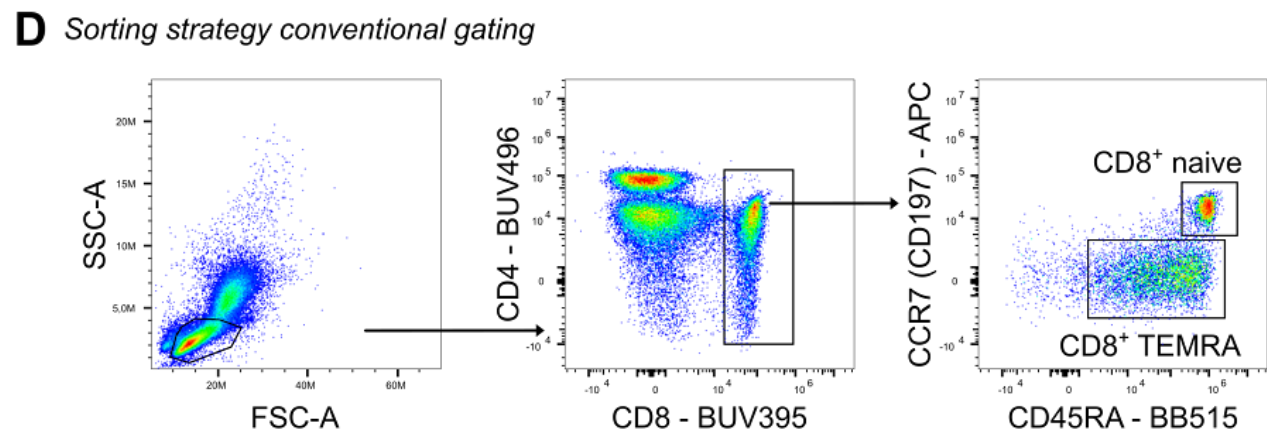

Supplementary Figure 9. (**D**) Conventional sorting strategy for CD8+ naive cells and CD8+ TEMRA cells (both shown in the last scatter plot).

6.After sorting Temra and Tnaive cells, the authors report the average protein expression of the 5 identified cell transcriptomic clusters based on the index sort data (Sup fig 8d). The data is scaled column-wise which does not allow interpreting the raw expression data. Worryingly, many markers appear “differentially-expressed” on this heatmap across Temra and Tnaive cells that should not be expressed in these cell population (e.g. CD56). This could be due to the scaling, but may highlight compensation issues. In particular, the CD8 - ‘BUC395‘ versus CD27 - BUV805 plot shown in fig S8A has CD27+ CD8- events that are leaning towards the negative, which suggests overcompensation.

We thank the reviewer for their astute observation. We thoroughly revisited and adjusted the compensation matrix and provided a high-resolution image of the pairwise spillover matrix as PDF with bi-exponential scaling as attachment to this response.

We agree that only showing a scaled heatmap could be misleading, therefore, we included an unscaled heatmap for both populations (**Supplementary Figure 9 H**):

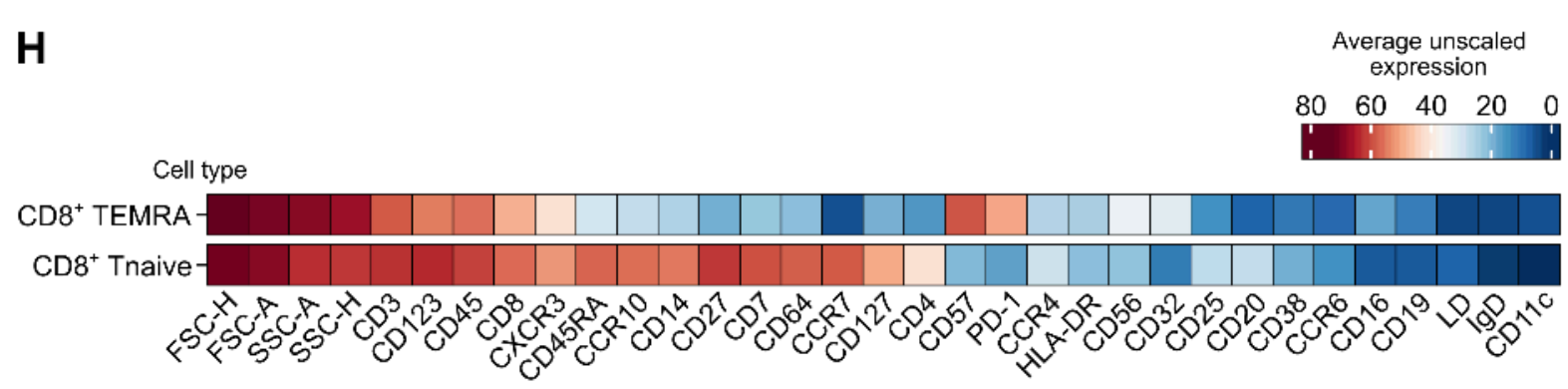

Supplementary Figure 9. (**H**) Overview of average unscaled marker protein expression across clusters.

7.The last panel of figure S8A (CD3-APC-H7 versus CD16-BUV615) features a blurry background which abruptly stops horizontally, and absolutely no event outside of the second gate. I am therefore confused about what is being shown. Has the image been edited in some way ? Why is this gate used although no events are being excluded ?

We sincerely thank the reviewer for their thorough and thoughtful examination of the panel figures in **Supplementary Figure 8 A.** We inspected the image in question and realized that the image quality had diminished due to file conversions. We revised the figure and provided a higher resolution image in the revised version of the manuscript (**Supplementary Figure 9 C**).

The reviewer correctly notes that the final gating step shown in the image contained no events. This gate was intentionally included as a quality control measure to ensure no contamination from other cell types would occur when transferring the gating strategy to the sorter. The figure demonstrates how the predicted gates are applied to the actual data.

To address this concern and improve clarity, we have now included precision and recall metrics for the ConvexGating run alongside the gate output results (**Figure 4 B**). These metrics demonstrate that the penultimate gating steps already achieve very high precision and recall. Thus, the additional final gate does not adversely affect performance and serves as an extra safeguard to maximize precision during cell sorting and ensures transferability to a previously unseen sample.

8.2D dotplots are shown with solid colors (e.g. fig 2d, 2h, 3b) or as contour plots with no outliers (e.g. fig 5). This contradicts a statement made in the nature portfolio reporting summary checklist (“All plots are contour plots with outliers or pseudocolor plots”).

We thank the reviewer for their concern on the contradictions with the reporting summary.
In our manuscript, we mostly use clustering to analyse the flow/mass cytometry and CITEseq data with pytometry and scanpy, such that the analysis plots come in the form of heatmaps and UMAPs.
We understand that the concern addresses the output plots of ConvexGating. These plots resemble the analysis plots of a FlowJo analysis. However, ConvexGating can not be seen as an analysis tool, but a prediction method for downstream experiments (see **Figure 1 A**). The output of the tool are gate inference plots, when the data analysis is already completed. We therefore consider it unwise to give the gate inference plots the same appearance as data analysis plots. As a consequence, the output format does not 100% reflect the Nature Portfolio Reporting Summary for flow cytometry data and therefore the guidelines do not apply for the gate inference plots of ConvexGating. To reduce the overplotting issue of the scatter plots created by ConvexGating, we post-processed all plots to display highly dense regions as contours and outliers as individual points.
To illustrate the difference of the FlowJo analysis plots and gate inference plots from ConvexGating, we display plots from both tools in the case of the CD8+ TEMRA T cells of our validation experiment (see **Figure 4 B** for ConvexGating plots and **Supplementary Figure 9 C-D** for FlowJo analysis plots).
Please note that the contour plots in **Figure 5** were derived from cyTOF data, where the Nature Portfolio Reporting Summary does provide a specification of presenting this data type.

In accordance with the Nature Portfolio Reporting Summary, we adjusted all plots where flow data was analysed with FlowJo according to the requirements (see **updated Supplementary Figure 1** and **updated Supplementary Figure 9 C-D**).
We further added the following description to the methods section:

Contour-based density visualization

For visualization of the derived gating strategies, density plots were generated for each applicable hierarchy level using 2D histograms of target and non-target populations in the corresponding 2D marker space, followed by Gaussian smoothing to obtain continuous density estimates. Contour levels were adaptively selected based on population size, enabling clear visualization of target and non-target distributions in large flow-cytometry datasets.

9.The plots shown are in transformed scale using biexponential transformations. Original scale tickmarks are not present. The biexponential plots have a number of free parameters that are not reported, so it becomes difficult to evaluate the data shown. The checklist states “The axis scales are clearly visible” which although ambiguous suggests that decades for raw intensities should be reported ?

We thank the reviewer for their remark on the axis scaling of the gate inference plots of ConvexGating. We understand that the normalized values are difficult to compare to original scale values. We designed ConvexGating as a flexible tool to infer gating strategies from flow and mass cytometry data as well as CITEseq data. The pre-processing of these different data types requires different transformations for normalization. For instance, while we used the bi-exponential transformation for flow cytometry data, we used the arcsinh transformation for cyTOF data as implemented in the pytometry package, not the ConvexGating package. Moreover, one may use different parameters for the biexponential scaling depending on the fluorophor and marker properties in high-dimensional flow cytometry. The normalization procedure puts all data to the same scale, which is essential for the high-dimensional analysis and machine learning applications. ConvexGating takes the normalized input data at face value regardless of the pre-processing steps. To facilitate the interpretability of the output plots, we added a secondary scale with the original scale of the data on the top and right side of each scatter plot of the ConvexGating workflow.

We further added the parameters used for the bi-exponential transformation to the method section in the revised version of the manuscript.

Minor points:

1.While it is not the main focus of the paper, there are many statements that advocate for clustering-based phenotyping over manual gating. While the authors advocate for clustering, a fair acknowledgements of manual gating advantages over clustering should also be stated (e.g. that the phenotype is informed by previous knowledge and generates interpretable cell labels)

We thank the reviewer for pointing out the advantage of interpretability of manual gating. We added the following statement in the second paragraph of the introduction:
“This iterative approach builds on prior knowledge and yields a straightforward cell type definition.”

2.Fig 1B puts a lot of emphasis on the convex hull but the core of the method seems to be the stochastic gradient descent step

We re-designed **Figures 1 B** and **1 C** to improve clarity of the algorithmic steps.

3.In Fig 3C, the authors compare manual labels generated by multiple independently-generated manual gating strategies to published labels with normalized mutual information. It is not clear what the benefit of NMI is compared to accuracy in this setting ?

We thank the reviewer for their critical assessment of our methodology. Normalized mutual information (NMI) is advantageous over accuracy for the following reasons.
Firstly, it handles label mismatches, as NMI does not require exact label correspondence between two annotations. For instance, if one rater labels cells as "T cells" and another one as "T lymphocytes", accuracy would score this as incorrect despite referring to the same cell type, while NMI can still capture the underlying concordance. We ensured that name variations still referred to the same cell type. Secondly, it manages missing classes. For instance, we included the debris label class in the comparison to the Gold standard, because the inter-rater variability leads to slight variations which events are considered valid and therefore subject to cell type annotation. When some cell types are present in only one annotation, accuracy becomes problematic as one cannot calculate meaningful accuracy for unmatched classes. NMI handles this naturally by measuring information content rather than requiring one-to-one label mapping. This was particularly important as some raters did not annotate all cell types in the benchmark. Thirdly, NMI has an information-theoretic foundation. NMI quantifies how much knowing one annotation reduces uncertainty about the other, which is exactly how we aimed to assess concordance between different annotation schemes.

4.Figure 4 is described after figure 5 in the text

We thoroughly reviewed the order of the figures in the text. All figures are labeled in order of appearance. However, we noticed that **Supplementary Figure 5** is referenced in the section before **Figure 4**, which might have led to the confusion. We revisited and updated the enumeration to accommodate new Supplementary Figures and ensured that Figures and Supplementary Figures are labeled in order of appearance independent of another.

5.Line 482 : typographical error, break instead of brake

We checked the lines in question and could not find the mentioned typographical error.

6.Line 592 : typographical error (104 instead of 10,000)

We corrected the formatting error, which should have been 10^4, and replaced it with 10,000.

7.In the methods, the authors mention bi-exponential transformations but the coefficients used is not clear

We thank the editor for their remark. We added the coefficients used for the bi-exponential transformations to the methods section.

8.The authors advocate for clustering-based phenotyping as a more unbiased strategy, but the methods describe the use of a “split and merge” strategy. Doesn’t this step introduce arbitrariness in the analysis ?

We thank the reviewer for this important clarifying question about the apparent contradiction between advocating for unbiased strategies while we modified the clustering approach as supervised split-and-merge strategy. We argue this represents informed biological interpretation rather than arbitrary decision-making. Clustering provides an objective foundation based on high-dimensional data structure, yet tends to overcluster the data. Rare cell populations like DCs require overclustering to be detected, while abundant populations like T cells get subdivided. This is a common observation and recognized challenge especially when analysing single-cell RNAseq data and there are a number of statistical approaches to address the issue of overclustering[4–6]. The split-and-merge step allows users to make biologically-informed decisions about which computational clusters represent meaningful cell types - a necessary step given the inherent challenge, where we have to account for biological variation, difference in abundance and potentially continuous marker distributions. While this introduces some subjectivity, starting from data-driven clustering results and making explicit merge/split decisions provides a transparent and reproducible framework for cell type definition.

9.I do not understand the equation stated in line 752 : q_f are length-3 vectors, so I am not sure how to interpret the modulus. Are the authors computing the L1 norm ?

We thank the reviewer for pointing this out, as the definition was not sufficiently clear in the previous version. The modulus represents the scaled L2 norm of the difference between the percentile vectors, equivalently corresponding to one half of their mean squared error. We updated the notation in the manuscript to avoid ambiguity.

Reviewer #2 (Remarks on code availability):

I've installed the package and followed the tutorial, which illustrates the use of convexgating on 5000 simulated cells.

A tutorial on real data starting from raw data and showing all the upstream steps (e.g. compensation, transformation, clustering of the data) and application of convexgating would be useful.

We thank the reviewer for their suggestions on the addition of tutorials. We would like to point out that the pre-processing steps were performed with pytometry/scanpy tools from the scverse ecosystem. To facilitate reproducibility and provide a practical reference for upstream steps and ConvexGating application, we have uploaded all data preprocessing and annotation notebooks to our reproducibility GitHub repository: https://github.com/buettnerlab/reproducibility_convex_gating.

Reviewer #3 (Remarks to the Author):

Friedrich et al present ConvexGating, a method for learning gating strategies from cytometry data using convex shapes in 2D marker space. The approach is designed to mimic (and improve upon) manual gating while improving reproducibility and reducing operator bias. The authors apply it across multiple cytometry platforms and validate its performance using transcriptomic data and comparisons to other methods. The overall concept is well-motivated and the results are promising. I found the work to be thoughtfully executed, though I have several suggestions that could help clarify certain aspects and strengthen the evaluation.

Major suggestions:

The heuristic for selecting marker pairs, based on differences in distribution percentiles between target and non-target populations, is intuitive and computationally efficient. However, it may overlook more informative combinations. Including a brief comparison to alternative selection criteria, such as mutual information or other discriminative metrics, could help validate the robustness of this choice.

We thank the reviewer for the insightful suggestion. To evaluate the robustness of our heuristic marker selection method, we compared the resulting marker rankings with those obtained from two commonly used discriminative models: a decision tree and a support vector machine (SVM). These models were chosen for their differing strengths, making them useful baselines for comparison:

- Decision trees capture hierarchical and non-linear decision rules.

- SVMs identify an optimal separating hyperplane.

We computed Spearman rank correlations between the marker rankings derived from our heuristic and those obtained from the tree and SVM classifiers, using the Oetjen et al. dataset described in the manuscript. This analysis was performed across all cell types and three levels of cell type annotation. As shown in the new **Supplementary Figure 1**, the heuristic demonstrated consistent and positive correlations with both classifiers, particularly with the SVM (median $\rho > 0.57$ across levels). Notably, the correlation between the heuristic and the SVM was higher than that between the decision tree and the SVM, suggesting that our method prioritizes markers in a manner more similar to sophisticated approaches like SVMs, and performs within their range.

Importantly, since our approach to constructing gates involves intersecting decision boundaries that resemble separating hyperplanes, conceptually closer to SVMs than to tree-based partitioning, we find this agreement particularly encouraging. It further supports that our heuristic identifies biologically and geometrically meaningful markers, while remaining computationally efficient. The median computation time for our heuristic was below 0.35 seconds across all annotation levels, whereas the SVM classifier required over 10 seconds for cell type annotation level 2, over 20 seconds for cell type annotation level 3, and over 40 seconds for cell type

annotation level 4. In the revised version of the manuscript, we added a **new Supplementary Figure 1** and updated the methods and results sections to include this comparison.

In results:

When comparing marker rankings from this heuristic method with those from tree-based and SVM-based classifiers, we observed overall positive correlations, with the heuristic and SVM rankings being more strongly correlated than those from the tree-based approach (**Supplementary Fig. 1**). The heuristic method also demonstrated the shortest computation time (**Supplementary Table 1**).

In methods:

Optional alternative marker selection strategies are implemented, in which either a shallow decision tree (max depth = 4) or a class-balanced linear SVM is trained on each marker independently to separate the target from the non-target population. Markers are then ranked by the resulting F1 scores, and the top two markers are selected for the corresponding hierarchy.

Added paragraph:

Comparison of heuristic and classifier-based marker selection

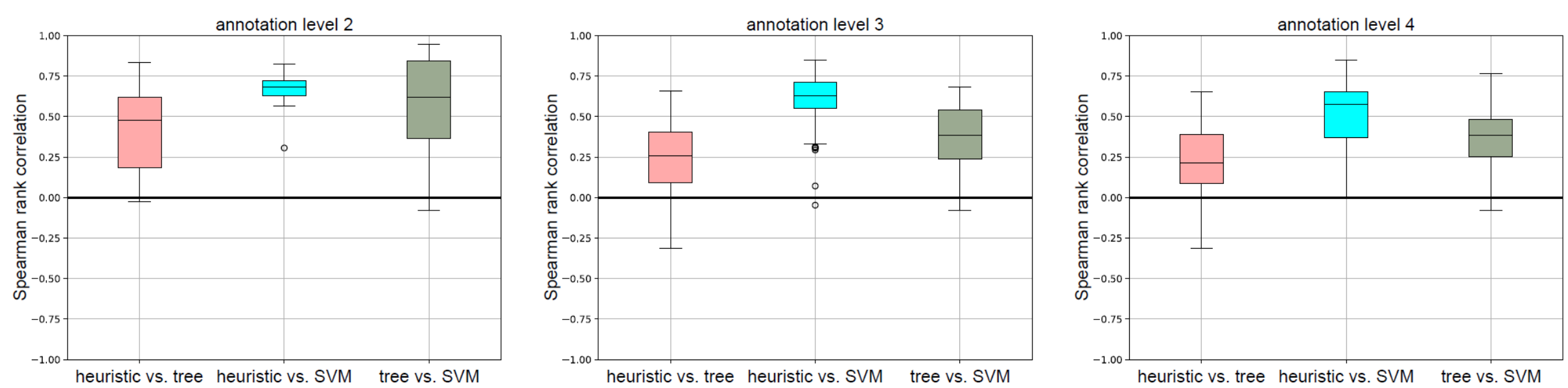

Supplementary Figure 1. Comparison of heuristic marker selection with feature importance from classifiers. Boxplots showing Spearman rank correlations between the rankings of marker importance derived from the heuristic marker selection, a tree-based classifier, and an SVM classifier. Results are shown for all cell types across all samples in the human bone marrow cyTOF data set at annotation level 2 (left), annotation level 3 (middle), and annotation level 4 (right).

The authors show that there is considerable variability in human-labeled gating and position ConvexGating as a viable, standardized, algorithmic alternative. However, the manuscript does not include a direct comparison of ConvexGating's performance against human gating in reproducing a gold standard. Including such a comparison would provide stronger support for the claim that ConvexGating can effectively replace or standardize manual gating workflows.

We thank the reviewer for this insightful comment that highlights an important distinction in our positioning of ConvexGating. The reviewer correctly identifies that we demonstrate human

gating variability but do not provide a direct head-to-head comparison against a Gold standard. This reflects a fundamental difference in ConvexGating's intended purpose: rather than attempting to replicate or improve upon manual gating decisions, ConvexGating aims to facilitate clustering-based cell type identification workflows by making them compatible with cell sorting instrumentation.

We think that establishing a true "Gold standard" for gating strategies is inherently difficult, as evidenced by the inter-operator variability we document (**Figure 3** and **Supplementary Figure 4**). Manual gates often represent expert interpretation that may vary based on experimental context, biological knowledge, and analytical goals. Our tool addresses a different workflow entirely - it translates clustering-identified cell populations into cell sorting-compatible gates. The relevant comparison is not whether ConvexGating matches manual gating decisions, but whether it accurately captures clustering-defined populations in a format suitable for cell sorting. We acknowledge this represents a limitation in directly validating ConvexGating against traditional gating approaches for cell sorting. This is why we conducted two validation experiments of flow-sorted TEMRA CD8 T cells and progenitor populations of white fat tissue (see our response to this reviewer's comment below), respectively, followed by single-cell RNAseq to compare a conventional gating strategy vs ConvexGating inferred gating strategy. In both cases, we achieved higher precision (population purity) with ConvexGating compared to a conventional gating strategy.

In our revised version of the manuscript, we have emphasized that ConvexGating is positioned as an enabler for alternative workflows rather than a direct replacement for manual gating expertise (see our response to **Reviewer 1 major comment 1** and **revised Figure 1 A**).

Most of the validation focuses on immune populations, particularly CD8+ T cells, and primarily within peripheral blood or related contexts. While these are important use cases, it would be helpful to assess whether ConvexGating generalizes to a broader range of tissues and cell types, including non-immune or more heterogeneous populations. This would better demonstrate its applicability across diverse biological settings.

We thank the reviewer for this valuable suggestion. We acknowledge that the manuscript previously focused predominantly on immune cells. To demonstrate ConvexGating's ability to robustly transfer to non-immune cells, we performed an additional wet lab experiment using visceral white adipose tissue (WAT), a technically challenging tissue for cell sorting due to its high lipid content and cellular fragility. We stained WAT cells and sorted two adipocyte precursor subpopulations (P1 and P2), which we then isolated for plate-based single-cell RNA-seq with FLASH-Seq to confirm their identities (schema in **new Supplementary Figure 10 A**). All antibodies that were used in the index-sort experiment are listed in **Supplementary Table 3**.
First, we conducted a test experiment to determine optimal parameters for the new panel and for both conventional and ConvexGating strategies. Running the tool on precursor populations P1 and P2 yielded the gating strategy displayed in **Supplementary Figure 10 B**, with the P2 population showing higher precision and recall than P1. Transfer of the respective strategies to the cell sorter as well as the conventional gating strategy are shown in **Supplementary Figure 11 C**.

After index-sorting both precursor subpopulations (**Supplementary Figure 11 A**) and subsequent scRNA-seq, we performed PCA, neighbor calculations, and clustering (**Supplementary Figure 11 B**). Using the respective cell type markers, we annotated both sorted cell types (**Supplementary Figure 11 C-G**). The contingency tables in **Supplementary Figure 11 D** revealed that P1 was challenging to sort with either strategy, whereas P2 showed better results. Purity for P2 was high with both strategies, with ConvexGating performing slightly better (**Supplementary Figure 11 E**). In conclusion, this additional experiment demonstrates that ConvexGating successfully transfers to non-immune cells and maintains robust performance even in technically challenging tissues such as WAT.

In the revised version of the manuscript, we have added these data to the results section and as **new Supplementary Figure 10** and **new Supplementary Figure 11**. We would like to thank the reviewer again for allowing us to strengthen our manuscript based on this suggestion.

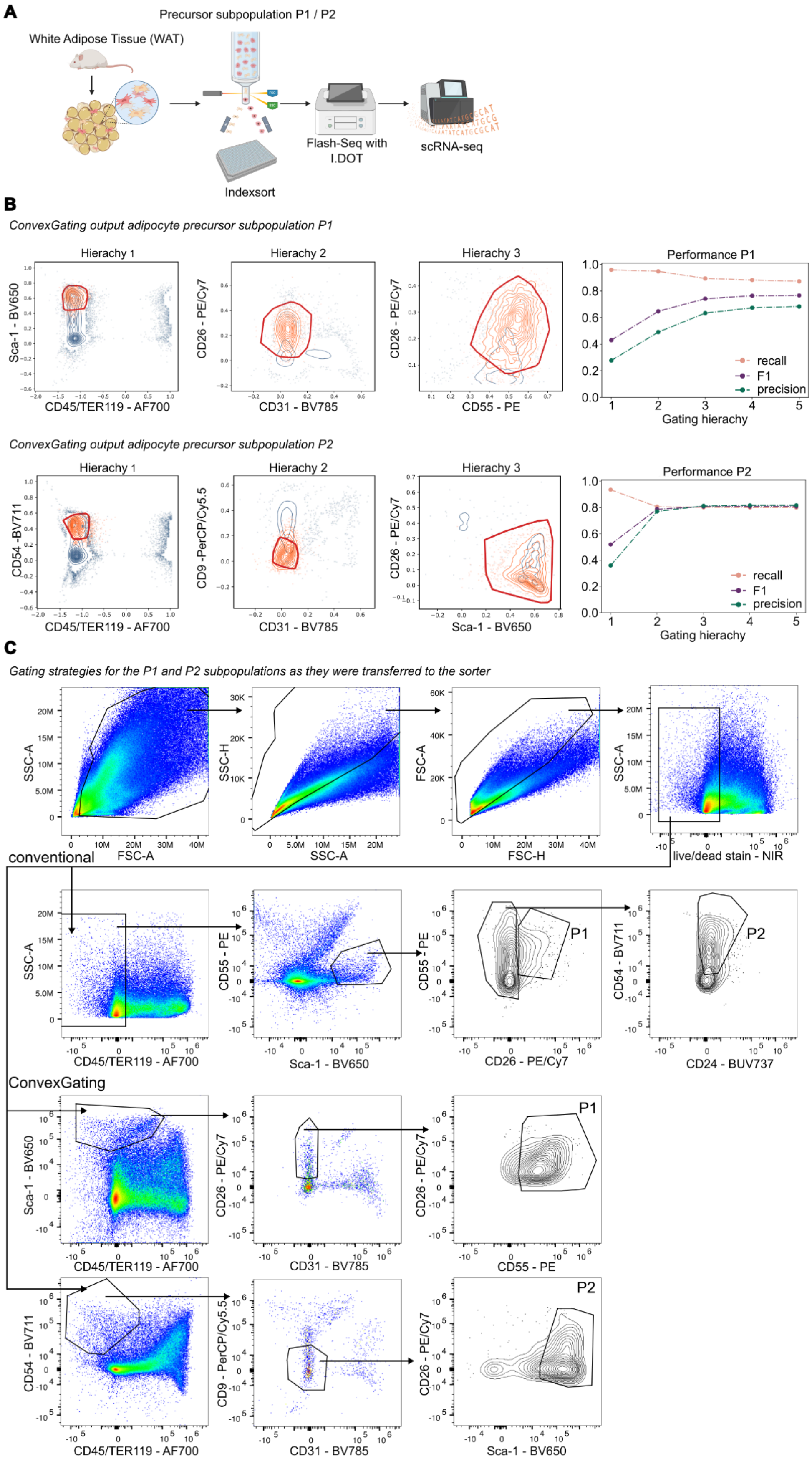
A
Precursor subpopulation P1 / P2
White Adipose Tissue (WAT)
Flash-Seq with I.DOT
scRNA-seq
Indexsort
B
ConvexGating output adipocyte precursor subpopulation P1
Hierachy 1
Hierachy 2
Hierachy 3
Performance P1
Sca-1 - BV650
CD45/TER119 - AF700
CD26 - PE/Cy7
CD31 - BV785
CD55 - PE
recall
F1
precision
Gating hierachy
ConvexGating output adipocyte precursor subpopulation P2
Performance P2
CD54 - BV711
CD9 -PerCP/Cy5.5
Sca-1 - BV650
C
Gating strategies for the P1 and P2 subpopulations as they were transferred to the sorter
SSC-A
FSC-A
SSC-H
FSC-H
live/dead stain - NIR
conventional
CD55 - PE
CD26 - PE/Cy7
P1
P2
CD24 - BUV737
ConvexGating
CD9 - PerCP/Cy5.5
CD31 - BV785

Supplementary Figure 10. Gating on White Adipose Tissue precursor cells with ConvexGating and a conventional gating strategy. (**A**) Schematic overview of the experimental design used to study adipocyte precursor subpopulations P1 and P2 isolated from white adipose tissue (WAT) of a high-fat diet mouse. (**B**) ConvexGating results for target populations P1 (upper panel) and P2 (lower panel). (**C**) Gating strategy implemented on the FACSDiscover8 for index-sorting adipocyte precursor populations P1 and P2 into 384-well-plates. Initial quality control gates (size, doublet exclusion and live cell filtering) were shared between the two strategies (row 1). Conventional gating strategies for P1 and P2 are shown in row 2, while ConvexGating-derived strategies for P1 and P2 are displayed in rows 3 and 4, respectively.

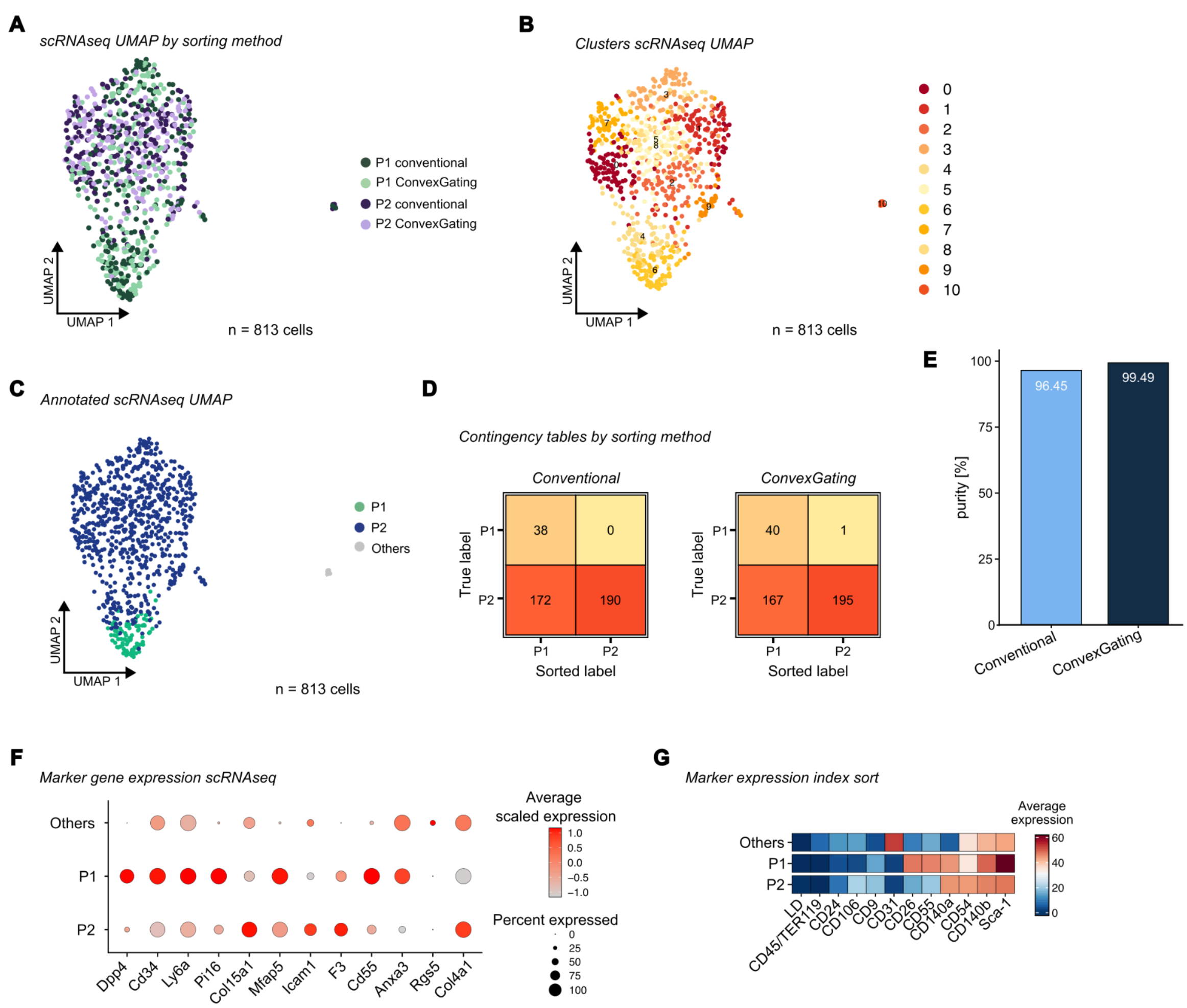

Supplementary Figure 11. Single-cell RNA sequencing validates adipocyte precursor population identities. (**A**) UMAP of adipocyte precursor populations P1 and P2 colored by index-sort labels (P1 and P2) of the respective gating strategies (ConvexGating and

conventional). (**B**) Clustering of scRNA data from sorted white adipocyte tissue precursor cells. (**C**) Annotated UMAP of P1 and P2 precursor populations based on scRNA-seq analysis. (**D**) Contingency tables comparing flow cytometry sort labels with scRNA-seq-derived cell type annotations for conventional and ConvexGating strategies. scRNA-seq labels are annotated as true labels. (**E**) P2 population purity achieved by conventional versus ConvexGating strategies. (**F**) Scaled expression heatmap of marker genes distinguishing Others, P1, and P2 clusters. (**G**) Surface marker protein expression levels across the three annotated cell populations, as measured by index sorting.

Minor suggestions:
Figure 1 would benefit from a clearer schematic illustrating how the convex gates evolve during training.

We thank the reviewer for this suggestion. In the revised manuscript, we have **updated Figure 1 B** (see **Reviewer 1 major comment 1**) to include a clearer schematic that better illustrates how the convex gates evolve during training.

It would be helpful to provide an estimated runtime for ConvexGating on a typical dataset (e.g., 50k cells with 30 markers).

We thank the reviewer for their interest in the runtime of ConvexGating. In a typical dataset with 50,000 cells and 30 markers, we recorded runtimes of approx. 2 min per target cell type. We added this information to the GitHub repository of ConvexGating.

Reviewer #3 (Remarks on code availability):

Code is well documented

Thank you for your positive feedback on the code documentation.

REVIEWER COMMENTS

Reviewer #2 (Remarks to the Author):

In this revised manuscript, Friedrich et al. have substantially improved the presentation of their results, notably by strengthening the quantitative evaluations of their method by clearly evaluating on held out cells from the same sample, cells from a distinct sample but generated in the same experimental fashion, or on data from a distinct and independently generated sample. The authors have also satisfyingly adressed most of my previous concerns or corrected me in some instances.

I am however still unconvinced by two of their answers to my previous comments.

1.My previous point #2 was:

“In many instances, it seems that markers are selected (with the heuristic “step 1” described in the method) but end up having little influence on the final output. For instance, when gating on pDCs in fig 3h, the authors report that ConvexGating identified CD57-CD123+CD13-CD45RA+ as the phenotype of pDCs. But when examining Fig 3H, it appears that CD57- has no effect to gate on pDCs since CD123+ cells are all CD57- ? Worryingly, the contribution of CD57 appears to be mostly to gate out CD123+ events that are below the axis for CD57 intensity. This suggests that CD57 does not meaningfully, but rather artifactually, contribute to the identification of pDCs in this instance. “

The authors replied by showing that removing CD57 indeed helped ConvexGating identify a gating strategy with higher performance. The way I see it in this example CD123+ cells are way scarcer than CD123- cells and the CD57+ gate is a way for the model to account for that, although examination of figure 3H clearly shows pDCs are CD57-. My argument was that in this instance ConvexGating identified CD57 as a discriminating marker for pDCs when it was not, and the manual exclusion analysis that the authors performed seems to confirm it. I thus think this warrants a discussion on the performance of this approach and that marker selection potentially needs to be refined manually ?

We thank the reviewer for pressing on this point, and we agree with his interpretation. In this instance, CD57 was selected because CD123+ cells are predominantly CD57-, meaning CD57- provides geometric separation that partially mirrors CD123+ in the feature space. We would also note that which markers are selected as most discriminating is inherently dependent on the specific panel composition and the concrete dye and antibody combinations used, hence, marker selection by ConvexGating is contextual by nature.
We have added a paragraph to the Discussion explicitly acknowledging this limitation. ConvexGating optimizes for geometric discrimination, and this can result in the selection of markers that are solely statistically separating. As with other computational gating tools, output

markers should be evaluated against domain knowledge, and the tool is expressly designed to support this kind of iterative, user-guided refinement. The pDC example illustrates this workflow well: the algorithm's initial solution serves as a starting point, and systematic exclusion of biologically implausible markers yields a more interpretable result. It is also important to note that ConvexGating was designed primarily for cell sorting applications where sample purity as well as retrieval are critical. ConvexGating optimises the F1 score with precision-focus by default. However, a recall-focused mode is available and when re-running ConvexGating with the focus parameter set to "recall", ConvexGating again selected CD57 as part of the optimal strategy, confirming the previously defined CD57- CD13- CD45RA+ CD123+ phenotype (**Reviewer Figure R1**). This is consistent with the established definition of human pDCs and suggests that, despite being a statistically redundant marker in this context, CD57 does not introduce biological error and the resulting gate correctly captures the population of interest. Nonetheless, we agree that its selection warrants transparency, and the Discussion has been revised accordingly (lines 449-456):

"As ConvexGating is designed to optimize marker discrimination, marker selection is primarily guided by separability in feature space and consequently is not directly aware of biological significance but rather by panel characteristics such as signal brightness and staining index. This can occasionally result in a marker selection where, for example, a negative marker is selected to identify a more biologically informative positive marker. Consequently, ConvexGating may prioritise statistically informative markers that should be evaluated against domain knowledge when available as the tool is expressly designed to support user-guided refinement"

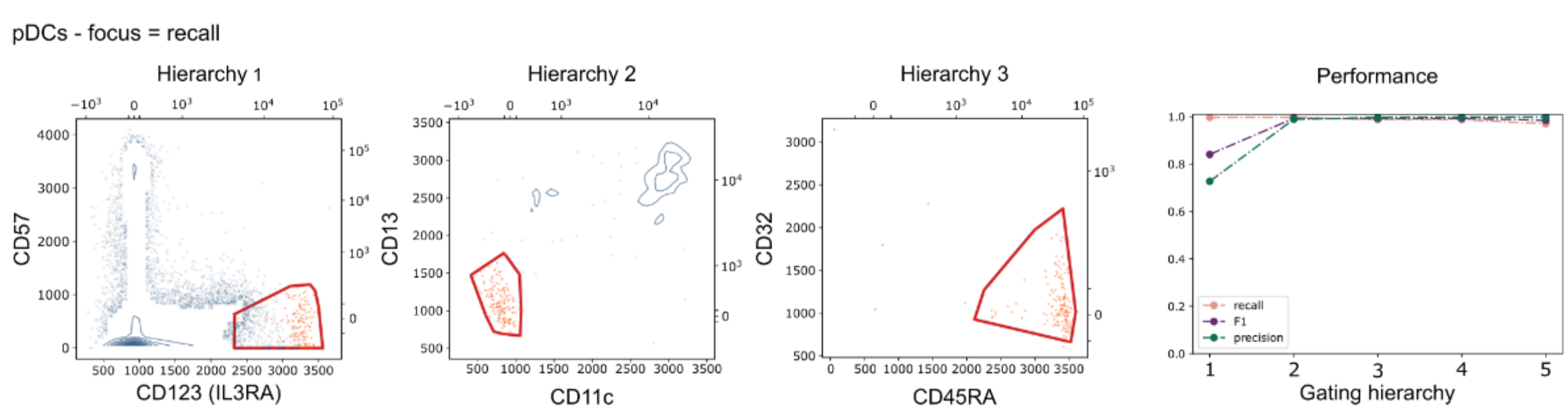

Reviewer Figure R1. ConvexGating consistently identifies pDCs with a consistent phenotype when prioritizing recall. ConvexGating was re-run on the pDC gating example with the focus parameter set to "recall" rather than the default F1-prioritized mode. The resulting gating strategy identifies pDCs as CD57- CD13- CD45RA+ CD123+, consistent with the previously established pDC phenotype shown in Figure 3H. Compared to the default mode, the recall-focused strategy captures a larger fraction of target cells at the cost of a modest reduction in precision, reflecting the inherent trade-off between population recovery and sorting purity. This demonstrates that while marker selection may vary with the chosen optimization focus, the biological definition of the population remains stable across modes, and that the choice between precision- and recall-focused strategies should be guided by the experimental context and the acceptable level of potential contamination in the sorted population.

2.My previous point #3 was :

“Additionally, the authors report to have subsetted 2,000 positive events and 2,000 negative events before running Hypergate, and state that this is the recommended procedure. I do not think this is the recommended procedure nor that this is a fair comparison. The two methods should be ran and evaluated on the exact same data.

The authors replied in their rebuttal by quoting the Hypergate paper to justify their choice.
In my opinion, metrics such as Precision and Recall will heavily depend on the balance of classes (% of positive and negative cells in this case), which I think justifies downsampling under stable proportions (as it appears to be done for ConvexGating).
Additionally, I would argue that this is what the authors of the Hypergate paper performed, as they mention “we also sampled a corresponding proportion of negative events.” (proportion, not count). I am the first author of that article so I apologize if that sentence was confusing, but I believe downsampling while maintaining class proportions is more appropriate in this instance.
In any case, I don’t think that comparing methods on datasets that were subsampled in different ways makes sense in this context, especially when the metric is F1 or its constituents.

I thus argue that the comparisons to Hypergate should be redone on the same data subsamples.

We sincerely thank the reviewer for his clarification and we appreciate him identifying himself as the first author of the Hypergate paper. We accept the provided clarification that “a corresponding proportion of negative events” indeed refers to maintaining class proportions rather than a fixed count. We further agree with the methodological argument that comparing different methods is most senseful when data is subsampled the same way. We would like to emphasize that we modified the benchmark in the previous round of revisions using the exact same data subsamples in all methods in our previous submission and we apologize that we failed to emphasize this crucial point more clearly in our previous response.
In the method section describing “Benchmark Gating Strategies”, we clarified the use of generation of training and test data as follows (lines 816-823):

For executing hypergate, we made use of the most recent hypergate R package version 0.8.3 (https://github.com/ebecht/hypergate) in R v. 4.0.3. For all comparisons, we first created train and test splits of all datasets such that all methods were run on the exact same data. To enable sufficient coverage of granular target cell types, we constructed training datasets with a fixed 1:1 ratio of target to non-target cells, using the remaining data as the test set. The training data included 70% of the available target cells, with a maximum total of 50,000 cells. When 70% of the target cells were insufficient to reach the specified 1:1 ratio, fewer cells were used for training to preserve this balance.

We also repeated the benchmarking using the same data subsamples across both methods, with cell type proportions preserved (**Reviewer Figure R2**). We acknowledge the reviewer's opinion that precision and recall will heavily depend on the balance of classes. However, the cell type compositions vary such that maintaining the class proportion in subsampling leads to lower performance comparability across cell types.

This is further supported by the updated benchmarking results using identical data subsamples with preserved cell type proportions (**Reviewer Figure R2**): ConvexGating still outperformed Hypergate in terms of precision in all but one scenario, while Hypergate performed better in F1 score and recall.

Importantly, while SVM classifiers provide superior performance, they cannot be directly translated into experimental sorting strategies due to their reliance on high-dimensional feature spaces. ConvexGating's constraint to 2D marker spaces, though limiting in some scenarios, is precisely what enables its practical utility for flow cytometry.

We would like to emphasize, however, that fixing cell type proportions does not fully reflect real-world usage of ConvexGating, especially for rare cell types. In practice, users would typically oversample rare target cell types when running ConvexGating, a scenario that is more favorable to ConvexGating's precision-focused design.

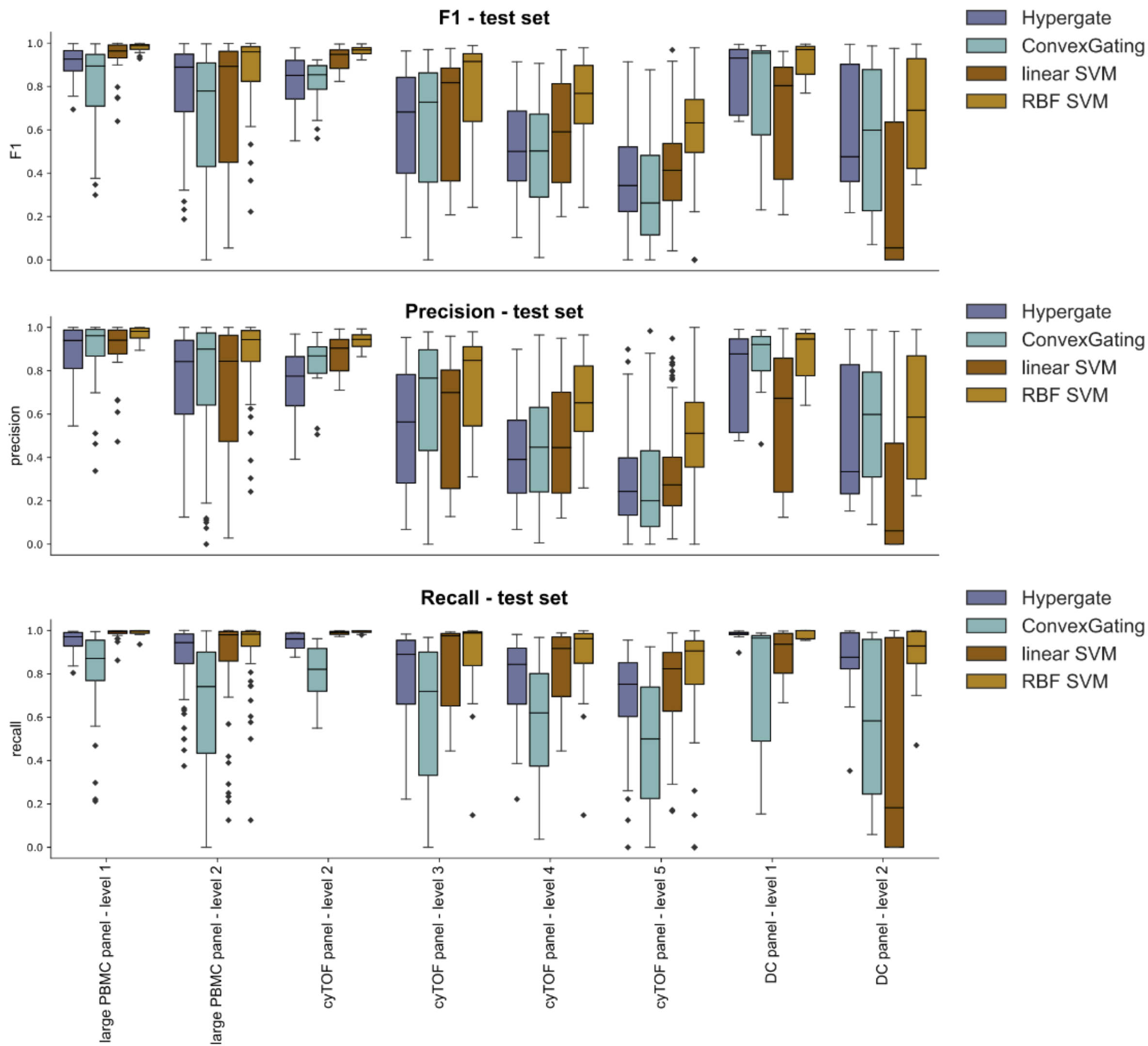

Reviewer Figure R2. Computational benchmark analysis comparing gating strategies from ConvexGating and Hypergate with machine learning classifiers on identical data subsamples with preserved cell type proportions. F1 score (upper panel), precision (middle panel), and recall (lower panel) boxplot results on the test set for gating strategies learned by ConvexGating and Hypergate, and for classification performance of linear SVM and RBF SVM models. Boxes represent the 25th percentile, median, and 75th percentile, respectively; whiskers indicate 1.5× the interquartile range, and black diamonds denote outliers. The benchmark analysis includes the large PBMC panel with two cell type annotation levels, the human bone marrow cyTOF panel with four annotation levels, and the DC panel with two annotation levels. All tools were run on identical data and fixed cell type proportions.

3.In my previous Minor points #5 I was pointing at a typographical error that the authors could not find. I apologize if my comment lacked clarity, the sentence that needs fixing is in the “Isolation of human PBMCs to sort CD8+ naive and CD8+ TEMRA cells” section of methods, specifically the sentence “We isolated PBMCs of one healthy donor from EDTA-treated peripheral blood via Pancoll density centrifugation at 800 xg for 20 minutes at room temperature without break”. I was saying that it should likely be “without brake” and not “without break” although this is not my main area of expertise.

Otherwise, the authors satisfyingly adressed my comments. The two major points above still need to be discussed or redone.

Etienne Becht

We thank the reviewer for his patience in clarifying this point and apologize for missing it previously. This is absolutely correct, the intended phrasing is “without brake”, referring to the deceleration brake of the centrifuge which is intentionally disabled during density gradient centrifugation. We have corrected the typo in the re-revised manuscript (line 538):

"Isolation of human PBMCs to sort CD8+ naive and CD8+ TEMRA cells

We isolated PBMCs of one healthy donor from EDTA-treated peripheral blood via Pancoll density centrifugation at 800 xg for 20 minutes at room temperature without brake. Ethical approval was obtained from the Ethics Committee of the University of Bonn under the reference 272/23-EP. Cells were collected with a Pasteur pipette and washed twice with MACS buffer (1x PBS with 0.5% BSA and 0.074% EDTA). Then, we stained the cells with the surface antibodies listed in **Supplementary Table 2** at 4°C for 30 minutes. We washed twice and proceeded with FACS sorting."

Reviewer #3 (Remarks to the Author):

The authors sufficiently addressed all my comments.

Reviewer #4 (Remarks to the Author):

I sincerely appreciate the authors' great revision work. The authors have successfully addressed most of the concerns proposed by Reviewer 1. However, here are some points that are not fully discussed.

#1: Where does ConvexGating’s main strength lie as compared to other methods?
Many clustering (unsupervised) and auto-gating (supervised or semi-supervised) methods are available for mass and cytometry data gating. Some of these methods apply machine learning approaches and employ markers (prior knowledge) for cell sorting. What are the major novelties and strengths of the proposed ConvexGating? Does ConvexGating present superior performance when quantitatively compared with existing methods? I believe this should be the major information that is expected to be highlighted.

We thank the reviewer for this question, as it allows us to clarify the intended positioning of ConvexGating more explicitly.

ConvexGating is not designed as a general-purpose flow cytometry analysis tool, and we do not claim it is universally superior to existing methods. Its core novelty lies in solving a specific problem: translating cluster-based annotations from high-dimensional cytometry data into actionable, instrument-executable gating strategies for cell sorting. Methods such as FlowSOM or Phenograph excel at clustering cells for subsequent labeling in silico, but their outputs cannot be directly implemented on a cell sorter. ConvexGating bridges this gap by converting any cluster annotation into a hierarchy of 2D polygon gates executable on a sorting instrument - a capability that, to our knowledge, no existing tool addresses directly. It is therefore intended to be used downstream of existing clustering workflows, not as a replacement for them.

That said, we did perform a direct quantitative comparison with Hypergate, the most closely related existing tool (compare lines 308-318 and **Supplementary Fig. 8**). ConvexGating outperformed Hypergate in mean F1 score and precision across all annotation levels of the different datasets, with ConvexGating focused on high precision while Hypergate prioritized recall. This makes ConvexGating better suited for applications where achieving a high level of purity is critical.

We also highlighted ConvexGating's intended position within a typical cytometry analysis workflow in the graphical abstract in **Figure 1A**:

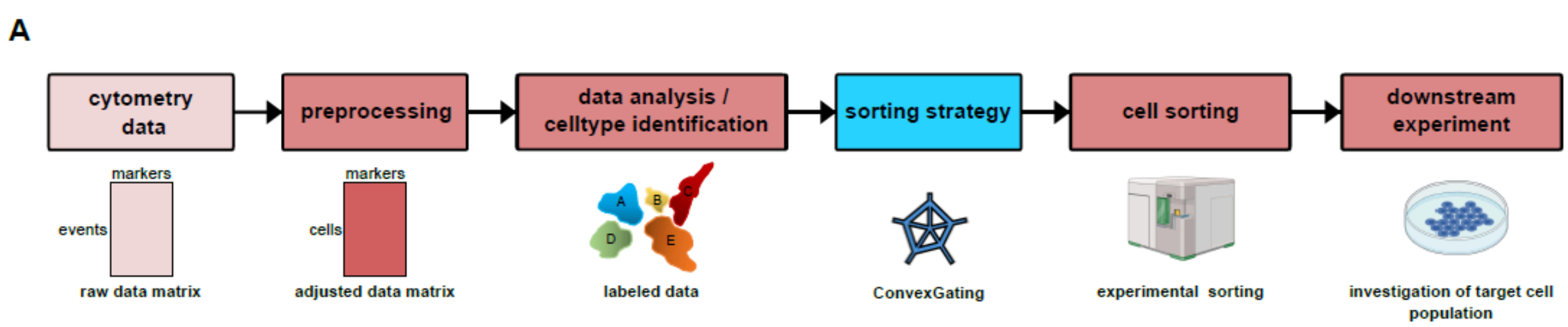

We have also revised the abstract and introduction to make ConvexGating's intended niche and relationship to existing methods clearer throughout:

Abstract (lines 44-49):

Cluster-based approaches can leverage the full marker space to define cell type populations with greater consistency, but their outputs cannot be directly implemented on a cell sorter, making validation and investigation of novel cell types or cell states challenging. Here, we propose ConvexGating, an AI tool that bridges this gap by automatically learning gating strategies for sorting in an unbiased, fully data-driven, yet interpretable manner.

Introduction (lines 139-142):

Importantly, ConvexGating is designed to complement existing clustering or annotation tools by converting cluster-based cell type annotations into a hierarchy of 2D polygon gates that can be directly executed on a cell sorter. This closes the gap between computational analysis and physical cell isolation.

Reviewer #4 (Remarks on code availability):

The README page is clear, with a user-friendly example demo.

Thank you for your positive feedback regarding the code documentation.

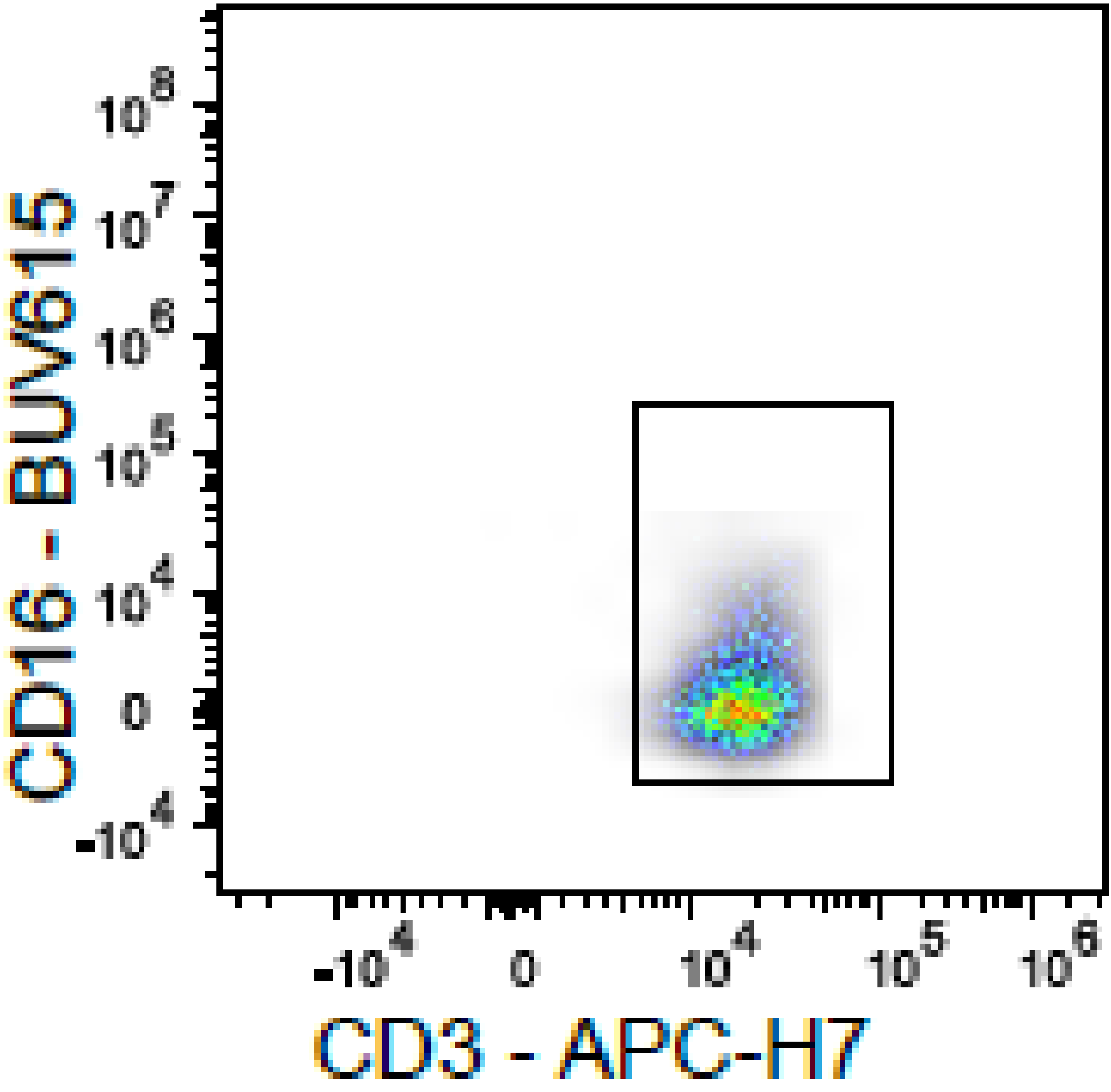

CD16 - BUV615
10^8
10^7
10^6
10^5
10^4
0
-10^4
-10^4
0
10^4
10^5
10^6
CD3 - APC-H7
